# Real-world outcomes of concomitant antidepressant and statin use in primary care patients with depression: a population-based cohort study

**DOI:** 10.1186/s12916-023-03138-5

**Published:** 2023-11-07

**Authors:** Riccardo De Giorgi, Franco De Crescenzo, Philip J. Cowen, Catherine J. Harmer, Andrea Cipriani

**Affiliations:** 1 Department of Psychiatry, University of Oxford, Warneford Hospital, Warneford Lane, Oxford, OX3 7JX UK; 2 Oxford Health NHS Foundation Trust, Warneford Hospital, Warneford Lane, Oxford, OX3 7JX UK; 3 Oxford Precision Psychaitry Lab, Oxford Health Biomedical Research Centre, University of Oxford, Oxford, UK

**Keywords:** Depression, Antidepressant, Statin, Primary care, Epidemiology, Real-world effects, Cohort study, QResearch, Treatment adherence, Antidepressant efficacy

## Abstract

**Background:**

Antidepressants are licensed for use in depressive disorders, but non-response and poor adherence to treatment affect a considerable number of patients. Pre-clinical and clinical evidence suggest that statins can augment the effects of antidepressants. However, the acceptability and tolerability of combining statins with antidepressants are unclear, and their add-on efficacy has only been shown in small, short-term clinical trials. Observational data can provide complementary information about treatment effects on larger samples over longer follow-ups. In this study, we therefore assessed the real-world acceptability, tolerability, and efficacy of concomitant antidepressant and statin treatment in depression.

**Methods:**

We conducted a population-based cohort study investigating QResearch primary care research database, which comprises the anonymised electronic healthcare records of 35 + million patients over 1574 English general practices. Patients aged 18–100 years, registered between January 1998 and August 2020, diagnosed with a new episode of depression, and commencing an antidepressant were included. Using a between-subject design, we identified two study groups: antidepressant + statin versus antidepressant-only prescriptions.

Outcomes of interest included the following: antidepressant treatment discontinuations due to any cause (acceptability) and due to any adverse event (tolerability) and effects on depressive symptoms (efficacy) measured as response, remission, and change in depression score on the Patient Health Questionnaire-9. All outcomes were assessed at 2, 6, and 12 months using multivariable regression analyses, adjusted for relevant confounders, to calculate adjusted odds ratios (aORs) or mean differences (aMDs) with 99% confidence intervals (99% CIs).

**Results:**

Compared to antidepressant-only (*N* 626,335), antidepressant + statin (*N* 46,482) was associated with higher antidepressant treatment acceptability (aOR_2months_ 0.88, 99% CI 0.85 to 0.91; aOR_6months_ 0.81, 99% CI 0.79 to 0.84; aOR_12months_ 0.78, 99% CI 0.75 to 0.81) and tolerability (aOR_2months_ 0.92, 99% CI 0.87 to 0.98; aOR_6months_ 0.94, 99% CI 0.89 to 0.99, though not long term aOR_12 months_ 1.02, 99% CI 0.97 to 1.06). Efficacy did not differ between groups (range aOR_2-12 months_ 1.00 and 1.02 for response and remission, range aOR_2-12 months_ − 0.01 and − 0.02 for change in depression score).

**Conclusions:**

On real-world data, there is a positive correlation between antidepressant treatment adherence and statin use, partly explained by fewer dropouts due to adverse events. The main limitation of our study is its observational design, which restricts the potential to make causal inferences.

**Supplementary Information:**

The online version contains supplementary material available at 10.1186/s12916-023-03138-5.

## Background

Depression affects more than 350 million people worldwide and is associated with a substantial clinical, economic, and societal burden [1]. Clinical guidelines endorse the use of antidepressants for the treatment of adults with moderate to severe depression [2–6]. Nonetheless, only about 50% of these patients respond to first-line antidepressants, and one third still suffer from impairing depressive symptoms after four steps of treatment over 1 year [7]. Importantly, more than 25% discontinue antidepressants due to any cause, and one tenth suspend treatment because of intolerable side-effects after only 2 months [8].

Evidence from pre-clinical [9–12] and clinical [13–16] studies suggests that statins are promising candidates to repurpose for the treatment of depressive disorders, especially when combined with conventional antidepressant medications [17–20]. A meta-analysis of randomised placebo-controlled trials in participants with depression has shown that statins, in addition to any antidepressant treatment, are more efficacious than an antidepressant plus placebo in reducing depressive symptoms at 2 months [*N* 238, standardised mean difference (SMD) − 0.48, 95% confidence interval (CI) − 0.74 to − 0.22] [18]. Comparable acceptability and tolerability between the two arms were noted, but the certainty of this finding was poor because of the very low number of events reported over a short follow-up period [18].

Indeed, clinical trials are usually powered to assess efficacy, but less so for other outcome measures [21]: small sample sizes, short follow-ups, and highly selected populations can limit the transportability and generalisability of their findings to clinical practice [22]. Observational studies that follow methodological principles shared with clinical trials (e.g. clear eligibility criteria and treatments assignments, defined start and end of follow-up, availability of detailed information about confounding variables, outcomes measures in line with those of experimental investigations, pre-specified data-analysis plan) can address these limitations and complement the evidence provided by randomised data [23]. In this population-based cohort study, we therefore investigated the real-world effects on antidepressant treatment discontinuations and depressive symptoms associated with antidepressant plus statin versus antidepressant alone in patients with depressive disorders on QResearch, the largest UK-based primary care database.

## Methods

This study has been independently reviewed and approved by the QResearch scientific committee: 18/EM/0400. The study-specific protocol is in Additional file, A1.

### Setting

QResearch primary care research registry comprises the anonymised electronic healthcare records of over 35 million patients registered with 1574 general practices in England [24]. Information on QResearch includes patients’ demographics (e.g. year of birth, gender, socio-economic status), characteristics (e.g. height, weight, smoking status), symptoms and adverse events, clinical diagnoses, and prescribed medications. To obtain consent to provide data, QResearch uses the Egton Medical Information Systems, a major supplier of primary care computer systems commissioned for most general practices across the country.

### Population

Firstly, we ascertained an open cohort of patients from eligible English general practices between the 1 January 1998 and 15 August 2020. Patients were included if they had been registered with the general practice for at least 12 months. We used previously validated [25, 26] ‘Read codes’ to identify patients with a diagnosis of depressive disorder (Additional file, A2) and applied pre-specified inclusion/exclusion criteria (reported in detail in Additional file, A3). Our final cohort included adult patients (i.e. 18–100 years old) with a first episode of depression who had started an antidepressant (follow-up initiation) and were followed up for 12 months.

### Exposure and comparison

The exposure of interest was the use of any statin at the same time of the prescription of any antidepressant monotherapy (antidepressant + statin) compared to the use of any antidepressant alone (antidepressant-only)—drugs licensed in the UK according to the British National Formulary (Additional file, A4).

### Outcomes

Effects on antidepressant treatment discontinuations (i.e. acceptability, tolerability—primary outcomes) and on depressive symptoms (i.e. efficacy as response, remission, change in depression score—secondary outcomes) were assessed at 2 months, 6 months, and 12 months from the initial prescription of antidepressant.

Acceptability was measured as the ratio of all-cause discontinuations from antidepressant treatment. Treatment discontinuation was defined as the following: a patient with a > 30-day gap between the end of a prescription of an antidepressant and the start of the next prescription (based on an average prescription length of 28 to 30 days), a patient switching to another antidepressant, or a patient prescribed any additional antidepressant, mood stabiliser, or antipsychotic.

Tolerability was measured as the ratio of discontinuations from antidepressant treatment within 30 days from any adverse event (Additional file, A5). Therefore, treatment discontinuation would have to occur within 30 days of an adverse event to be assumed to be a consequence of the adverse event.

Efficacy outcomes were measured via the Patient Health Questionnaire (PHQ)-9 [27] as the following: response (50% score reduction), remission (score < 5), change in depression score.

### Confounding variables

Confounders include baseline variables that have been shown to be risk factors for the outcomes and also potentially associated with the likelihood of receiving antidepressant or statin treatment, based on previous studies on QResearch [25, 26, 28]. The several suspected confounders included the following: age; sex; body mass index (BMI); year, type, and severity of depression diagnosis; deprivation status (Townsend deprivation score); smoking status; alcohol intake; ethnicity; antidepressant category; use of other drugs—for a total of 58 variables detailed in Additional file, A6.

### Statistical analysis

Analyses were conducted on Stata MP v16.0 [29] on the QResearch server. Baseline characteristics of the study cohort were examined with descriptive statistics for the total sample as well as separately for statin + antidepressant and antidepressant-only groups. Study outcomes were explored using an intention-to-treat analysis with multivariable logistic regression, clustered by general practices (using the *‘vce(cluster clustvar)’* function, which accounts for intragroup correlation within each general practice), to compute odds ratios (ORs, for dichotomous outcomes) or mean differences (MDs, for continuous outcomes) with 99% confidence intervals (99% CIs, instead of 95% CIs, to correct for multiple comparisons for the primary outcomes) for antidepressant + statin versus antidepressant-only (i.e. between-subject design), both unadjusted and adjusted (aOR, aMD) for all confounders. Multiple imputation by chained equations (using the *‘mi impute chained’* function of Stata MP v16.0) was employed to impute data when values were not available: for each imputation, 10 imputed datasets were generated, including all confounding and outcome variables, and coefficient estimates were combined using Rubin’s rule [30], under a missing-at-random assumption based on a prior study [31]. Results were separately reported for the complete case analysis (sensitivity analysis) and the full set (i.e. imputed) analysis (adjusted model—primary analysis). This decision was based on the full set analysis’ potential to mitigate bias associated with missing data [32].

In view of the expected age difference between the group on statin + antidepressant and the one on antidepressant-only, we conducted a subgroup analysis on the sample of patients aged > 65 years only. We also compared our multiple logistic regression analyses data on the full set for statins with the aOR and 99% CIs of aspirin (sensitivity analysis), to verify whether results were non-specifically associated with another medication used in a similar population.

## Results

**Fig. 1:**
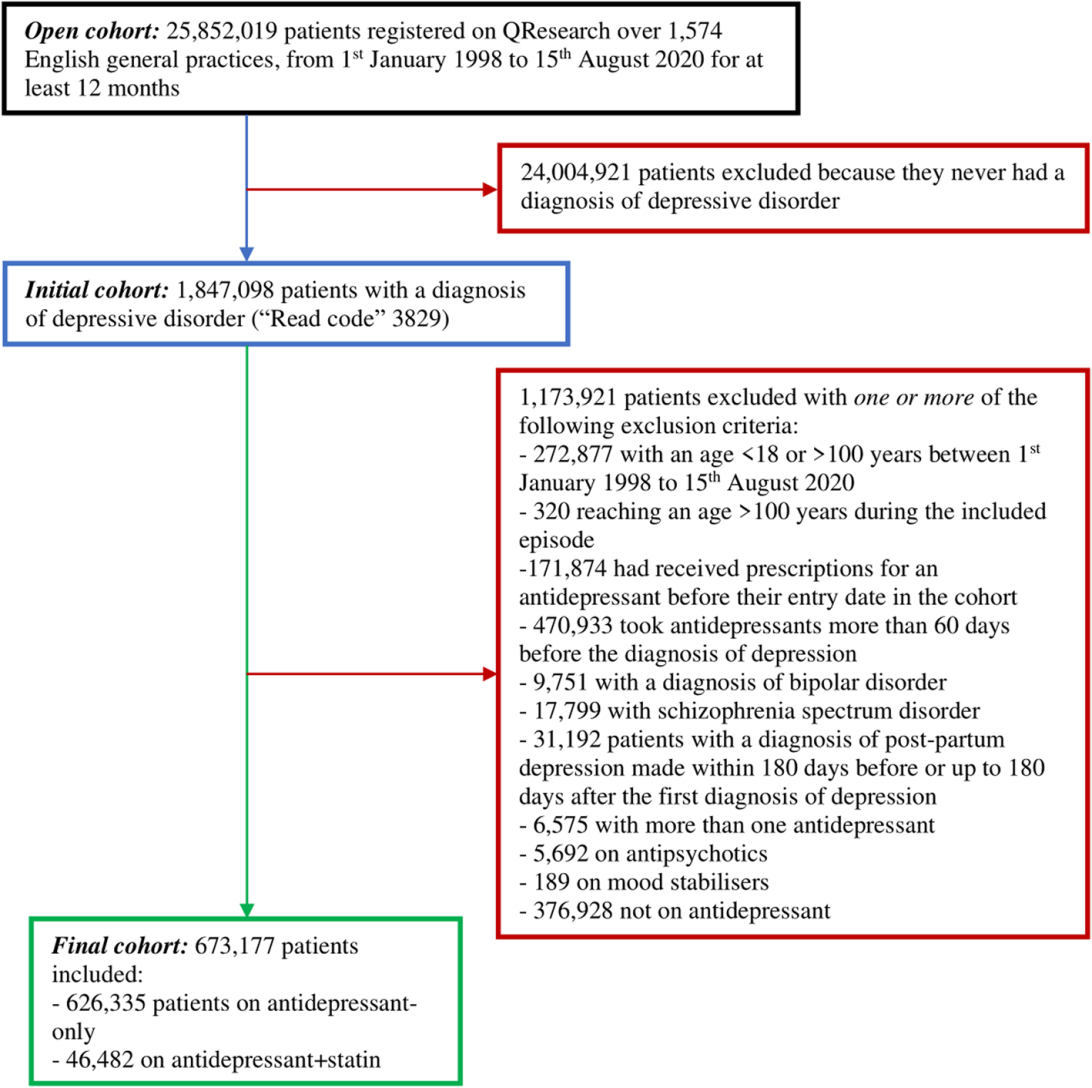
Study flow chart

From an initial cohort of 1,847,098 patients with a diagnosis of depression (‘Read code’ 3829), we applied eligibility criteria that led to the exclusion of 1,173,921 people; the most common reasons for exclusion included one or more among the following: antidepressant use before the diagnosis of depression (*N* 470,933) or the initiation of the current antidepressant (*N* 171,874), age outside the range of 18–100 years (*N* 272,877), no antidepressant use (*N* 376,928). The final cohort eventually comprised 673,177 patients: 46,482 on antidepressant + statin and 626,335 on antidepressant-only (Fig. 1).

Baseline characteristics of the study cohort are in Table 1. Overall, the sample showed moderate-severe depression (PHQ-9 17.09 ± 4.95), and the most used class of antidepressants was selective serotonin reuptake inhibitors (SSRI, 85.70%). Compared to the antidepressant-only group, patients on antidepressant + statin were older (respectively, mean age 40.9 years versus 67.1 years, see the ‘Additional analyses’ section), included more males (56.18% versus 41.90%), were more likely to have minor depression (28.18% versus 37.27%), accounted for fewer cases between 1998 and 2005 (30.44% versus 15.30%), more came from least deprived Townsend quintile (20.40% versus 24.68%), had more comorbidities (especially cardiovascular, cerebrovascular, metabolic, and neoplastic), and were taking more non-psychiatric medications. These expected baseline differences between the two groups were controlled for in the multivariable regression analyses adjusted model. Missing data, imputed for the full set analysis, are in Additional file, Table A7: data that required larger imputation due to missing values included PHQ-9 scores (78.56%), alcohol use (49.48%), ethnic group (26.72%), and body mass index (22.14%).

**Table 1:** Characteristics of study cohort at baseline

**Characteristic**	**Antidepressant-only** *N* 626,335	**Antidepressant + statin** *N* 46,482	**All sample** *N* 673,177
Mean age [SD]	40.92 (16.54)	67.13 (14.10)	42.75 (17.69)
Sex			
Male	262,431 (41.90)	26,314 (56.18)	288,745 (42.89)
Read codes for depression			
Major depression	430,221 (68.69)	27,834 (59.42)	458,055 (68.04)
Minor depression	176,528 (28.18)	17,456 (37.27)	193,984 (28.82)
Other	19,586 (3.13)	1,552 (3.31)	21,138 (3.14)
PHQ-9 baseline [SD]	*N* 133,939	*N* 10,408	*N* 144,347
	17.17 (4.92)	16.07 (5.22)	17.09 (4.95)
Year of diagnosis			
1998 to 2005	190,673 (30.44)	7,167 (15.30)	197,840 (29.39)
2006 to 2010	126,689 (20.23)	12,728 (27.17)	139,417 (20.71)
2011 to 2015	145,690 (23.26)	13,866 (29.60)	159,556 (23.70)
2016 to 2020	163,283 (26.07)	13,081 (27.93)	176,364 (26.20)
Body Mass Index [SD]	*N* 481,533	*N* 42,587	*N* 524,120
	26.01 (5.85)	28.77 (6.16)	26.24 (5.93)
Smoking (cigarettes/day)	*N* 574,590	*N* 45,707	*N* 620,297
Non-smoker	279,118 (48.58)	20,821 (45.55)	299,939 (48.35)
Ex-smoker	102,395 (17.82)	15,223 (33.31)	117,618 (18.96)
Light-smoker (1–9)	176,979 (30.80)	9,031 (19.76)	186,010 (29.99)
Moderate-smoker (10–19)	11,374 (1.98)	400 (0.88)	11,774 (1.90)
Heavy-smoker (≥ 20)	4,724 (0.82)	232 (0.51)	4,956 (0.80)
Alcohol (daily units)	*N* 311,478	*N* 28,639	*N* 340,117
Non-drinker/trivial (< 1)	170,934 (54.88)	15,722 (54.90)	186,656 (54.88)
Light (1–2)	103,715 (33.30)	8,737 (30.51)	112,452 (33.06)
Medium (3–6)	27,427 (8.81)	3,299 (11.52)	30,726 (9.03)
Heavy (7–9)	4,233 (1.36)	459 (1.60)	4,692 (1.38)
Very heavy (> 9)	5,169 (1.66)	422 (1.47)	5,591 (1.64)
Ethnic group	*N* 454,840	*N* 38,483	*N* 493,323
White	395,624 (86.98)	34,237 (88.97)	429,861 (87.14)
Indian	7,104 (1.56)	797 (2.07)	7,901 (1.60)
Pakistani	7494 (1.65)	767 (1.99)	8,261 (1.67)
Bangladeshi	4,987 (1.10)	609 (1.58)	5,596 (1.13)
Other Asian	6,674 (1.47)	531 (1.38)	7,205 (1.46)
Caribbean	5,551 (1.22)	452 (1.17)	6,003 (1.22)
Black African	8,004 (1.76)	351 (0.91)	8,355 (1.69)
Chinese	2,010 (0.44)	59 (0.15)	2,069 (0.42)
Other	17,392 (3.82)	680 (1.77)	18,072 (3.66)
Townsend deprivation score (in fifths)	*N* 624,118	*N* 46,770	*N* 670,888
1 (least deprived)	127,305 (20.40)	11,545 (24.68)	138,850 (20.70)
2	132,005 (21.15)	10,746 (22.98)	142,751 (21.28)
3	131,087 (21.00)	9,683 (20.70)	140,770 (20.98)
4	121,405 (19.45)	8,222 (17.58)	129,627 (19.32)
5 (most deprived)	112,316 (18.00)	6,574 (14.06)	118,890 (17.72)
Region of England			
East Midlands	23,054 (3.68)	1,136 (2.43)	24,190 (3.59)
East of England	23,486 (3.75)	1,742 (3.72)	25,228 (3.75)
London	130,577 (20.85)	8,469 (18.08)	139,046 (20.66)
North-East	21,708 (3.47)	1,687 (3.60)	23,395 (3.48)
North-West	122,774 (19.60)	11,283 (24.09)	134,057 (19.91)
South-Central	80,591 (12.87)	5,183 (11.06)	85,774 (12.74)
South-East	61,619 (9.84)	4,902 (10.46)	66,521 (9.88)
South-West	71,044 (11.34)	4,706 (10.05)	75,750 (11.25)
West Midlands	60,420 (9.65)	5,490 (11.72)	65,910 (9.79)
Yorkshire and Humber	31,062 (4.96)	2,244 (4.79)	33,306 (4.95)
Comorbidities at baseline			
Coronary heart disease	9,030 (1.44)	14,068 (30.03)	23,098 (3.43)
Stroke	8,934 (1.43)	8,414 (17.96)	17,348 (2.58)
Diabetes	14,784 (2.36)	14,879 (31.76)	29,663 (4.41)
Epilepsy	7,911 (1.26)	953 (2.03)	8,864 (1.32)
Hypothyroidism	16,792 (2.68)	4,076 (8.70)	20,868 (3.10)
Arthritis	32,637 (5.21)	11,411 (24.36)	44,048 (6.54)
Anxiety	73,684 (11.76)	5,230 (11.17)	78,914 (11.72)
Migraine	37,411 (5.97)	2,287 (4.88)	39,698 (5.90)
Cancer	40,233 (6.42)	8,789 (18.76)	49,022 (7.28)
Asthma	93,238 (14.89)	8,451 (18.04)	101,689 (15.11)
Renal failure	1,339 (0.21)	803 (1.71)	2,142 (0.32)
Liver failure	4,125 (0.66)	1,122 (2.40)	5,247 (0.78)
Osteoporosis	5,939 (0.95)	1,909 (4.08)	7,848 (1.17)
Suicidality	16,807 (2.68)	677 (1.45)	17,484 (2.60)
Antidepressant category at baseline			
SSRIs	539,367 (86.11)	37,569 (80.20)	oth
TCAs	43,420 (6.93)	3,160 (6.75)	46,580 (6.92)
MAOIs	77 (0.01)	8 (0.02)	85 (0.01)
Other ADs	43,471 (6.94)	6,105 (13.03)	49,576 (7.36)
Use of other drugs at baseline			
Antihypertensives	26,283 (4.20)	24,753 (52.84)	51,036 (7.58)
Aspirin	11,672 (1.86)	17,203 (36.73)	28,875 (4.29)
Anticoagulants	4,492 (0.72)	3,523 (7.52)	8,015 (1.19)
NSAIDs	22,503 (3.59)	2,466 (5.26)	24,969 (3.71)
Anticonvulsants	8,247 (1.32)	2,223 (4.75)	10,470 (1.56)
Hypnotics	47,389 (7.57)	4,788 (10.22)	52,177 (7.75)
Bisphosphonates	1,648 (0.26)	661 (1.41)	2,309 (0.34)
Contraceptives	39,530 (6.31)	143 (0.31)	39,673 (5.89)

Values are numbers (percentages) unless stated otherwise

### Study outcomes

**Fig. 2:**
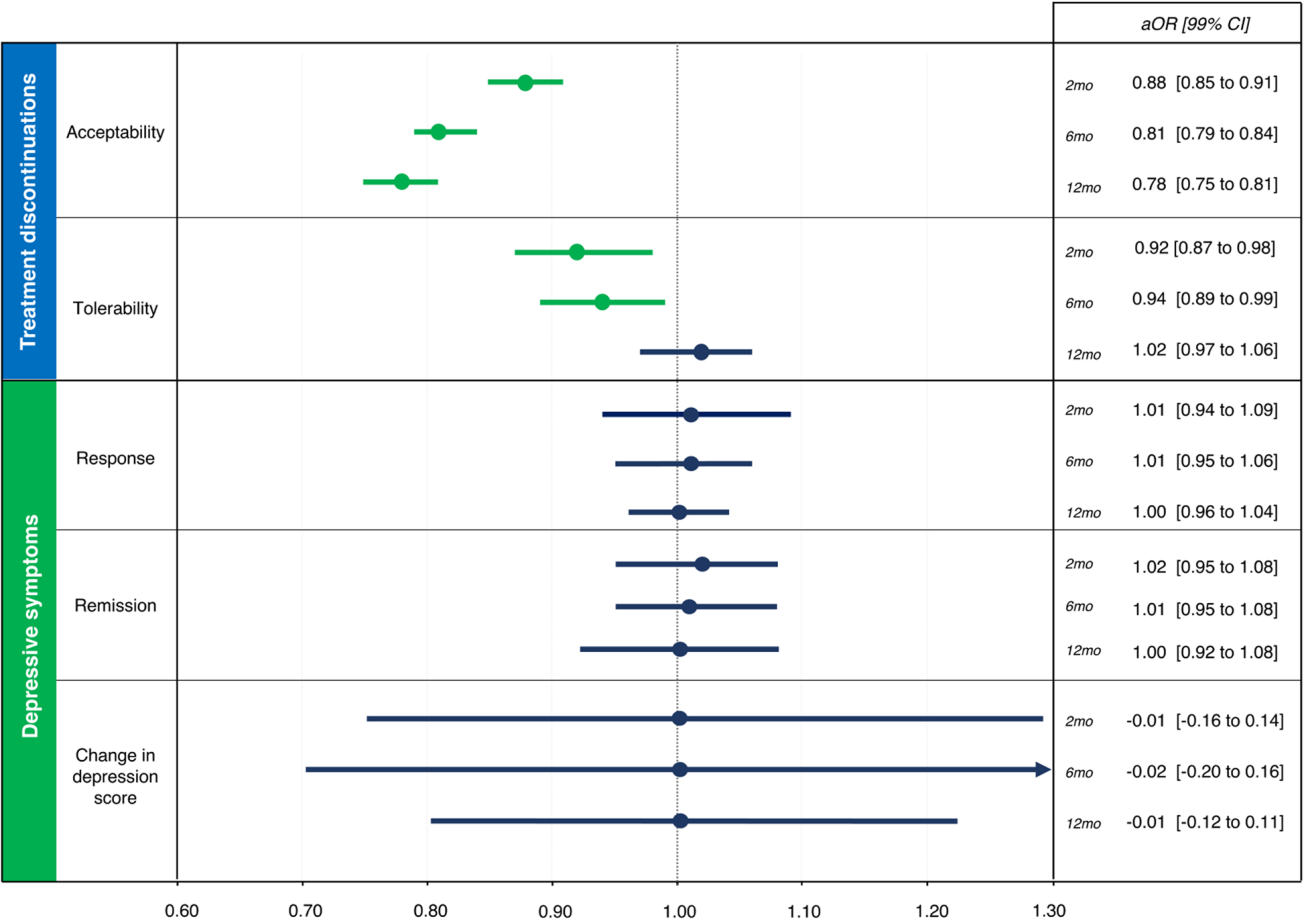
Study outcomes, full-set analysis, adjusted. Green indicates a positive effect, red indicates a negative effect, blue indicates no effect. 2mo: 2 months, 6mo: 6 months, 12mo: 12 months. For treatment discontinuations outcomes, an OR < 1 favours statin users. For efficacy outcomes, an OR > 1 favours statin users (the efficacy outcome ‘change in depression score’ was transformed from mean difference to odds ratio and depicted as such for the purpose of this illustration)

Adjusted data for the full set analysis of all primary and secondary outcomes are in Fig. 2. Number of events are in Additional file, Table A8; unadjusted and adjusted complete case analysis and full set analysis in Additional file, Tables A9a-c; regression analyses in Additional file, Tables A10a-j—these also include safety data about individual adverse events.

Antidepressant + statin use was associated with lower all-cause discontinuation of antidepressant treatment (i.e. higher acceptability) compared to the antidepressant-only group at all time-points, which remained consistent after adjustment for both the complete case analysis and the full set analysis (*N* 673,177; aOR_2 months_ 0.88, 99% CI 0.85 to 0.91; aOR_6 months_ 0.81, 99% CI 0.79 to 0.84; aOR_12 months_ 0.78, 99% CI 0.75 to 0.81).

Tolerability findings varied depending on the model used. Tolerability (i.e. discontinuation of antidepressant treatment due to adverse events) seemed worse in the antidepressant + statin group at all time-points for both datasets in the unadjusted analyses. However, after adjusting for confounders, this association was not confirmed in the complete case analysis and suggested better tolerability for the antidepressant + statin group at 2 and 6 months in the full set analysis (*N* 673,177; aOR_2 months_ 0.92, 99% CI 0.87 to 0.98; aOR_6 months_ 0.94, 99% CI 0.89 to 0.99; aOR_12 months_ 1.02, 99% CI 0.97 to 1.06).

Though unadjusted analyses showed higher response and remission rates and lower depression scores for the antidepressant + statin group at most time-points in the complete case and full set analyses, adjustment did not confirm these results for any of the efficacy outcomes measured (*N* 673,177; range aOR_2-12 months_ 1.00 to 1.02; range aMD_2-12 months_ − 0.01 to − 0.02).

#### Additional analyses

Considering the age difference between the two study groups, a subgroup analysis including only patients aged > 65 years was conducted (i.e. excluding 588,196 participants with an age during the included episode < 65 years). Characteristics of this subgroup (*N* 59,124 on antidepressant-only, *N* 25,857 on antidepressant + statin) and analyses for all outcomes are in Additional file, Tables A11a-d. Results largely reflected those of the whole study cohort: people on antidepressant + statin showed higher acceptability at all time-points (aOR_2 months_ 0.89, 99% CI 0.85 to 0.93; aOR_6 months_ 0.85, 99% CI 0.81 to 0.89; aOR_12 months_ 0.81, 99% CI 0.78 to 0.88) and higher tolerability at 2 months (aOR_2 months_ 0.92, 99% CI 0.86 to 0.99), with no difference for efficacy outcomes.

The effect estimates of the multiple logistic regression analyses for statins (Additional file, A10) differed from those of aspirin for acceptability and tolerability—details in Additional file, A12a [18, 33–35] and Table A12b.

## Discussion

Our study was based on a large cohort of 673,177 patients with depression on antidepressant monotherapy, of whom 46,482 were concomitantly taking a statin. Patients were followed up for 12 months in real-world conditions. We had access to data about numerous potential confounders and performed unadjusted and adjusted analyses for each outcome. We reported results for both the complete case (sensitivity) and full set (primary) analyses since both carry valuable information [36]. Overall, we found an association between antidepressant + statin, compared to antidepressant-only, and fewer antidepressant treatment discontinuations due to any cause at 2 to 12 months. Similarly, the antidepressant + statin group showed better antidepressant treatment tolerability at 2 months and 6 months on the primary analysis, though this association was not observed on the sensitivity analysis on complete cases only. We did not observe differences for any of the PHQ-9 efficacy outcomes. Results did not materially change in patients aged > 65 years and when compared to those for aspirin users, suggesting that the inverse association between statin use and antidepressant treatment discontinuations was independent from age difference and specific to statins but not to aspirin.

Our findings for acceptability and tolerability are in line with another cohort study that highlighted a modest yet significant association between adherence to SSRIs and adherence to statins (*N* 201 777 l, *B* 0.28, 95% CI 0.28 to 0.29) [37]. Antidepressant adherence is an important concern: a recent trial in primary care patients has linked antidepressant treatment discontinuation to a higher risk of depressive relapse (*N* 478, hazard ratio 2.06, 95% CI 1.56 to 2.70) within 1 year [38]. Consistent with previously reported data [8], we saw a high percentage of antidepressant dropouts in both the exposure and comparison arms at all time-points (Additional file, A8); however, the odds of treatment discontinuation were up to 22% lower in the antidepressant + statin group at 12 months. These patients were also concurrently taking many other non-psychiatric medications and had higher comorbidities (Table 1). A recent cohort study of UK primary care data has found an association between polypharmacy and fewer antidepressant discontinuations in people with comorbid depression and type 2 diabetes (*N* 73 808, hazard ratio 0.45; 95% CI 0.37 to 0.50) [39]—an association explained by the observation that patients on polypharmacological treatment represent individuals with worse physical and mental health who are more likely to benefit and therefore adhere to antidepressant treatment while also interacting with multiple physicians and a larger medical support network. Nevertheless, this conclusion would not explain why we could not see the same effect on a comparable group using aspirin in the sensitivity analysis (Additional file, A12), and the focus on a sample of primary care patients reduces the impact of these confounders. A possible implication is that patients newly diagnosed with depression who are started on antidepressants and are not concurrently taking other drugs, such as statins, may benefit from medication adherence support. It is conceivable that combining statin and antidepressant treatment could improve the acceptability and possibly the tolerability of the latter. However, the mechanisms behind such effect remain unclear and further translational and clinical studies are required to explain the biological and psycho-social bases of this association.

In this study, we could not replicate previous findings suggesting the efficacy of combining statins and antidepressants for the treatment of depression. A meta-analysis of four small randomised controlled trials (*N* 238) had found a moderate improvement at 2 months (SMD − 0.48, 95% CI − 0.74 to − 0.22) in scores of depression in the statin plus antidepressant arm [18]. Also, an observational study had shown that the concomitant use of SSRIs and statins, compared to SSRIs alone, was associated with lower psychiatric hospital contacts due to depressive episodes (*N* 872,216, adjusted hazard ratio 0.64, 95% CI 0.55 to 0.75) [40]. This study is akin to ours in its design, including a large population and correcting for similar potential confounders. However, their sample included all incident SSRI users, whereas we considered patients with a recorded diagnosis of depression and on any antidepressant. Their efficacy outcomes were based on psychiatric hospital contact, which likely gauges more severe psychopathology as compared to our outcomes based on a self-rated PHQ-9 scale. Further, PHQ-9 scores were only available for a minority of our sample (Additional file, A7); therefore, despite the large imputation procedure, our study may not have had enough power to identify any augmenting antidepressant effect of statins. While a Cox proportional-hazard (i.e. time-to-event) methodology was used in that study, we assessed outcomes at fixed timepoints using multiple logistic regression. Finally, they used 95% confidence intervals, while we calculated more conservative 99% confidence intervals. On these bases, our null findings for efficacy should be received with caution. A recently completed clinical trial of simvastatin plus standard of care in treatment-resistant depression has not identified any beneficial effect (MD − 0.61; 95% CI − 3.69 to 2.46) [32]. An ongoing trial examining the effects of statins in addition to antidepressants in comorbid major depressive disorder and obesity [41] may prove informative in this respect.

### Strengths and limitations

The main limitation of our study is its observational design, which restricts the potential to make causal inferences. Statins had not been prescribed as a supplement to antidepressant medications with the purpose of augmenting their effects, but for their primary indication of preventing or treating a range of cardiovascular, cerebrovascular, and metabolic disorders. We divided patients in an exposed and unexposed group depending on statin use at baseline: in other words, statins had not necessarily been initiated at the same time of starting an antidepressant for a depressive episode, and the duration of statin exposure was not known, because in real-world, as compared to a corresponding randomised controlled trial, statins would not be prescribed for treating depression. This can expectedly lead to confounding by indication, which we handled by using a model that controls for numerous potential confounders [25, 26]. Our design still allowed to investigate how co-occurring prescriptions of statins and antidepressants can affect antidepressant adherence and the progression of the depressive episode. Residual confounding, however, remains a possibility [42]. Indication bias and residual confounding can affect the internal validity of any observational study, especially when compared to randomised controlled trials; thus, all our findings require a cautious interpretation. Nevertheless, compared to randomised trials, this study involved a large, more representative population of patients with depression across England, including people with multiple comorbidities, followed up for 1 year. This allowed for a longitudinal evaluation of all outcomes as well as greater generalisability. Although secondary outcome measures were not corrected for multiple comparisons, effect estimates were conservative as we calculated 99% confidence intervals.

Our study did not use a matching strategy, such as propensity-score matching, because this would not allow for multiple imputations, which may itself introduce bias [43]. However, our model—adjusted for several confounding variables via multivariable logistic regression analyses—reduces the risk of bias due to lack of matching, while also permitting the use of multiple imputations more effectively [44, 45].

Another methodological limitation concerns the abovementioned extent of imputed data for the effects on depressive symptoms, because PHQ-9 scores had been recorded more infrequently than other variables. Therefore, our study may have been underpowered to detect the effects of statins on the efficacy outcomes, which could explain differences with prior investigations [18, 40]. Previous cohort studies, especially those based on large nationwide databases, have focused on ‘hard efficacy outcomes’, such as psychiatric hospitalisations [40], possibly better tailored to gauge more severe illness. Our primary care database had the advantage of containing a more granular measure of psychopathology, by means of a scale for depressive symptoms. However, the PHQ-9 remains a self-rated and quite simple instrument, thus perhaps lacking the sensitivity to detect modest but important effects of treatment. Furthermore, we assumed that unavailable data was missing-at-random based on a previous subgroup analysis of the entire dataset [31]; however, the reporting of both the complete case and full set analyses strengthens our confidence in the validity of the imputation procedure.

We acknowledge that our study design did not account for censoring individuals in the control group who initiated statin therapy during the observation period. This could introduce bias and confound the interpretation of our results, as those individuals would experience treatment change that is not captured in the analysis.

We could not use a time-to-event analysis (e.g. Cox proportional-hazard model) because the proportional-hazard assumptions for treatment discontinuations were found to be implausible when checked against the available data, hence the choice of multiple logistic regression at pre-specified timepoints, which does not rely on similar assumptions and allows comparison with prior clinical trials [18]. A time-to-event analysis however could have provided useful information about varying follow-up lengths.

There is a possibility that some of the treatment discontinuations due to any cause would have occurred in patients who have remitted from depressive symptoms. Such occurrence would indeed be possible in both clinical trials (e.g. patients who are lost to follow-up) and observational studies. However, we believe that this would not be a major factor in this study of primary care patients because clinical guidelines followed by GPs advise continuing antidepressant medication for at least 6 months after symptomatic remission [4]. Guidelines for primary care use of antidepressants [4] and statins [46] have evolved over the years, which may have had an influence on prescribing patterns; however, we adjusted for ‘year of diagnosis’ to minimise this possible bias.

Regarding our additional analyses, the comparison between the effects of aspirin and statins was based on the estimates of the primary multiple regression analysis for statins: it is therefore at risk of bias (i.e. ‘table 2 fallacy’) [47] since we did not consider other variables that could potentially confound results for aspirin but not for statins. Also, aspirin is a medication available over-the-counter, unlike several statins, and this might have caused a dilution to null of the findings for this drug.

Finally, we did not analyse for potentially distinct effects between different statins. Some studies have proposed that the beneficial effect of statin in depression might depend on their ability to cross the blood–brain barrier, thus on their lipophilicity [48], though outcomes in this respect have sometimes proved conflicting [49, 50].

## Conclusions

In conclusion, this real-world cohort study found that concomitant antidepressant and statin use in people with depression is associated with lower antidepressant treatment discontinuations, but it is not more efficacious than antidepressant alone. Further studies are needed to clarify these associations. Meanwhile, adherence with antidepressant treatment should be monitored in patients who are not taking concurrent medications such as statins.

### Supplementary Information

**Additional file 1. **

## Data Availability

To guarantee the confidentiality of personal and health information, only the authors have had access to the data during the study in accordance with the relevant license agreements. Access to the QResearch data is according to the information on the QResearch website (https://www.QResearch.org/).
