# Supplementary material for: Real-world outcomes of concomitant antidepressant and statin use in primary care patients with depression: a population-based cohort study
Source: BMC Med. 2023 Nov 7;21:424. doi: 10.1186/s12916-023-03138-5 (PMC10631198; doi:10.1186/s12916-023-03138-5)
Supplement: Supplementary file 1 — Additional file 1. [file 12916_2023_3138_MOESM1_ESM.docx]

# STATaddon-QResearch – Additional file

## A1. Study protocol

Available at <https://osf.io/96zcn/>

*CHANGES TO THE PROTOCOL*

There were no major changes to the original protocol. The only minor change involves the several safety outcomes (i.e., any adverse event, self-harm, suicidality, completed suicide, all-cause mortality), which were analysed and reported in the Supplementary Material as per protocol; however, we did not include these outcomes in our final manuscript due to space constraints.

### ABSTRACT

#### Introduction

Statins have been proposed for repurposing in the treatment of depressive disorders. This study will assess the real-world acceptability, tolerability, safety, and efficacy of statin plus antidepressant compared to antidepressant-only in depression.

#### Methods

This study is part of a larger project exploring antidepressant treatments for depression (QResearch: OX3 “Choosing the right antidepressant for depressive disorder”, 18/EM/0400).

We will conduct a population-based cohort study, using a between-subjects design, on QResearch primary care research database. Participants registered for at least one year from 1^st^ January 1998, aged 18-100 years, diagnosed with a new episode of depression, and on any statin plus antidepressant versus antidepressant-only will be included. Primary outcomes will be acceptability (antidepressant drop-out due to any cause), and tolerability (antidepressant drop-out due to an adverse event). Secondary outcomes will include safety (any adverse event, self-harm, suicidality, completed suicide, all-cause mortality) and efficacy (response, remission, change in depression score on the Patient Health Questionnaire-9). All outcomes will be assessed at two, six, and twelve months from initial antidepressant prescription, using multiple logistic regression with adjustment for several potential confounders, calculating odds ratio/mean differences with 99% confidence intervals.

#### Discussion

Our findings will complement the available evidence on the short- and long-term acceptability, tolerability, safety, and efficacy of statins in addition to antidepressants in people with depression, with possible implications for patients, clinicians, public health, and further research.

#### Funding

Wellcome Trust, National Institute for Health Research

### INTRODUCTION

The clinical and societal burden of depression worldwide is large (1). Antidepressants are the mainstay treatment in adults with moderate to severe depression (2-4), but only about 50% of these patients respond to first-line antidepressants (efficacy), more than 25% discontinue antidepressants due to any cause (acceptability), and around 10% suspend treatment because of adverse events (tolerability) after only two months (5, 6). Any strategy that could improve these outcomes while remaining safe would potentially benefit many people suffering from depressive disorders.

Growing evidence from pre-clinical, translational, and clinical studies suggests that statins (3-hydroxy-3-methylglutaryl-Coenzyme A reductase inhibitors), traditionally used for managing cardiometabolic disorders (7), could be repurposed for the treatment of depression (8). A meta-analysis of randomised controlled trials has shown that statin plus antidepressant, compared to antidepressant plus placebo, is more efficacious in reducing depressive symptoms in patients diagnosed with depressive disorder at two to three months (9). Comparable acceptability, tolerability, and safety between the two study arms were noticed, but the certainty of the evidence for these findings was modest in view of the very low number of events reported over a short follow-up (9).

Clinical trials are indeed best for evaluating treatment efficacy, but small sample sizes, short follow-ups, and highly selected populations can affect their ability to measure treatment discontinuation and adverse events (10), as well as limiting the applicability and generalisability of their findings (11). Conversely, observational studies with robust methodology (12) that include larger populations, are conducted over longer periods, and can involve participants with multiple comorbidities and co-occurring treatments can complement the evidence provided by trials.

Our team is working on an extensive project that aims to optimise antidepressant treatment for depression (13, 14). This project was independently reviewed and approved by the QResearch scientific committee under 18/EM/0400, and registered on QResearch under OX3 “Choosing the right antidepressant for depressive disorder”. As part of this project, the effects of medications other than antidepressants on the treatment of depression is to be assessed. Therefore, following the same methodology of the stem project, the proposed study will explore the real-world acceptability, tolerability, safety, and efficacy of statin plus antidepressant versus antidepressant alone in patients with depressive disorders on QResearch.

### METHODS

The proposed study is part of the larger project on epidemiological data (13) exploring antidepressant treatments for depression (QResearch: OX3 “Choosing the right antidepressant for depressive disorder”, 18/EM/0400).

#### Setting

We will use data from the latest version of QResearch primary care research registry ([www.qresearch.org](http://www.qresearch.org)), the largest general practice research database in the UK. This database contains the anonymised electronic healthcare records of over 35 million patients ever registered with 1,500 UK general practices (15). It uses the Egton Medical Information Systems (EMIS) to obtain consent to provide data, which is the major supplier of primary care computer systems in the UK and is commissioned for most general practices in the country.

Information on QResearch accurately and reliably records patient demographic data (e.g., year of birth, gender, socio-economic status), characteristics (e.g., height, weight, smoking status), symptoms, clinical diagnoses, consultations, referrals, prescribed medications, and results of investigations. The registry has been robustly validated for research on the adverse outcomes of antidepressants in adult and elderly populations (16, 17). Within QResearch, we will use data coming from English general practices.

#### Population

Cohort construction is summarised in Figure 1.

### **Figure 1.** Cohort construction flow chart


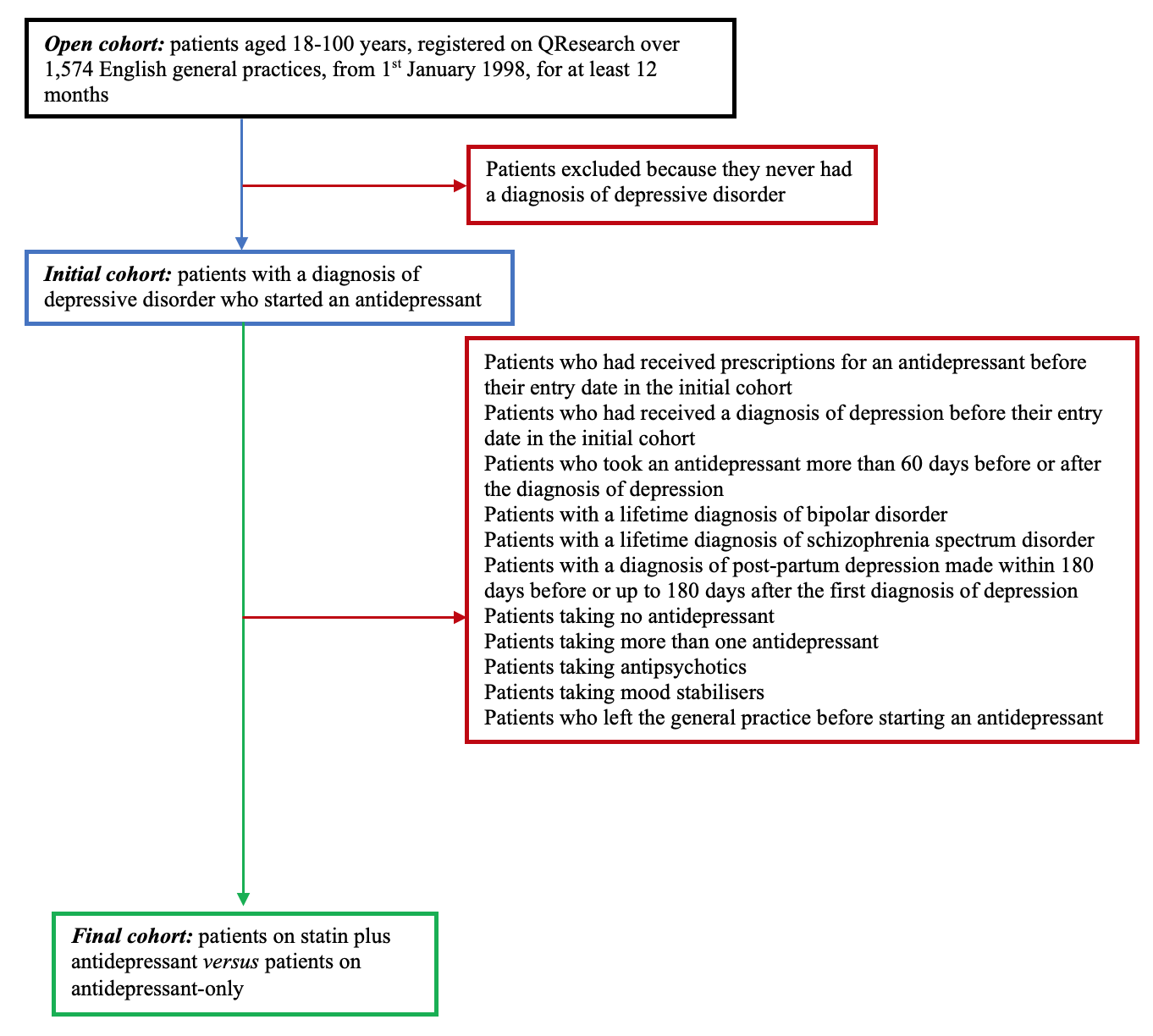


Firstly, we will ascertain an open cohort of patients aged 18-100 years from eligible English practices since 1^st^ Jan 1998. Patients will be included if they have been registered with the practice for at least 12 months. We will use previously validated (16, 17) “Read codes” (i.e., a coded thesaurus of clinical terms used in English primary care) to identify patients with a diagnosis of depressive disorder (Supplementary Material, S1). Initiation of first antidepressant treatment, as further detailed below, will represent the entry date to the initial cohort.

We will exclude: patients with a lifetime diagnosis of bipolar disorder or schizophrenia spectrum disorder; patients prescribed antipsychotics or mood stabilisers; patients with a diagnosis of post-partum depression made within 180 days before or up to 180 days after the first diagnosis of depression; patients prescribed no antidepressant or more than one antidepressant at baseline; patients who had received prescriptions for an antidepressant or had a diagnosis of depression before their cohort entry date; patients with a diagnosis of depression made two months before or two months after starting an antidepressant.

Our final cohort for analysis will include patients with first-episode depression followed-up for 12 months: an exposed group on statin plus antidepressant, and an unexposed group on antidepressant only.

#### Exposure, comparison

The exposure of interest will be the use of any statin at the same time of the prescription of any antidepressant monotherapy (statin plus antidepressant), in comparison to the use of any antidepressant alone (antidepressant-only) – drugs currently licensed for use in the UK according to the British National Formulary (BNF, <https://bnf.nice.org.uk>) (Supplementary Material, S2).

Patients will be classified as continually exposed to an antidepressant during periods where there were no gaps of more than 30 days between the end of a prescription and the beginning of the next, as most prescriptions at the beginning of a treatment are prescribed for no longer than 28 to 30 days. Patients will be divided into the two study arms depending on whether they were taking a statin at baseline.

#### Outcomes

All outcomes will be measured at 2 months, 6 months, and 12 months from the initial prescription of antidepressants.

##### Primary outcomes

- Acceptability will be measured as all-cause antidepressant treatment drop-out rate. Treatment drop-out will consist of: a patient has a >30-day gap between the end of a prescription of an antidepressant and the start of the next prescription (based on an average prescription length of 28 to 30 days); a patient switches to another antidepressant; a patient is prescribed any additional antidepressant, mood stabiliser, or antipsychotic.

- Tolerability will be measured as antidepressant treatment drop-out rate, as defined above, within 30 days from any adverse event (Supplementary Material, S3); therefore, treatment drop-out would have to occur within 30 days of an adverse event to be assumed to be a consequence of the adverse event

##### Secondary outcomes

- Safety outcomes will be measured as rates of: number of patients with at least one adverse event (Supplementary Material, S3); self-harm; suicidality; completed suicide; all-cause mortality.

- Efficacy outcomes will be measured via the Patient Health Questionnaire (PHQ-9) as: response (50% score reduction); remission (score <5); change in depression score.

#### Confounder variables

Confounders will include baseline variables that have been shown to be potential risk factors for the outcomes and are also potentially associated with the likelihood of receiving antidepressant or statin treatment, based on previously validated studies on QResearch (16-18){Coupland, 2011, Antidepressant use and risk of adverse outcomes in older people: population based cohort study;Coupland, 2018, Antidepressant use and risk of adverse outcomes in people aged 20-64 years: cohort study using a primary care database;Vinogradova, 2016, Discontinuation and restarting in patients on statin treatment: prospective open cohort study using a primary care database}. These will include: age at study entry; sex; body mass index (BMI); year of diagnosis of depression; type of diagnosis of depression (major depressive disorders, minor depression, other); severity of index diagnosis of depression (mild, moderate, severe); deprivation status (Townsend deprivation score); smoking status (non-smoker, ex-smoker, light smoker 1-9 cigarettes/day, moderate smoker 10-19 cigarettes/day, heavy smoker ≥20 cigarettes/day, not recorded); alcohol intake (none, trivial <1 unit/day, light 1-2 unit/day, medium 3-6 unit/day, heavy 7-9 unit/day, very heavy >9 unit/day, not recorded); ethnic group (White, African-Caribbean, Asian, other); comorbidities (coronary heart disease, stroke, diabetes, hypertension, cancer, epilepsy/seizures, hypothyroidism, osteoarthritis, rheumatoid arthritis, suicidality, obesity, asthma/chronic obstructive airways disease, osteoporosis, liver disease, renal disease); antidepressant category [selective serotonin reuptake inhibitors (SSRIs), tricyclics (TCAs), monoamine oxidase inhibitors (MAOIs), other antidepressant]; use of other drugs (anticonvulsants, hypnotics/anxiolytics, antihypertensives, aspirin, anticoagulants, non-steroidal anti-inflammatory drugs, bisphosphonates, oral contraceptives, hormone replacement therapy).

#### Handling of missing data

Excluding subjects with missing values would reduce the study sample size, thus decreasing precision and power, and potentially introducing bias (19). We will use multiple imputation by chained equations to impute data when actual values are not available. We will first analyse the patterns of distribution of missing values to assess if the data are suitable for multiple imputation using chained equations. For each imputation we will generate ten imputed datasets and we will combine coefficient estimates across these using Rubin’s rules (20). We will include all the confounding variables in the multiple imputation process, along with the outcome variable as it carries information about predictors’ missing values.

#### Statistical analysis

Stata MP version 16.0 statistical software (21) will be used to conduct all the analyses.

Baseline characteristics will be explored with descriptive statistics for the total sample, as well as divided into a statin plus antidepressant and an antidepressant-only groups.

For all outcomes, we will use intention-to-treat analyses with multiple logistic regressions, clustered by GP practices, to calculate odds ratios (ORs, for dichotomous outcomes) or mean differences (MDs, for continuous outcomes) with 99% confidence intervals (99% CIs), both unadjusted and adjusted for the abovementioned confounders. Results will be separately reported for the complete case and the full set (imputed) analyses.

#### Patient and public involvement

People with lived experience of unipolar depression were recruited from across the UK by the Oxford Precision Psychiatry Lab of the Oxford Health Biomedical Research Centre (<https://oxfordhealthbrc.nihr.ac.uk/our-work/oxppl/patient-involvement-and-ethics/>) and were involved in the design of the overall study (13).

### DISCUSSION

Our protocol describes a population-based cohort study that aims to assess the real-world acceptability, tolerability, safety, and efficacy of statin plus antidepressant versus antidepressant-only treatment in adults with depressive disorders in primary care. This work fits within a more comprehensive project (13, 14) that ultimately seeks to develop and test a precision medicine approach to the pharmacological treatment of depression, by integrating data from both randomised controlled trials and epidemiological studies.

Our study builds on the solid methodologies of previous work done on QResearch in adults with depressive disorders (16, 17). This database thoroughly records detailed information about primary care patients in England (15), which is usually not available in other nationwide databases. This allows for a meticulous accounting of patients’ characteristics and potentially confounding variables, thus strengthening the internal validity of our results.

This work contributes to the area of clinical research in the treatment of depression by focussing on clinically relevant outcomes that have also been evaluated in randomised controlled trials (9), but measured in a real-world setting. Compared to these trials, we will be able to include an in-depth assessment of treatment discontinuations, adverse events, and antidepressant efficacy on a longer follow-up of twelve months. Furthermore, we can involve a larger population of patients with depression including those with multiple comorbidities and co-occurring treatments, normally excluded from clinical trials, thus increasing the external validity of our results.

Our study has some important limitations. The main issue is that, due to its observational nature, this study has lower internal validity compared to randomised controlled trials due to confounding by indication bias and residual confounding. Despite having access to and controlling for a very high number of potentially confounding variables, a large and adequately conducted randomised controlled trials can better estimate causal effects of interventions. Another problem is that we divide patients in an exposed (statin plus antidepressant) and unexposed (antidepressant-only) groups depending on statin use at baseline. In other words, statins are not initiated at the same time of starting an antidepressant for a depressive episode, because in real-world, as compared to a corresponding randomised controlled trial, statins would not be prescribed for treating depression. However, this design still allows to investigate how co-occurring prescriptions of statins and antidepressants can affect antidepressant adherence and the progression of the depressive episode.

Finally, some information before date of entry in the QResearch database may have not been recorded. For example, we will exclude patients if they have received prescriptions for an antidepressant or had a diagnosis of depression before their entry date in the cohort, but these may have not been recorded in all cases.

In summary, our study will add to the existing evidence about the acceptability, tolerability, safety, and efficacy of statin add-on to antidepressant treatment in patients with depressive disorders. Our findings will have the potential to inform further clinical trials and, taken together with data from the latter, can have consequences for patients, clinicians, and public health.

### REFERENCES

1. Demyttenaere K, Van Duppen Z. The Impact of (the Concept of) Treatment-Resistant Depression: An Opinion Review. Int J Neuropsychopharmacol. 2019;22(2):85-92.

2. Association AP. Clinical practice guideline for the treatment of depression across three age cohorts. Washington: American Psychological Association; 2019 [updated February 2019. Available from: <https://www.apa.org/depression-guideline>.

3. Malhi GS, Bell E, Singh AB, Bassett D, Berk M, Boyce P, et al. The 2020 Royal Australian and New Zealand College of Psychiatrists clinical practice guidelines for mood disorders: Major depression summary. Bipolar Disord. 2020;22(8):788-804.

4. Health NCCfM. Depression - The NICE guideline to the treatment and management of depression in adults (updated edition) London: The British Psychological Society & The Royal College of Psychiatrists; 2020 [Available from: <https://www.nice.org.uk/guidance/cg90/evidence/full-guideline-pdf-4840934509>.

5. Rush AJ, Trivedi MH, Wisniewski SR, Nierenberg AA, Stewart JW, Warden D, et al. Acute and longer-term outcomes in depressed outpatients requiring one or several treatment steps: a STAR*D report. Am J Psychiatry. 2006;163(11):1905-17.

6. Cipriani A, Furukawa TA, Salanti G, Chaimani A, Atkinson LZ, Ogawa Y, et al. Comparative efficacy and acceptability of 21 antidepressant drugs for the acute treatment of adults with major depressive disorder: a systematic review and network meta-analysis. Lancet. 2018;391(10128):1357-66.

7. NICE. Surveillance report 2018 – Cardiovascular disease: risk assessment and reduction, including lipid modification (2014) NICE guideline CG181. Surveillance report 2018 – Cardiovascular disease: risk assessment and reduction, including lipid modification (2014) NICE guideline CG181. London: National Institute for Health and Care Excellence (NICE)

Copyright © NICE 2018.; 2018.

8. De Giorgi R, Rizzo Pesci N, Quinton A, De Crescenzo F, Cowen PJ, Harmer CJ. Statins in Depression: An Evidence-Based Overview of Mechanisms and Clinical Studies. Front Psychiatry. 2021;12:702617.

9. De Giorgi R, De Crescenzo F, Rizzo Pesci N, Martens M, Howard W, Cowen PJ, et al. Statins for major depressive disorder: A systematic review and meta-analysis of randomized controlled trials. PLoS One. 2021;16(3):e0249409.

10. Phillips R, Hazell L, Sauzet O, Cornelius V. Analysis and reporting of adverse events in randomised controlled trials: a review. BMJ Open. 2019;9(2):e024537.

11. Kennedy-Martin T, Curtis S, Faries D, Robinson S, Johnston J. A literature review on the representativeness of randomized controlled trial samples and implications for the external validity of trial results. Trials. 2015;16:495.

12. Hernán MA. Methods of Public Health Research - Strengthening Causal Inference from Observational Data. N Engl J Med. 2021;385(15):1345-8.

13. De Crescenzo F, Garriga C, Tomlinson A, Coupland C, Efthimiou O, Fazel S, et al. Real-world effect of antidepressants for depressive disorder in primary care: protocol of a population-based cohort study. Evid Based Ment Health. 2020;23(3):122-6.

14. Tomlinson A, Furukawa TA, Efthimiou O, Salanti G, De Crescenzo F, Singh I, et al. Personalise antidepressant treatment for unipolar depression combining individual choices, risks and big data (PETRUSHKA): rationale and protocol. Evid Based Ment Health. 2020;23(2):52-6.

15. Hippisley-Cox J, Vingradova J, Coupland C, Pringle M. Comparison of key practice characteristics between general practices in England and Wales and general practices in the QRESEARCH database NHS information centre2005 [Available from: <https://www.qresearch.org/Public_Documents/Characteristics%20of%20QRESEARCH%20practices%20_database%20version%208_%20v1.0.pdf>.

16. Coupland C, Dhiman P, Morriss R, Arthur A, Barton G, Hippisley-Cox J. Antidepressant use and risk of adverse outcomes in older people: population based cohort study. Bmj. 2011;343:d4551.

17. Coupland C, Hill T, Morriss R, Moore M, Arthur A, Hippisley-Cox J. Antidepressant use and risk of adverse outcomes in people aged 20-64 years: cohort study using a primary care database. BMC Med. 2018;16(1):36.

18. Vinogradova Y, Coupland C, Brindle P, Hippisley-Cox J. Discontinuation and restarting in patients on statin treatment: prospective open cohort study using a primary care database. Bmj. 2016;353:i3305.

19. Sterne JA, White IR, Carlin JB, Spratt M, Royston P, Kenward MG, et al. Multiple imputation for missing data in epidemiological and clinical research: potential and pitfalls. Bmj. 2009;338:b2393.

20. Rubin DB. Multiple Imputation after 18+ Years. Journal of the American Statistical Association. 1996;91(434):473-89.

21. StataCorp. Stata Statistical Software: Release 17. College Station, TX: StataCorp LLC.; 2021.

### DECLARATION OF INTEREST

RDG is supported by the Wellcome Trust (award 102176/Z/13/Z, grant 216452/Z/19/Z, title “The effects of anti-inflammatory drugs on emotional and reward processing”). FDC is supported by the National Institute for Health Research (NIHR) Research Professorship to Professor Andrea Cipriani (grant RP-2017-08-ST2-006) and by the NIHR Oxford Health Biomedical Research Centre (grant BRC-1215-20005). PJC is supported by the Medical Research Council (MRC) (grant MR/S003037/1, title “Clinical Psychopharmacology of Depression”). CJH is supported by the NIHR Oxford Health Biomedical Research Centre. AC is supported by the National Institute for Health Research (NIHR) Oxford Cognitive Health Clinical Research Facility, by an NIHR Research Professorship (grant RP-2017-08-ST2-006), by the NIHR Oxford and Thames Valley Applied Research Collaboration, and by the NIHR Oxford Health Biomedical Research Centre (grant BRC-1215-20005). The views expressed are those of the authors and not necessarily those of the Wellcome Trust, the NIHR, the MRC, the UK NHS, or the UK Department of Health.

CJH has received consultancy fees from P1vital, Lundbeck, Servier, UCB, Zogenix, J&J, and Syndesi outside of the current work. AC has received research and consultancy fees from INCiPiT (Italian Network for Paediatric Trials), CARIPLO, and Angelini Pharma outside of the current work. The other authors declare that they have no conflict of interest.

### DATA SHARING

To guarantee the confidentiality of personal and health information, only the authors have had access to the data during the study in accordance with the relevant licence agreements. Access to the QResearch data is according to the information on the QResearch website (<https://www.qresearch.org/>).

### ACKNOWLEDGMENTS

The study was funded by the Wellcome Trust (grant 216452/Z/19/Z to Dr De Giorgi) and the National Institute for Health Research (grant RP-2017-08-ST2-006 to Professor Cipriani).

### SUPPLEMENTARY MATERIAL

#### S1. Read Codes for depressive disorders

*Minor depression*

Abnormal depressed feelings

Adjustment disorder with depressed mood

Adjustment reaction, predominant disturbance other emotions C/O - feeling depressed

C/O - feeling unhappy

Depressed mood

Depressive symptoms

Dysphoric mood

Emotional problem

Emotional upset

Loss of capacity for enjoyment

Loss of hope for the future

Low mood

O/E - depressed

PHQ9 score - feeling down or depressed or hopeless

Rebound mood swings

Sad mood

Stress reaction causing mixed disturbance of emotion/conduct Suspected depression

Symptoms of depression

Dysthymia

*Major Depression*

Agitated depression

Anxiety with depression

Arteriosclerotic dementia with depression Atypical depressive disorder

Brief depressive reaction

Brief depressive reaction NOS

Chronic depression

Depressed

Depression

Depression NOS

Depression confirmed

Depressive disorder NEC

Endogenous depression

Endogenous depression - recurrent Endogenous depression first episode Masked depression

Mild depression

Moderate depression

Neurotic depression reactive type Presenile dementia with depression

Prolonged depressive reaction

Psychotic reactive depression

Reactive (neurotic) depression

Recurrent depression

Recurrent major depressive episode

Recurrent major depressive episode NOS

Recurrent major depressive episodes, unspecified

Recurrent major depressive episodes, mild

Recurrent major depressive episodes, moderate

Recurrent major depressive episodes, severe, no psychosis Recurrent major depressive episodes, severe, with psychosis Recurrent major depressive episodes, partial/unspec remission Recurrent major depressive episodes, in full remission

Senile dementia with depression

Severe depression

Single major depressive episode

Single major depressive episode NOS

Single major depressive episode, in full remission Single major depressive episode, mild

Single major depressive episode, moderate

Single major depressive episode, partial or unspec remission Single major depressive episode, severe with psychosis Single major depressive episode, severe without psychosis Single major depressive episode, unspecified

[RFC] Depression

Reactive depression NOS

Antenatal depression

Atypical depression

Depression NOS

Depressive disorder NOS

Depressive episode

Depressive episode, unspecified

Depressive neurosis

Endogenous depression with psychotic symptoms Endogenous depression without psychotic symptoms

Major depression, mild

Major depression, moderately severe

Major depression, recurrent without psychotic symptoms Major depression, severe with psychotic symptoms

Major depression, severe without psychotic symptoms

Mild anxiety depression

Mild depression

Mild depressive episode

Mixed anxiety and depressive disorder

Moderate depressive episode

Monopolar depression NOS

Neurotic depression

Other depressive episodes

Other recurrent depressive disorders

Persistant anxiety depression

Prolonged single episode of reactive depression

Recurr depress disorder cur epi severe without psyc sympt Recurr severe episodes/ major depression+psychotic symptom Recurr severe episodes/psychogenic depressive psychosis Recurrent brief depressive episodes

Recurrent depress disorder cur epi severe with psyc symp Recurrent depressive disorder

Recurrent depressive disorder, current episode mild

Recurrent depressive disorder, current episode moderate Recurrent depressive disorder, unspecified

Recurrent episodes of depressive reaction

Recurrent episodes of psychogenic depression

Recurrent episodes of reactive depression

Recurrent severe episodes of psychotic depression

Recurrent severe episodes/reactive depressive psychosis Severe depressive episode with psychotic symptoms

Severe depressive episode without psychotic symptoms

Single episode agitated depressn w'out psychotic symptoms Single episode major depression w'out psychotic symptoms Single episode of depressive reaction

Single episode of major depression and psychotic symptoms Single episode of psychogenic depression

Single episode of psychogenic depressive psychosis

Single episode of psychotic depression

Single episode of reactive depression

Single episode of reactive depressive psychosis

*Other Read codes for depression*

4 item geriatric depression scale

Acute posttrauma stress state

Acute stress reaction NOS

Assessment using Whooley depression screen Beck depression inventory

Beck depression inventory second edition score Depression - enhanced service completed Depression - enhanced services administration Depression annual review

Depression anxiety stress scales anxiety score Depression anxiety stress scales depression score Depression anxiety stress scales stress score

Depression interim review

Depression management programme

Depression medication review

Depression monitoring administration

Depression monitoring first letter

Depression monitoring second letter

Depression monitoring telephone invite

Depression monitoring third letter

Depression monitoring verbal invite

Depression resolved

Depression screen

Edinburgh postnatal depression scale

Emotional and psychosocial support and advice Geriatric Depression Screen Score

Geriatric depression scale

Geriatric depression scale – 0 point

Geriatric depression scale – 1 point

Geriatric depression scale – 10 points

Geriatric depression scale – 11 points

Geriatric depression scale – 12 points

Geriatric depression scale – 13 points

Geriatric depression scale – 14 points

Geriatric depression scale – 15 points

Geriatric depression scale – 2 points

Geriatric depression scale – 3 points

Geriatric depression scale – 4 points

Geriatric depression scale – 5 points

Geriatric depression scale – 6 points

Geriatric depression scale – 7 points

Geriatric depression scale – 8 points

Geriatric depression scale – 9 points

Geriatric depression scale – <6 points

H/O: depression

HAD scale: depression score

HAMD - Hamilton rating scale for depression

HRSD - Hamilton rating scale for depression

Hospital anxiety and depression scale

MADRS - Montgomery-Asberg depression rating scale Mixed disturbance of conduct and emotion

Mood observations

On depression register

On full dose long term treatment for depression

Other acute stress reaction NOS

Other acute stress reactions

Patient given advice about management of depression Postnatal depression

Postviral depression

Referral for depression self-help video

Referral for guided self-help for depression

Removed from depression register

Stress monitoring 1st letter

[RFC] Postnatal depression

Personal history of affective disorder

Depressive personality disorder

Manic-depress psychosis,depressd, no psychotic symptoms Manic-depress psychosis,depressed type+psychotic symptoms Mood - affective disorders

Postnatal depression NOS

Postpartum depression NOS

Recurrent depressive disorder, current episode mild

Seasonal depressive disorder

#### S2. Statins and antidepressants included

Statins were included based on those currently available in the UK according to the British National Formulary (BNF) (<https://bnf.nice.org.uk>):

- simvastatin

- atorvastatin

- pravastatin

- fluvastatin

- rosuvastatin

Antidepressants were categorised according to four classes as in the BNF:

- selective serotonin reuptake inhibitors (SSRIs): citalopram, escitalopram, fluoxetine, fluvoxamine, paroxetine, sertraline

- tricyclics (TCAs): amitriptyline, amoxapine, butriptyline, clomipramine, desipramine, dosulepine, doxepin, imipramine, iprindole, lofepramine, maprotiline, mianserin, nortriptyline, protriptyline, trimipramine, viloxazine

- monoamine oxidase inhibitors (MAOIs): isocarboaxazid, moclobemide, phenelzine, tranylcypromine

- other antidepressants: agomelatine, duloxetine, mirtazapine, nefazodone, reboxetine, tryptophan, trazodone, venlafaxine, vortioxetine

#### S3. List of adverse events

Nausea, headache, dry mouth, insomnia, dizziness, sedation/ somnolence, diarrhoea, constipation, sexual dysfunction, fatigue, rhinitis/nasopharyngitis, hyperhidrosis, respiratory disorder (infection, cough), anxiety, decreased appetite, increased appetite, tremor, pain, vomiting, abdominal pain/discomfort, dyspepsia, agitation, visual impairment, ejaculation disorder/erectile dysfunction, weight increased, weight decreased, arrhythmia/heart rate disorder, abnormal dreams, infection, blood pressure increased, blood pressure decreased, extrapyramidal dis- orders, suicidal ideation, suicide behaviour or self-harm, hot flush, dysuria, skin disorder, flatulence, urinary dis- orders, injury, yawning, eye disorders, paraesthesia, nervous system symptoms, feeling cold, menstrual disorder, chest pain, disturbance in attention, libido increased, psychiatric symptoms, fall, confusional state, salivary hypersecretion, accidental overdose, cardiovascular symptoms (e.g., angina), sleep disturbance, oedema, aggression, completed suicide, affect lability, fever, euphoric mood, hypersomnia, memory impairment, muscular skeletal problems, serotonin syndrome, withdrawal syndrome, fractures, upper gastrointestinal bleeding, bleeding at any site, epilepsy/seizures

## A2. “Read codes” for depression

“Read codes” are a coded thesaurus of clinical terms used in English primary care.

***Minor depression***

Abnormal depressed feelings

Adjustment disorder with depressed mood

Adjustment reaction, predominant disturbance other emotions C/O - feeling depressed

C/O - feeling unhappy

Depressed mood

Depressive symptoms

Dysphoric mood

Emotional problem

Emotional upset

Loss of capacity for enjoyment

Loss of hope for the future

Low mood

O/E - depressed

PHQ9 score - feeling down or depressed or hopeless

Rebound mood swings

Sad mood

Stress reaction causing mixed disturbance of emotion/conduct Suspected depression

Symptoms of depression

Dysthymia

***Major Depression***

Agitated depression

Anxiety with depression

Arteriosclerotic dementia with depression Atypical depressive disorder

Brief depressive reaction

Brief depressive reaction NOS

Chronic depression

Depressed

Depression

Depression NOS

Depression confirmed

Depressive disorder NEC

Endogenous depression

Endogenous depression - recurrent Endogenous depression first episode Masked depression

Mild depression

Moderate depression

Neurotic depression reactive type Presenile dementia with depression

Prolonged depressive reaction

Psychotic reactive depression

Reactive (neurotic) depression

Recurrent depression

Recurrent major depressive episode

Recurrent major depressive episode NOS

Recurrent major depressive episodes, unspecified

Recurrent major depressive episodes, mild

Recurrent major depressive episodes, moderate

Recurrent major depressive episodes, severe, no psychosis Recurrent major depressive episodes, severe, with psychosis Recurrent major depressive episodes, partial/unspec remission Recurrent major depressive episodes, in full remission

Senile dementia with depression

Severe depression

Single major depressive episode

Single major depressive episode NOS

Single major depressive episode, in full remission Single major depressive episode, mild

Single major depressive episode, moderate

Single major depressive episode, partial or unspec remission Single major depressive episode, severe with psychosis Single major depressive episode, severe without psychosis Single major depressive episode, unspecified

[RFC] Depression

Reactive depression NOS

Antenatal depression

Atypical depression

Depression NOS

Depressive disorder NOS

Depressive episode

Depressive episode, unspecified

Depressive neurosis

Endogenous depression with psychotic symptoms Endogenous depression without psychotic symptoms

Major depression, mild

Major depression, moderately severe

Major depression, recurrent without psychotic symptoms Major depression, severe with psychotic symptoms

Major depression, severe without psychotic symptoms

Mild anxiety depression

Mild depression

Mild depressive episode

Mixed anxiety and depressive disorder

Moderate depressive episode

Monopolar depression NOS

Neurotic depression

Other depressive episodes

Other recurrent depressive disorders

Persistant anxiety depression

Prolonged single episode of reactive depression

Recurr depress disorder cur epi severe without psyc sympt Recurr severe episodes/ major depression+psychotic symptom Recurr severe episodes/psychogenic depressive psychosis Recurrent brief depressive episodes

Recurrent depress disorder cur epi severe with psyc symp Recurrent depressive disorder

Recurrent depressive disorder, current episode mild

Recurrent depressive disorder, current episode moderate Recurrent depressive disorder, unspecified

Recurrent episodes of depressive reaction

Recurrent episodes of psychogenic depression

Recurrent episodes of reactive depression

Recurrent severe episodes of psychotic depression

Recurrent severe episodes/reactive depressive psychosis Severe depressive episode with psychotic symptoms

Severe depressive episode without psychotic symptoms

Single episode agitated depressn w'out psychotic symptoms Single episode major depression w'out psychotic symptoms Single episode of depressive reaction

Single episode of major depression and psychotic symptoms Single episode of psychogenic depression

Single episode of psychogenic depressive psychosis

Single episode of psychotic depression

Single episode of reactive depression

Single episode of reactive depressive psychosis

***Other Read codes for depression***

4 item geriatric depression scale

Acute posttrauma stress state

Acute stress reaction NOS

Assessment using Whooley depression screen Beck depression inventory

Beck depression inventory second edition score Depression - enhanced service completed Depression - enhanced services administration Depression annual review

Depression anxiety stress scales anxiety score Depression anxiety stress scales depression score Depression anxiety stress scales stress score

Depression interim review

Depression management programme

Depression medication review

Depression monitoring administration

Depression monitoring first letter

Depression monitoring second letter

Depression monitoring telephone invite

Depression monitoring third letter

Depression monitoring verbal invite

Depression resolved

Depression screen

Edinburgh postnatal depression scale

Emotional and psychosocial support and advice Geriatric Depression Screen Score

Geriatric depression scale

Geriatric depression scale – 0 point

Geriatric depression scale – 1 point

Geriatric depression scale – 10 points

Geriatric depression scale – 11 points

Geriatric depression scale – 12 points

Geriatric depression scale – 13 points

Geriatric depression scale – 14 points

Geriatric depression scale – 15 points

Geriatric depression scale – 2 points

Geriatric depression scale – 3 points

Geriatric depression scale – 4 points

Geriatric depression scale – 5 points

Geriatric depression scale – 6 points

Geriatric depression scale – 7 points

Geriatric depression scale – 8 points

Geriatric depression scale – 9 points

Geriatric depression scale – <6 points

H/O: depression

HAD scale: depression score

HAMD - Hamilton rating scale for depression

HRSD - Hamilton rating scale for depression

Hospital anxiety and depression scale

MADRS - Montgomery-Asberg depression rating scale Mixed disturbance of conduct and emotion

Mood observations

On depression register

On full dose long term treatment for depression

Other acute stress reaction NOS

Other acute stress reactions

Patient given advice about management of depression Postnatal depression

Postviral depression

Referral for depression self-help video

Referral for guided self-help for depression

Removed from depression register

Stress monitoring 1st letter

[RFC] Postnatal depression

Personal history of affective disorder

Depressive personality disorder

Manic-depress psychosis,depressd, no psychotic symptoms Manic-depress psychosis,depressed type+psychotic symptoms Mood - affective disorders

Postnatal depression NOS

Postpartum depression NOS

Recurrent depressive disorder, current episode mild

Seasonal depressive disorder

## A3. Inclusion/exclusion criteria

*Inclusion*

All of:

- Patients registered on QResearch between 1^st^ January 1998 and 15^th^ August 2020 for at least 12 months

- Patients aged 18-100 years

- Patients with a new diagnosis of depression (see exclusion criteria below) according to “Read codes”, commencing a new antidepressant (see exclusion criteria below)

*Exclusion*

Any of:

- Patients with a lifetime diagnosis of bipolar disorder or schizophrenia spectrum disorder;

- Patients prescribed antipsychotics or mood stabilisers;

- Patients with a diagnosis of post-partum depression made within 180 days before or up to 180 days after the first diagnosis of depression;

- Patients prescribed no antidepressant or more than one antidepressant at baseline;

- Patients who had received prescriptions for an antidepressant or had a diagnosis of depression before their cohort entry date;

- Patients with a diagnosis of depression made two months before or two months after starting an antidepressant.

## A4. Statins and antidepressants included

Statins were included based on those currently available in the UK according to the British National Formulary (BNF) (<https://bnf.nice.org.uk>):

- simvastatin

- atorvastatin

- pravastatin

- fluvastatin

- rosuvastatin

Antidepressants were categorised according to four classes as in the BNF:

- selective serotonin reuptake inhibitors (SSRIs): citalopram, escitalopram, fluoxetine, fluvoxamine, paroxetine, sertraline

- tricyclics (TCAs): amitriptyline, amoxapine, butriptyline, clomipramine, desipramine, dosulepine, doxepin, imipramine, iprindole, lofepramine, maprotiline, mianserin, nortriptyline, protriptyline, trimipramine, viloxazine

- monoamine oxidase inhibitors (MAOIs): isocarboaxazid, moclobemide, phenelzine, tranylcypromine

- other antidepressants: agomelatine, duloxetine, mirtazapine, nefazodone, reboxetine, tryptophan, trazodone, venlafaxine, vortioxetine

## A5. List of adverse events

Nausea, headache, dry mouth, insomnia, dizziness, sedation/ somnolence, diarrhoea, constipation, sexual dysfunction, fatigue, rhinitis/nasopharyngitis, hyperhidrosis, respiratory disorder (infection, cough), anxiety, decreased appetite, increased appetite, tremor, pain, vomiting, abdominal pain/discomfort, dyspepsia, agitation, visual impairment, ejaculation disorder/erectile dysfunction, weight increased, weight decreased, arrhythmia/heart rate disorder, abnormal dreams, infection, blood pressure increased, blood pressure decreased, extrapyramidal dis- orders, suicidal ideation, suicide behaviour or self-harm, hot flush, dysuria, skin disorder, flatulence, urinary dis- orders, injury, yawning, eye disorders, paraesthesia, nervous system symptoms, feeling cold, menstrual disorder, chest pain, disturbance in attention, libido increased, psychiatric symptoms, fall, confusional state, salivary hypersecretion, accidental overdose, cardiovascular symptoms (e.g., angina), sleep disturbance, oedema, aggression, completed suicide, affect lability, fever, euphoric mood, hypersomnia, memory impairment, muscular skeletal problems, serotonin syndrome, withdrawal syndrome, fractures, upper gastrointestinal bleeding, bleeding at any site, epilepsy/seizures.

## A6. List of confounding variables

Age at study entry; sex; body mass index (BMI); year of diagnosis of depression; type of diagnosis of depression (major depressive disorders, minor depression, other); severity of index diagnosis of depression (mild, moderate, severe); deprivation status (Townsend deprivation score); smoking status (non-smoker, ex-smoker, light smoker 1-9 cigarettes/day, moderate smoker 10-19 cigarettes/day, heavy smoker ≥20 cigarettes/day, not recorded); alcohol intake (none, trivial <1 unit/day, light 1-2 unit/day, medium 3-6 unit/day, heavy 7-9 unit/day, very heavy >9 unit/day, not recorded); ethnic group (white, African-Caribbean, Asian, other); comorbidities (coronary heart disease, stroke, diabetes, hypertension, cancer, epilepsy/seizures, hypothyroidism, osteoarthritis, rheumatoid arthritis, suicidality, obesity, asthma/chronic obstructive airways disease, osteoporosis, liver disease, renal disease); antidepressant category [selective serotonin reuptake inhibitors (SSRIs), tricyclics (TCAs), monoamine oxidase inhibitors (MAOIs), other antidepressant – see Supplementary Material, S3]; use of other drugs (anticonvulsants, hypnotics/ anxiolytics, antihypertensive drugs, aspirin, anticoagulants, non-steroidal anti-inflammatory drugs, bisphosphonates, oral contraceptives, hormone replacement therapy).

## A7. Missing data

### **Table A7.** Missing data (percentage) imputed for the full set analysis

| **Characteristic** | **Missing (%)** | **Complete** |
| --- | --- | --- |
| PHQ-9 |  |  |
| at baseline | 528,830 (78.56) | 673,177 |
| at 2 months | 600,720 (89.24) | 673,177 |
| at 6 months | 654,694 (97.25) | 673,177 |
| at 12 months | 661,413 (98.25) | 673,177 |
| Body Mass Index | 149,057 (22.14) | 673,177 |
| Smoking | 52,880 (7.86) | 673,177 |
| Alcohol | 333,060 (49.48) | 673,177 |
| Ethnic group | 179,854 (26.72) | 673,177 |
| Townsend deprivation score | 2,289 (0.34) | 673,177 |

## A8. Number of events for all outcomes

### **Table A8.** Number of events (percentage) for all outcomes at 2 months, 6 months, and 12 months

|  | **Antidepressant-only** | | **Antidepressant+statin** | |
| --- | --- | --- | --- | --- |
| Outcome | *N^o^ events* | *N^o^ patients* | *N^o^ events* | *N^o^ patients* |
| **Acceptability** |  | |  | |
| 2mo | 242,951 (38.79) | 626,335 | 16,784 (35.83) | 46,842 |
| 6mo | 449,473 (71.76) | 626,335 | 28,988 (61.88) | 46,842 |
| 12mo | 554,512 (88.53) | 626,335 | 36,141 (77.16) | 46,842 |
| **Tolerability** |  | |  | |
| 2mo | 34,162 (5.45) | 626,335 | 3,598 (7.68) | 46,842 |
| 6mo | 54,534 (8.71) | 626,335 | 5,610 (11.98) | 46,842 |
| 12mo | 75,242 (12.01) | 626,335 | 8,614 (18.39) | 46,842 |
| **Any adverse event** |  | |  | |
| 2mo | 282,246 (45.06) | 626,335 | 24,409 (52.11) | 46,842 |
| 6mo | 376,136 (60.05) | 626,335 | 32,490 (69.36) | 46,842 |
| 12mo | 441,264 (70.45) | 626,335 | 37,775 (80.64) | 46,842 |
| **Self-harm** |  | |  | |
| 2mo | 2,154 (0.34) | 626,335 | 53 (0.11) | 46,842 |
| 6mo | 3,878 (0.62) | 626,335 | 88 (0.19) | 46,842 |
| 12mo | 5,201 (0.83) | 626,335 | 122 (0.26) | 46,842 |
| **Suicidality** |  | |  | |
| 2mo | 6,372 (1.02) | 626,335 | 373 (0.80) | 46,842 |
| 6mo | 9,559 (1.53) | 626,335 | 534 (1.14) | 46,842 |
| 12mo | 12,166 (1.94) | 626,335 | 652 (1.39) | 46,842 |
| **Completed suicide** |  | |  | |
| 2mo | 190 (0.03) | 626,335 | 7 (0.01) | 46,842 |
| 6mo | 318 (0.05) | 626,335 | 10 (0.02) | 46,842 |
| 12mo | 443 (0.07) | 626,335 | 16 (0.03) | 46,842 |
| **All-cause mortality** |  | |  | |
| 2mo | 3,203 (0.51) | 626,335 | 879 (1.88) | 46,842 |
| 6mo | 3,203 (0.51) | 626,335 | 879 (1.88) | 46,842 |
| 12mo | 10,797 (1.72) | 626,335 | 3,375 (7.21) | 46,842 |
| **Response** |  | |  | |
| 2mo | 15,701 (35.54) | 44,175 | 1,330 (37.52) | 3,545 |
| 6mo | 3,407 (34.53) | 9,868 | 264 (37.08) | 712 |
| 12mo | 1,812 (30.84) | 5,876 | 148 (35.84) | 413 |
| **Remission** |  | |  | |
| 2mo | 9,031 (13.46) | 67,097 | 872 (16.27) | 5,360 |
| 6mo | 2,767 (16.06) | 17,227 | 273 (21.74) | 1,256 |
| 12mo | 1,506 (13.75) | 10,954 | 157 (19.38) | 810 |

## A9. Unadjusted and adjusted complete case analysis and full set analysis for all outcomes

### **Table A9a.** Unadjusted and adjusted odds ratios (any statin + antidepressant vs antidepressant-only) for acceptability and tolerability at 2 months, 6 months, and 12 months; complete case analysis and full set analysis.

|  |  | **COMPLETE CASE ANALYSIS** | | | | | |
| --- | --- | --- | --- | --- | --- | --- | --- |
|  |  | Unadjusted analysis  N= 673,177 | | | Adjusted analysis  N= 63,801 | | |
| Outcome | Time  point | *OR* | *99% CI* | *P* | *aOR* | *99% CI* | *P* |
| **Acceptability** | 2mo | 0.88 | 0.86 to 0.91 | <0.001 | 0.84 | 0.76 to 0.94 | <0.001 |
|  | 6mo  12mo | 0.64  0.44 | 0.62 to 0.66  0.42 to 0.45 | <0.001  <0.001 | 0.86  0.79 | 0.78 to 0.95  0.70 to 0.88 | <0.001  <0.001 |
| **Tolerability** | 2mo | 1.44 | 1.37 to 1.51 | <0.001 | 1.02 | 0.83 to 1.26 | 0.795 |
|  | 6mo  12mo | 1.43  1.65 | 1.37 to 1.48  1.60 to 1.71 | <0.001  <0.001 | 1.07  1.11 | 0.90 to 1.26  0.97 to 1.28 | 0.316  0.052 |

|  |  | **FULL SET ANALYSIS** | | | | | | |
| --- | --- | --- | --- | --- | --- | --- | --- | --- |
|  |  | Unadjusted analysis  N= 673,177 | | | Adjusted analysis  N= 673,177 | | | |
| Outcome | Time  point | *OR* | *99% CI* | *P* | *aOR* | *99% CI* | *P* | |
| **Acceptability** | 2mo | 0.88 | 0.86 to 0.91 | <0.001 | 0.88 | 0.85 to 0.91 | <0.001 | |
|  | 6mo  12mo | 0.64  0.44 | 0.62 to 0.66  0.42 to 0.45 | <0.001  <0.001 | 0.81  0.78 | 0.79 to 0.84  0.75 to 0.81 | <0.001  <0.001 |  |
| **Tolerability** | 2mo | 1.44 | 1.37 to 1.51 | <0.001 | 0.92 | 0.87 to 0.98 | 0.001 | |
|  | 6mo  12mo | 1.43  1.65 | 1.37 to 1.48  1.60 to 1.71 | <0.001  <0.001 | 0.94  1.02 | 0.89 to 0.99  0.97 to 1.06 | 0.001  0.351 | |

### **Table A9b.** Unadjusted and adjusted odds ratios (any statin + antidepressant vs antidepressant-only) for safety (any adverse event, self-harm, suicidality, completed suicide, and all-cause mortality) at 2 months, 6 months, and 12 months; complete case analysis and full set analysis.

|  |  | **COMPLETE CASE ANALYSIS** | | | | | |
| --- | --- | --- | --- | --- | --- | --- | --- |
|  |  | Unadjusted analysis  N= 673,177 | | | Adjusted analysis  N= 63,801 | | |
| Outcome | Time  point | *OR* | *99% CI* | *P* | *aOR* | *99% CI* | *P* |
| **Any adverse event** | 2mo | 1.33 | 1.28 to 1.37 | <0.001 | 0.94 | 0.85 to 1.06 | 0.140 |
|  | 6mo  12mo | 1.51  1.75 | 1.45 to 1.56  1.68 to 1.82 | <0.001  <0.001 | 0.98  1.00 | 0.88 to 1.09  0.89 to 1.12 | 0.605  0.961 |
| **Self-harm** | 2mo | 0.33 | 0.23 to 0.48 | <0.001 | 0.14 | 0.01 to 1.46 | 0.031 |
|  | 6mo  12mo | 0.30  0.31 | 0.23 to 0.40  0.24 to 0.40 | <0.001  <0.001 | 0.22  0.20 | 0.04 to 1.16  0.06 to 0.72 | 0.019  0.001 |
| **Suicidality** | 2mo | 0.78 | 0.65 to 0.93 | <0.001 | 1.07 | 0.70 to 1.64 | 0.684 |
|  | 6mo  12mo | 0.74  0.71 | 0.64 to 0.87  0.62 to 0.82 | <0.001  <0.001 | 0.94  0.97 | 0.63 to 1.40  0.66 to 1.43 | 0.693  0.840 |
| **Completed suicide** | 2mo | 0.49 | 0.18 to 1.32 | 0.064 | 0.81 | 0.01 to 81.94 | 0.908 |
|  | 6mo  12mo | 0.42  0.48 | 0.18 to 0.95  0.25 to 0.92 | 0.006  0.004 | 0.36  0.32 | 0.01 to 13.65  0.01 to 10.19 | 0.473  0.394 |
| **All-cause mortality** | 2mo | 3.72 | 3.35 to 4.14 | <0.001 | 0.66 | 0.36 to 1.19 | 0.066 |
|  | 6mo  12mo | 3.72  4.43 | 3.35 to 4.14  4.13 to 4.74 | <0.001  <0.001 | 0.66  0.69 | 0.36 to 1.19  0.52 to 0.92 | 0.066  0.001 |

|  |  | **FULL SET ANALYSIS** | | | | | |
| --- | --- | --- | --- | --- | --- | --- | --- |
|  |  | Unadjusted analysis  N= 673,177 | | | Adjusted analysis  N= 673,177 | | |
| Outcome | Time  point | *OR* | *99% CI* | *P* | *aOR* | *99% CI* | *P* |
| **Any adverse event** | 2mo | 1.33 | 1.28 to 1.37 | <0.001 | 0.96 | 0.92 to 0.99 | 0.001 |
|  | 6mo  12mo | 1.51  1.75 | 1.45 to 1.56  1.68 to 1.82 | <0.001  <0.001 | 0.98  0.99 | 0.95 to 1.02  0.95 to 1.03 | 0.174  0.468 |
| **Self-harm** | 2mo | 0.33 | 0.23 to 0.48 | <0.001 | 0.70 | 0.44 to 1.12 | 0.049 |
|  | 6mo  12mo | 0.30  0.31 | 0.23 to 0.40  0.24 to 0.40 | <0.001  <0.001 | 0.73  0.81 | 0.51 to 1.05  0.60 to 1.09 | 0.026  0.068 |
| **Suicidality** | 2mo | 0.78 | 0.65 to 0.93 | <0.001 | 1.08 | 0.89 to 1.31 | 0.298 |
|  | 6mo  12mo | 0.74  0.71 | 0.64 to 0.87  0.62 to 0.82 | <0.001  <0.001 | 1.07  1.04 | 0.91 to 1.27  0.89 to 1.20 | 0.272  0.550 |
| **Completed suicide** | 2mo | 0.49 | 0.18 to 1.32 | 0.064 | 0.98 | 0.24 to 3.98 | 0.977 |
|  | 6mo  12mo | 0.42  0.48 | 0.18 to 0.95  0.25 to 0.92 | 0.006  0.004 | 0.81  1.09 | 0.28 to 2.36  0.48 to 2.47 | 0.609  0.794 |
| **All-cause mortality** | 2mo | 3.72 | 3.35 to 4.14 | <0.001 | 0.69 | 0.62 to 0.78 | <0.001 |
|  | 6mo  12mo | 3.72  4.43 | 3.35 to 4.14  4.13 to 4.74 | <0.001  <0.001 | 0.69  0.72 | 0.62 to 0.78  0.67 to 0.77 | <0.001  <0.001 |

### **Table A9c.** Unadjusted and adjusted odds ratios/mean differences (any statin + antidepressant vs antidepressant-only) for efficacy (response, remission, change in depressive score), as measured on the PHQ-9 at 2 months, 6 months, and 12 months; complete case analysis and full set analysis.

|  |  | **COMPLETE CASE ANALYSIS** | | | | | | | |
| --- | --- | --- | --- | --- | --- | --- | --- | --- | --- |
|  |  | Unadjusted analysis  N= see below | | | | Adjusted analysis  N= see below | | | |
| Outcome | Time  point | *N* | *OR* | *99% CI* | *P* | *N* | *aOR* | *99% CI* | *P* |
| **Response** | 2mo | 47,720 | 1.09 | 0.99 to 1.19 | 0.017 | 22,804 | 1.10 | 0.93 to 1.29 | 0.155 |
|  | 6mo  12mo | 10.580  6,289 | 1.12  1.25 | 0.91 to 1.37  0.93 to 1.70 | 0.160  0.054 | 5,035  2,938 | 1.06  1.47 | 0.74 to 1.52  0.87 to 2.49 | 0.685  0.057 |
| **Remission** | 2mo | 47,720 | 1.25 | 1.13 to 1.38 | <0.001 | 22,804 | 1.16 | 0.95 to 1.41 | 0.052 |
|  | 6mo  12mo | 10.580  6,289 | 1.45  1.51 | 1.20 to 1.75  1.19 to 1.91 | <0.001  <0.001 | 5,035  2,938 | 0.99  1.37 | 0.63 to 1.54  0.73 to 2.55 | 0.938  0.199 |
|  |  | *N* | *MD* | *99% CI* | *P* | *N* | *MD* | *99% CI* | *P* |
| **Change** | 2mo | 72,457 | 0.10 | -0.18 to 0.38 | 0.349 | 22,804 | -0.11 | -0.54 to 0.32 | 0.509 |
| **in depression**  **score** | 6mo  12mo | 18,483  11,764 | 0.01  -0.03 | -0.66 to 0.67  -1.05 to 0.98 | 0.972  0.932 | 5,025  2,935 | -0.02  -1.31 | -1.10 to 1.06  -2.92 to 0.30 | 0.962  0.035 |

|  |  | **FULL SET ANALYSIS** | | | | | |
| --- | --- | --- | --- | --- | --- | --- | --- |
|  |  | Unadjusted analysis  N= 673,177 | | | Adjusted analysis  N= 673,177 | | |
| Outcome | Time  point | *OR* | *99% CI* | *P* | *aOR* | *99% CI* | *P* |
| **Response** | 2mo | 1.08 | 1.02 to 1.14 | <0.001 | 1.01 | 0.94 to 1.09 | 0.715 |
|  | 6mo  12mo | 1.06  1.05 | 1.01 to 1.12  0.99 to 1.11 | <0.001  0.018 | 1.01  1.00 | 0.95 to 1.06  0.96 to 1.04 | 0.723  0.992 |
| **Remission** | 2mo | 1.13 | 1.06 to 1.19 | <0.001 | 1.02 | 0.95 to 1.08 | 0.502 |
|  | 6mo  12mo | 1.10  1.09 | 1.04 to 1.17  1.01 to 1.17 | <0.001  <0.001 | 1.01  1.00 | 0.95 to 1.08  0.92 to 1.08 | 0.541  0.973 |
|  |  | *MD* | *99% CI* | *P* | *MD* | *99% CI* | *P* |
| **Change** | 2mo | -0.08 | -0.23 to 0.07 | 0.136 | -0.01 | -0.16 to 0.14 | 0.846 |
| **in depression**  **score** | 6mo  12mo | -0.05  -0.03 | -0.26 to 0.15  -0.24 to 0.18 | 0.462  0.683 | -0.02  -0.01 | -0.20 to 0.16  -0.12 to 0.11 | 0.748  0.855 |

## A10. Regression analyses for all outcomes

### **Table A10a.** Regression analyses for acceptability at 2 months, 6 months, and 12 months; complete case analysis (CCA) and full set analysis (FSA)

|  | **Acceptability *(CCA, N= 63,801)*** | | | | | | | | | | | |
| --- | --- | --- | --- | --- | --- | --- | --- | --- | --- | --- | --- | --- |
|  | **2 months** | | | | **6 months** | | | | **12 months** | | | |
|  | *OR* | *99% CI* | | *P* | *OR* | *99% CI* | | *P* | *OR* | *99% CI* | | *P* |
| ***Statins*** | 0.84 | 0.76 | 0.94 | <0.001 | 0.86 | 0.78 | 0.95 | <0.001 | 0.79 | 0.70 | 0.88 | <0.001 |
| Antidepressant category (SSRIs) |  |  |  |  |  |  |  |  |  |  |  |  |
| TCAs | 2.60 | 2.22 | 3.03 | <0.001 | 3.13 | 2.57 | 3.82 | <0.001 | 3.02 | 2.29 | 3.99 | <0.001 |
| MAOIs |  |  |  |  |  |  |  |  |  |  |  |  |
| Other antidepressants | 1.60 | 1.43 | 1.79 | <0.001 | 1.71 | 1.51 | 1.93 | <0.001 | 1.62 | 1.36 | 1.92 | <0.001 |
|  |  |  |  |  |  |  |  |  |  |  |  |  |
| PHQ-9 baseline | 1.00 | 0.99 | 1.00 | 0.680 | 1.01 | 1.00 | 1.01 | <0.001 | 1.01 | 1.01 | 1.02 | <0.001 |
| BMI | 0.99 | 0.98 | 0.99 | <0.001 | 0.99 | 0.99 | 0.99 | <0.001 | 0.98 | 0.98 | 0.99 | <0.001 |
|  |  |  |  |  |  |  |  |  |  |  |  |  |
| Read codes for depression (Major depression) |  |  |  |  |  |  |  |  |  |  |  |  |
| Minor depression | 1.29 | 1.22 | 1.38 | <0.001 | 1.26 | 1.19 | 1.34 | <0.001 | 1.20 | 1.12 | 1.30 | <0.001 |
| Other | 1.38 | 1.18 | 1.62 | <0.001 | 1.26 | 1.06 | 1.50 | 0.001 | 1.16 | 0.92 | 1.47 | 0.090 |
|  |  |  |  |  |  |  |  |  |  |  |  |  |
| Townsend deprivation score in fifths (1, least deprived) |  |  |  |  |  |  |  |  |  |  |  |  |
| 2 | 1.07 | 1.00 | 1.14 | 0.012 | 1.09 | 1.02 | 1.16 | <0.001 | 1.04 | 0.96 | 1.13 | 0.210 |
| 3 | 1.12 | 1.04 | 1.20 | <0.001 | 1.20 | 1.12 | 1.29 | <0.001 | 1.16 | 1.06 | 1.27 | <0.001 |
| 4 | 1.18 | 1.09 | 1.27 | <0.001 | 1.29 | 1.19 | 1.39 | <0.001 | 1.23 | 1.12 | 1.36 | <0.001 |
| 5, most deprived | 1.29 | 1.18 | 1.42 | <0.001 | 1.40 | 1.26 | 1.57 | <0.001 | 1.34 | 1.17 | 1.52 | <0.001 |
|  |  |  |  |  |  |  |  |  |  |  |  |  |
| Region of England (East Midlands) |  |  |  |  |  |  |  |  |  |  |  |  |
| East of England | 1.06 | 0.76 | 1.49 | 0.629 | 0.91 | 0.62 | 1.35 | 0.556 | 0.95 | 0.63 | 1.42 | 0.732 |
| London | 0.97 | 0.71 | 1.33 | 0.816 | 0.92 | 0.64 | 1.32 | 0.531 | 0.92 | 0.65 | 1.32 | 0.571 |
| North East | 1.08 | 0.75 | 1.55 | 0.602 | 0.98 | 0.66 | 1.44 | 0.873 | 0.90 | 0.59 | 1.36 | 0.505 |
| North West | 1.11 | 0.82 | 1.50 | 0.385 | 0.97 | 0.68 | 1.38 | 0.821 | 0.88 | 0.63 | 1.24 | 0.343 |
| South Central | 1.05 | 0.78 | 1.42 | 0.647 | 0.96 | 0.67 | 1.36 | 0.756 | 0.92 | 0.65 | 1.30 | 0.540 |
| South East | 1.04 | 0.77 | 1.41 | 0.718 | 0.99 | 0.69 | 1.41 | 0.939 | 0.94 | 0.65 | 1.36 | 0.679 |
| South West | 0.96 | 0.71 | 1.31 | 0.763 | 0.92 | 0.65 | 1.31 | 0.560 | 0.93 | 0.65 | 1.31 | 0.575 |
| West Midlands | 1.14 | 0.84 | 1.55 | 0.257 | 1.04 | 0.73 | 1.47 | 0.793 | 0.96 | 0.68 | 1.36 | 0.777 |
| Yorkshire & Humber | 0.95 | 0.67 | 1.33 | 0.684 | 0.85 | 0.59 | 1.22 | 0.253 | 0.82 | 0.56 | 1.20 | 0.174 |
|  |  |  |  |  |  |  |  |  |  |  |  |  |
| Smoking status (Non-smoker) |  |  |  |  |  |  |  |  |  |  |  |  |
| Ex-smoker | 0.95 | 0.90 | 1.00 | 0.016 | 0.93 | 0.88 | 0.99 | 0.001 | 0.91 | 0.85 | 0.97 | <0.001 |
| Light-smoker | 1.20 | 1.13 | 1.27 | <0.001 | 1.41 | 1.32 | 1.50 | <0.001 | 1.34 | 1.24 | 1.46 | <0.001 |
| Moderate-smoker | 1.17 | 0.99 | 1.39 | 0.014 | 1.46 | 1.20 | 1.78 | <0.001 | 1.31 | 1.01 | 1.71 | 0.008 |
| Heavy-smoker | 1.17 | 0.89 | 1.54 | 0.148 | 1.43 | 1.06 | 1.93 | 0.002 | 1.34 | 0.93 | 1.94 | 0.042 |
|  |  |  |  |  |  |  |  |  |  |  |  |  |
| Alcohol use (Non-drinker/trivial) |  |  |  |  |  |  |  |  |  |  |  |  |
| Light | 0.96 | 0.91 | 1.01 | 0.020 | 0.95 | 0.90 | 1.00 | 0.007 | 0.96 | 0.90 | 1.03 | 0.156 |
| Medium | 0.97 | 0.89 | 1.05 | 0.302 | 1.00 | 0.91 | 1.09 | 0.965 | 0.97 | 0.87 | 1.09 | 0.522 |
| Heavy | 1.14 | 0.95 | 1.38 | 0.064 | 1.12 | 0.91 | 1.37 | 0.168 | 1.04 | 0.81 | 1.34 | 0.671 |
| Very heavy | 1.02 | 0.86 | 1.21 | 0.770 | 1.29 | 1.07 | 1.56 | <0.001 | 1.30 | 1.00 | 1.69 | 0.011 |
|  |  |  |  |  |  |  |  |  |  |  |  |  |
| Ethnic group (White) |  |  |  |  |  |  |  |  |  |  |  |  |
| African/Caribbean | 1.62 | 1.38 | 1.89 | <0.001 | 2.33 | 1.92 | 2.83 | <0.001 | 2.97 | 2.18 | 4.03 | <0.001 |
| Asian | 1.79 | 1.56 | 2.06 | <0.001 | 2.12 | 1.80 | 2.49 | <0.001 | 1.92 | 1.53 | 2.42 | <0.001 |
| Other | 1.57 | 1.37 | 1.82 | <0.001 | 1.76 | 1.51 | 2.05 | <0.001 | 1.81 | 1.41 | 2.31 | <0.001 |
|  |  |  |  |  |  |  |  |  |  |  |  |  |
| Age | 1.00 | 1.00 | 1.00 | 0.985 | 0.99 | 0.99 | 0.99 | <0.001 | 0.98 | 0.98 | 0.99 | <0.001 |
|  |  |  |  |  |  |  |  |  |  |  |  |  |
| Sex (Female) |  |  |  |  |  |  |  |  |  |  |  |  |
| Male | 1.00 | 0.95 | 1.05 | 0.832 | 1.17 | 1.12 | 1.23 | <0.001 | 1.16 | 1.09 | 1.24 | <0.001 |
|  |  |  |  |  |  |  |  |  |  |  |  |  |
| Year of diagnosis (1998-2005) |  |  |  |  |  |  |  |  |  |  |  |  |
| 2006 to 2010 | 0.74 | 0.53 | 1.03 | 0.018 | 0.80 | 0.54 | 1.19 | 0.151 | 0.71 | 0.41 | 1.24 | 0.115 |
| 2011 to 2015 | 0.68 | 0.49 | 0.95 | 0.003 | 0.71 | 0.48 | 1.06 | 0.029 | 0.57 | 0.33 | 0.99 | 0.009 |
| 2016 to 2020 | 0.71 | 0.51 | 0.99 | 0.009 | 0.76 | 0.51 | 1.14 | 0.079 | 0.64 | 0.37 | 1.13 | 0.042 |
|  |  |  |  |  |  |  |  |  |  |  |  |  |
| Coronary heart disease | 1.21 | 1.04 | 1.39 | 0.001 | 1.27 | 1.10 | 1.48 | <0.001 | 1.34 | 1.12 | 1.60 | <0.001 |
| Stroke | 1.00 | 0.84 | 1.19 | 0.973 | 1.22 | 1.01 | 1.46 | 0.006 | 1.05 | 0.86 | 1.28 | 0.533 |
| Diabetes | 1.05 | 0.94 | 1.18 | 0.235 | 1.00 | 0.90 | 1.11 | 0.944 | 0.98 | 0.87 | 1.10 | 0.645 |
| Epilepsy | 0.95 | 0.77 | 1.18 | 0.551 | 1.02 | 0.83 | 1.27 | 0.783 | 0.88 | 0.67 | 1.15 | 0.207 |
| Hypothyroidism | 0.96 | 0.84 | 1.09 | 0.403 | 0.89 | 0.78 | 1.00 | 0.013 | 0.93 | 0.80 | 1.08 | 0.185 |
| Arthritis | 1.02 | 0.93 | 1.12 | 0.595 | 1.12 | 1.02 | 1.24 | 0.002 | 1.11 | 0.99 | 1.24 | 0.020 |
| Anxiety | 1.01 | 0.94 | 1.07 | 0.834 | 0.90 | 0.85 | 0.96 | <0.001 | 0.86 | 0.79 | 0.94 | <0.001 |
| Migraine | 1.03 | 0.94 | 1.12 | 0.420 | 0.98 | 0.90 | 1.07 | 0.614 | 0.93 | 0.83 | 1.04 | 0.076 |
| Cancer | 1.09 | 1.00 | 1.19 | 0.008 | 1.05 | 0.97 | 1.14 | 0.117 | 1.10 | 0.99 | 1.22 | 0.023 |
| Asthma | 1.00 | 0.95 | 1.06 | 0.853 | 1.01 | 0.95 | 1.07 | 0.747 | 0.95 | 0.88 | 1.03 | 0.091 |
| Renal failure | 1.01 | 0.66 | 1.53 | 0.968 | 1.29 | 0.83 | 2.01 | 0.141 | 1.31 | 0.76 | 2.25 | 0.200 |
| Liver failure | 0.98 | 0.77 | 1.25 | 0.834 | 0.89 | 0.71 | 1.13 | 0.225 | 0.92 | 0.69 | 1.22 | 0.436 |
| Osteoporosis | 1.22 | 0.96 | 1.55 | 0.034 | 1.30 | 1.03 | 1.66 | 0.004 | 1.21 | 0.90 | 1.62 | 0.102 |
| Suicidality | 1.11 | 0.97 | 1.27 | 0.052 | 1.19 | 1.02 | 1.37 | 0.003 | 1.13 | 0.93 | 1.38 | 0.097 |
| Antihypertensive | 0.99 | 0.91 | 1.09 | 0.801 | 0.96 | 0.88 | 1.04 | 0.194 | 0.87 | 0.79 | 0.97 | 0.001 |
| Aspirin | 1.04 | 0.90 | 1.19 | 0.492 | 1.08 | 0.94 | 1.24 | 0.144 | 1.07 | 0.91 | 1.25 | 0.298 |
| Anticoagulants | 1.20 | 0.94 | 1.52 | 0.051 | 1.25 | 0.98 | 1.59 | 0.019 | 1.18 | 0.90 | 1.54 | 0.123 |
| NSAIDs | 1.06 | 0.92 | 1.21 | 0.308 | 0.96 | 0.84 | 1.10 | 0.456 | 0.93 | 0.78 | 1.09 | 0.232 |
| Anticonvulsants | 1.02 | 0.81 | 1.28 | 0.820 | 0.97 | 0.78 | 1.22 | 0.756 | 0.79 | 0.60 | 1.03 | 0.020 |
| Hypnotics | 1.11 | 1.02 | 1.21 | 0.001 | 1.11 | 1.03 | 1.21 | 0.001 | 1.04 | 0.94 | 1.16 | 0.329 |
| Bisphosphonates | 0.90 | 0.57 | 1.43 | 0.565 | 0.77 | 0.50 | 1.19 | 0.128 | 0.95 | 0.56 | 1.61 | 0.801 |
| Contraceptives | 0.94 | 0.86 | 1.03 | 0.093 | 0.88 | 0.81 | 0.96 | <0.001 | 0.91 | 0.80 | 1.03 | 0.047 |
| *Constant* | 0.72 | 0.45 | 1.17 | 0.082 | 2.79 | 1.59 | 4.89 | <0.001 | 17.19 | 8.60 | 34.32 | <0.001 |

|  | **Acceptability *(FSA, N = 673,177)*** | | | | | | | | | | | |
| --- | --- | --- | --- | --- | --- | --- | --- | --- | --- | --- | --- | --- |
|  | **2 months** | | | | **6 months** | | | | **12 months** | | | |
|  | *OR* | *99% CI* | | *P* | *OR* | *99% CI* | | *P* | *OR* | *99% CI* | | *P* |
| ***Statins*** | 0.88 | 0.85 | 0.91 | <0.001 | 0.81 | 0.79 | 0.84 | <0.001 | 0.78 | 0.75 | 0.81 | <0.001 |
| Antidepressant category (SSRIs) |  |  |  |  |  |  |  |  |  |  |  |  |
| TCAs | 2.03 | 1.95 | 2.12 | <0.001 | 2.39 | 2.28 | 2.51 | <0.001 | 2.04 | 1.92 | 2.18 | <0.001 |
| MAOIs | 1.23 | 0.69 | 2.17 | 0.358 | 1.97 | 0.94 | 4.11 | 0.018 | 1.01 | 0.42 | 2.42 | 0.967 |
| Other antidepressants | 1.42 | 1.37 | 1.49 | <0.001 | 1.58 | 1.52 | 1.64 | <0.001 | 1.47 | 1.41 | 1.54 | <0.001 |
|  |  |  |  |  |  |  |  |  |  |  |  |  |
| PHQ-9 baseline | 1.00 | 1.00 | 1.00 | 0.538 | 1.00 | 1.00 | 1.00 | <0.001 | 1.00 | 1.00 | 1.01 | <0.001 |
| BMI | 0.99 | 0.99 | 0.99 | <0.001 | 0.99 | 0.99 | 0.99 | <0.001 | 0.99 | 0.99 | 0.99 | <0.001 |
|  |  |  |  |  |  |  |  |  |  |  |  |  |
| Read codes for depression (Major depression) |  |  |  |  |  |  |  |  |  |  |  |  |
| Minor depression | 1.31 | 1.28 | 1.35 | <0.001 | 1.26 | 1.23 | 1.29 | <0.001 | 1.23 | 1.19 | 1.26 | <0.001 |
| Other | 0.97 | 0.92 | 1.02 | 0.162 | 0.99 | 0.95 | 1.04 | 0.757 | 0.97 | 0.91 | 1.03 | 0.207 |
|  |  |  |  |  |  |  |  |  |  |  |  |  |
| Townsend deprivation score in fifths (1, least deprived) |  |  |  |  |  |  |  |  |  |  |  |  |
| 2 | 1.06 | 1.04 | 1.08 | <0.001 | 1.11 | 1.08 | 1.13 | <0.001 | 1.06 | 1.03 | 1.10 | <0.001 |
| 3 | 1.13 | 1.10 | 1.16 | <0.001 | 1.23 | 1.19 | 1.26 | <0.001 | 1.16 | 1.12 | 1.20 | <0.001 |
| 4 | 1.21 | 1.17 | 1.25 | <0.001 | 1.35 | 1.31 | 1.40 | <0.001 | 1.29 | 1.24 | 1.35 | <0.001 |
| 5, most deprived | 1.25 | 1.20 | 1.30 | <0.001 | 1.45 | 1.38 | 1.51 | <0.001 | 1.38 | 1.31 | 1.46 | <0.001 |
|  |  |  |  |  |  |  |  |  |  |  |  |  |
| Region of England (East Midlands) |  |  |  |  |  |  |  |  |  |  |  |  |
| East of England | 1.00 | 0.83 | 1.22 | 0.949 | 0.97 | 0.80 | 1.17 | 0.642 | 0.91 | 0.72 | 1.15 | 0.284 |
| London | 1.04 | 0.86 | 1.26 | 0.569 | 1.02 | 0.85 | 1.22 | 0.787 | 0.97 | 0.78 | 1.19 | 0.670 |
| North East | 0.98 | 0.81 | 1.19 | 0.793 | 0.94 | 0.78 | 1.14 | 0.413 | 0.86 | 0.69 | 1.07 | 0.078 |
| North West | 1.08 | 0.90 | 1.30 | 0.273 | 1.02 | 0.85 | 1.21 | 0.825 | 0.87 | 0.71 | 1.07 | 0.080 |
| South Central | 1.02 | 0.85 | 1.23 | 0.765 | 1.01 | 0.85 | 1.20 | 0.893 | 0.97 | 0.79 | 1.19 | 0.675 |
| South East | 1.06 | 0.88 | 1.27 | 0.449 | 1.03 | 0.86 | 1.22 | 0.718 | 0.95 | 0.77 | 1.17 | 0.512 |
| South West | 1.01 | 0.84 | 1.22 | 0.838 | 1.02 | 0.85 | 1.21 | 0.818 | 0.96 | 0.78 | 1.18 | 0.615 |
| West Midlands | 1.10 | 0.92 | 1.33 | 0.179 | 1.06 | 0.89 | 1.27 | 0.392 | 0.94 | 0.77 | 1.15 | 0.437 |
| Yorkshire & Humber | 0.96 | 0.78 | 1.18 | 0.575 | 0.92 | 0.76 | 1.11 | 0.258 | 0.83 | 0.67 | 1.03 | 0.028 |
|  |  |  |  |  |  |  |  |  |  |  |  |  |
| Smoking status (Non-smoker) |  |  |  |  |  |  |  |  |  |  |  |  |
| Ex-smoker | 0.98 | 0.96 | 1.01 | 0.048 | 0.95 | 0.93 | 0.97 | <0.001 | 0.93 | 0.90 | 0.95 | <0.001 |
| Light-smoker | 1.18 | 1.16 | 1.21 | <0.001 | 1.33 | 1.30 | 1.36 | <0.001 | 1.27 | 1.24 | 1.31 | <0.001 |
| Moderate-smoker | 1.23 | 1.17 | 1.30 | <0.001 | 1.43 | 1.35 | 1.52 | <0.001 | 1.35 | 1.24 | 1.48 | <0.001 |
| Heavy-smoker | 1.21 | 1.12 | 1.30 | <0.001 | 1.44 | 1.32 | 1.58 | <0.001 | 1.36 | 1.19 | 1.55 | <0.001 |
|  |  |  |  |  |  |  |  |  |  |  |  |  |
| Alcohol use (Non-drinker/trivial) |  |  |  |  |  |  |  |  |  |  |  |  |
| Light | 0.98 | 0.96 | 1.00 | 0.002 | 0.96 | 0.94 | 0.98 | <0.001 | 0.96 | 0.94 | 0.99 | <0.001 |
| Medium | 1.00 | 0.97 | 1.03 | 0.755 | 0.99 | 0.95 | 1.03 | 0.496 | 0.98 | 0.93 | 1.03 | 0.240 |
| Heavy | 1.05 | 0.97 | 1.12 | 0.103 | 1.05 | 0.97 | 1.14 | 0.101 | 0.99 | 0.88 | 1.11 | 0.869 |
| Very heavy | 1.06 | 1.00 | 1.14 | 0.013 | 1.09 | 1.01 | 1.18 | 0.005 | 1.09 | 0.99 | 1.21 | 0.026 |
|  |  |  |  |  |  |  |  |  |  |  |  |  |
| Ethnic group (White) |  |  |  |  |  |  |  |  |  |  |  |  |
| African/Caribbean | 1.33 | 1.27 | 1.40 | <0.001 | 1.57 | 1.46 | 1.69 | <0.001 | 1.69 | 1.54 | 1.86 | <0.001 |
| Asian | 1.74 | 1.66 | 1.81 | <0.001 | 2.12 | 2.01 | 2.23 | <0.001 | 2.14 | 1.99 | 2.30 | <0.001 |
| Other | 1.43 | 1.37 | 1.50 | <0.001 | 1.66 | 1.58 | 1.76 | <0.001 | 1.88 | 1.72 | 2.05 | <0.001 |
|  |  |  |  |  |  |  |  |  |  |  |  |  |
| Age | 1.00 | 1.00 | 1.00 | <0.001 | 0.99 | 0.99 | 0.99 | <0.001 | 0.98 | 0.98 | 0.98 | <0.001 |
|  |  |  |  |  |  |  |  |  |  |  |  |  |
| Sex (Female) |  |  |  |  |  |  |  |  |  |  |  |  |
| Male | 1.00 | 0.98 | 1.01 | 0.892 | 1.14 | 1.12 | 1.16 | <0.001 | 1.16 | 1.13 | 1.19 | <0.001 |
|  |  |  |  |  |  |  |  |  |  |  |  |  |
| Year of diagnosis (1998-2005) |  |  |  |  |  |  |  |  |  |  |  |  |
| 2006 to 2010 | 0.88 | 0.86 | 0.90 | <0.001 | 0.82 | 0.80 | 0.84 | <0.001 | 0.70 | 0.68 | 0.73 | <0.001 |
| 2011 to 2015 | 0.80 | 0.78 | 0.82 | <0.001 | 0.73 | 0.71 | 0.75 | <0.001 | 0.56 | 0.54 | 0.59 | <0.001 |
| 2016 to 2020 | 0.84 | 0.82 | 0.86 | <0.001 | 0.78 | 0.75 | 0.80 | <0.001 | 0.65 | 0.62 | 0.68 | <0.001 |
|  |  |  |  |  |  |  |  |  |  |  |  |  |
| Coronary heart disease | 1.19 | 1.14 | 1.24 | <0.001 | 1.22 | 1.17 | 1.28 | <0.001 | 1.22 | 1.15 | 1.29 | <0.001 |
| Stroke | 0.97 | 0.92 | 1.02 | 0.088 | 1.02 | 0.98 | 1.07 | 0.190 | 0.96 | 0.91 | 1.02 | 0.096 |
| Diabetes | 1.00 | 0.97 | 1.04 | 0.791 | 1.00 | 0.96 | 1.04 | 0.976 | 0.96 | 0.91 | 1.00 | 0.012 |
| Epilepsy | 1.05 | 0.98 | 1.12 | 0.054 | 1.03 | 0.96 | 1.10 | 0.239 | 0.91 | 0.83 | 0.99 | 0.003 |
| Hypothyroidism | 0.96 | 0.93 | 1.00 | 0.014 | 0.89 | 0.85 | 0.92 | <0.001 | 0.86 | 0.82 | 0.90 | <0.001 |
| Arthritis | 1.07 | 1.04 | 1.11 | <0.001 | 1.13 | 1.10 | 1.17 | <0.001 | 1.15 | 1.11 | 1.20 | <0.001 |
| Anxiety | 1.00 | 0.98 | 1.02 | 0.984 | 0.89 | 0.87 | 0.91 | <0.001 | 0.83 | 0.80 | 0.85 | <0.001 |
| Migraine | 0.98 | 0.95 | 1.00 | 0.028 | 0.95 | 0.92 | 0.98 | <0.001 | 0.91 | 0.88 | 0.95 | <0.001 |
| Cancer | 1.10 | 1.07 | 1.13 | <0.001 | 1.10 | 1.07 | 1.14 | <0.001 | 1.14 | 1.10 | 1.18 | <0.001 |
| Asthma | 0.99 | 0.97 | 1.01 | 0.299 | 0.98 | 0.96 | 1.00 | 0.017 | 0.97 | 0.94 | 0.99 | 0.001 |
| Renal failure | 1.11 | 0.99 | 1.25 | 0.019 | 1.22 | 1.07 | 1.39 | <0.001 | 1.18 | 1.01 | 1.39 | 0.007 |
| Liver failure | 1.00 | 0.92 | 1.08 | 0.941 | 0.97 | 0.90 | 1.06 | 0.393 | 0.92 | 0.83 | 1.02 | 0.034 |
| Osteoporosis | 1.13 | 1.06 | 1.21 | <0.001 | 1.26 | 1.17 | 1.35 | <0.001 | 1.20 | 1.11 | 1.31 | <0.001 |
| Suicidality | 1.08 | 1.03 | 1.13 | <0.001 | 1.14 | 1.09 | 1.20 | <0.001 | 1.09 | 1.02 | 1.17 | 0.001 |
| Antihypertensive | 0.97 | 0.95 | 1.00 | 0.023 | 0.92 | 0.89 | 0.95 | <0.001 | 0.84 | 0.81 | 0.88 | <0.001 |
| Aspirin | 0.98 | 0.94 | 1.02 | 0.131 | 1.01 | 0.97 | 1.06 | 0.403 | 0.98 | 0.94 | 1.03 | 0.354 |
| Anticoagulants | 1.09 | 1.02 | 1.16 | 0.001 | 1.13 | 1.06 | 1.21 | <0.001 | 1.14 | 1.05 | 1.23 | <0.001 |
| NSAIDs | 1.02 | 0.98 | 1.06 | 0.144 | 0.98 | 0.94 | 1.02 | 0.142 | 0.89 | 0.84 | 0.93 | <0.001 |
| Anticonvulsants | 0.89 | 0.83 | 0.94 | <0.001 | 0.76 | 0.71 | 0.81 | <0.001 | 0.70 | 0.65 | 0.75 | <0.001 |
| Hypnotics | 1.08 | 1.06 | 1.11 | <0.001 | 1.12 | 1.09 | 1.15 | <0.001 | 1.08 | 1.04 | 1.13 | <0.001 |
| Bisphosphonates | 0.90 | 0.80 | 1.01 | 0.017 | 0.90 | 0.80 | 1.02 | 0.024 | 0.91 | 0.79 | 1.05 | 0.105 |
| Contraceptives | 0.88 | 0.85 | 0.90 | <0.001 | 0.82 | 0.79 | 0.85 | <0.001 | 0.86 | 0.82 | 0.90 | <0.001 |
| *Constant* | 0.61 | 0.50 | 0.75 | <0.001 | 3.28 | 2.71 | 3.97 | <0.001 | 23.42 | 18.72 | 29.29 | <0.001 |

### **Table A10b.** Regression analyses for tolerability at 2 months, 6 months, and 12 months; complete case analysis (CCA) and full set analysis (FSA)

|  | **Tolerability *(CCA, N= 63,801)*** | | | | | | | | | | | |
| --- | --- | --- | --- | --- | --- | --- | --- | --- | --- | --- | --- | --- |
|  | **2 months** | | | | **6 months** | | | | **12 months** | | | |
|  | *OR* | *99% CI* | | *P* | *OR* | *99% CI* | | *P* | *OR* | *99% CI* | | *P* |
| ***Statins*** | 1.02 | 0.83 | 1.26 | 0.795 | 1.07 | 0.90 | 1.26 | 0.316 | 1.11 | 0.97 | 1.28 | 0.052 |
| Antidepressant category (SSRIs) |  |  |  |  |  |  |  |  |  |  |  |  |
| TCAs | 2.80 | 2.20 | 3.57 | <0.001 | 2.05 | 1.67 | 2.52 | <0.001 | 1.37 | 1.13 | 1.67 | <0.001 |
| MAOIs |  |  |  |  |  |  |  |  |  |  |  |  |
| Other antidepressants | 1.51 | 1.22 | 1.86 | <0.001 | 1.45 | 1.23 | 1.71 | <0.001 | 1.24 | 1.07 | 1.44 | <0.001 |
|  |  |  |  |  |  |  |  |  |  |  |  |  |
| PHQ-9 baseline | 1.01 | 1.00 | 1.02 | 0.066 | 1.01 | 1.00 | 1.02 | 0.018 | 1.01 | 1.00 | 1.01 | 0.034 |
| BMI | 0.99 | 0.98 | 1.00 | 0.042 | 1.00 | 0.99 | 1.00 | 0.313 | 1.00 | 1.00 | 1.01 | 0.417 |
|  |  |  |  |  |  |  |  |  |  |  |  |  |
| Read codes for depression (Major depression) |  |  |  |  |  |  |  |  |  |  |  |  |
| Minor depression | 1.58 | 1.40 | 1.78 | <0.001 | 1.39 | 1.27 | 1.52 | <0.001 | 1.26 | 1.17 | 1.36 | <0.001 |
| Other | 1.09 | 0.74 | 1.61 | 0.560 | 0.96 | 0.70 | 1.32 | 0.749 | 0.97 | 0.74 | 1.27 | 0.763 |
|  |  |  |  |  |  |  |  |  |  |  |  |  |
| Townsend deprivation score in fifths (1, least deprived) |  |  |  |  |  |  |  |  |  |  |  |  |
| 2 | 1.02 | 0.86 | 1.20 | 0.765 | 1.06 | 0.93 | 1.20 | 0.272 | 1.06 | 0.96 | 1.18 | 0.136 |
| 3 | 1.09 | 0.93 | 1.28 | 0.160 | 1.15 | 1.01 | 1.31 | 0.004 | 1.11 | 1.00 | 1.23 | 0.012 |
| 4 | 1.07 | 0.89 | 1.28 | 0.346 | 1.16 | 1.01 | 1.34 | 0.004 | 1.13 | 1.00 | 1.27 | 0.011 |
| 5, most deprived | 1.22 | 1.00 | 1.48 | 0.008 | 1.25 | 1.07 | 1.45 | <0.001 | 1.16 | 1.02 | 1.33 | 0.004 |
|  |  |  |  |  |  |  |  |  |  |  |  |  |
| Region of England (East Midlands) |  |  |  |  |  |  |  |  |  |  |  |  |
| East of England | 1.03 | 0.69 | 1.56 | 0.836 | 0.80 | 0.58 | 1.11 | 0.076 | 0.79 | 0.56 | 1.10 | 0.069 |
| London | 0.92 | 0.66 | 1.29 | 0.524 | 0.80 | 0.63 | 1.02 | 0.018 | 0.80 | 0.62 | 1.03 | 0.025 |
| North East | 1.16 | 0.79 | 1.72 | 0.313 | 0.99 | 0.76 | 1.30 | 0.930 | 1.00 | 0.75 | 1.34 | 0.996 |
| North West | 1.10 | 0.78 | 1.55 | 0.485 | 0.98 | 0.77 | 1.25 | 0.828 | 0.96 | 0.74 | 1.24 | 0.655 |
| South Central | 1.04 | 0.71 | 1.51 | 0.795 | 0.90 | 0.69 | 1.16 | 0.278 | 0.80 | 0.61 | 1.05 | 0.032 |
| South East | 1.00 | 0.71 | 1.42 | 0.989 | 0.94 | 0.73 | 1.22 | 0.557 | 0.92 | 0.70 | 1.20 | 0.404 |
| South West | 0.98 | 0.68 | 1.42 | 0.883 | 0.87 | 0.65 | 1.15 | 0.192 | 0.88 | 0.66 | 1.17 | 0.245 |
| West Midlands | 1.02 | 0.72 | 1.45 | 0.883 | 0.92 | 0.71 | 1.21 | 0.441 | 0.87 | 0.66 | 1.16 | 0.218 |
| Yorkshire & Humber | 0.97 | 0.66 | 1.44 | 0.865 | 0.87 | 0.63 | 1.19 | 0.249 | 0.94 | 0.69 | 1.28 | 0.630 |
|  |  |  |  |  |  |  |  |  |  |  |  |  |
| Smoking status (Non-smoker) |  |  |  |  |  |  |  |  |  |  |  |  |
| Ex-smoker | 1.02 | 0.89 | 1.16 | 0.739 | 0.95 | 0.86 | 1.06 | 0.233 | 1.00 | 0.92 | 1.09 | 0.889 |
| Light-smoker | 1.07 | 0.94 | 1.21 | 0.195 | 1.05 | 0.95 | 1.16 | 0.199 | 0.98 | 0.90 | 1.07 | 0.581 |
| Moderate-smoker | 1.09 | 0.71 | 1.68 | 0.602 | 1.03 | 0.72 | 1.47 | 0.840 | 0.97 | 0.72 | 1.31 | 0.787 |
| Heavy-smoker | 1.09 | 0.59 | 2.00 | 0.722 | 0.90 | 0.54 | 1.48 | 0.570 | 0.80 | 0.52 | 1.23 | 0.173 |
|  |  |  |  |  |  |  |  |  |  |  |  |  |
| Alcohol use (Non-drinker/trivial) |  |  |  |  |  |  |  |  |  |  |  |  |
| Light | 1.00 | 0.89 | 1.13 | 0.978 | 1.04 | 0.95 | 1.14 | 0.244 | 1.02 | 0.95 | 1.10 | 0.464 |
| Medium | 0.82 | 0.67 | 1.01 | 0.012 | 0.91 | 0.78 | 1.06 | 0.106 | 0.93 | 0.82 | 1.05 | 0.127 |
| Heavy | 1.18 | 0.80 | 1.74 | 0.278 | 0.90 | 0.64 | 1.27 | 0.425 | 0.79 | 0.58 | 1.08 | 0.051 |
| Very heavy | 0.85 | 0.56 | 1.29 | 0.321 | 0.94 | 0.68 | 1.28 | 0.584 | 0.87 | 0.66 | 1.15 | 0.208 |
|  |  |  |  |  |  |  |  |  |  |  |  |  |
| Ethnic group (White) |  |  |  |  |  |  |  |  |  |  |  |  |
| African/Caribbean | 1.26 | 0.91 | 1.76 | 0.070 | 1.30 | 0.99 | 1.71 | 0.012 | 1.02 | 0.80 | 1.31 | 0.797 |
| Asian | 1.31 | 0.98 | 1.75 | 0.017 | 1.27 | 1.00 | 1.60 | 0.009 | 1.06 | 0.86 | 1.30 | 0.511 |
| Other | 1.28 | 0.95 | 1.72 | 0.036 | 1.13 | 0.88 | 1.44 | 0.205 | 1.00 | 0.82 | 1.23 | 0.986 |
|  |  |  |  |  |  |  |  |  |  |  |  |  |
| Age | 1.01 | 1.00 | 1.01 | <0.001 | 1.01 | 1.00 | 1.01 | <0.001 | 1.01 | 1.01 | 1.01 | <0.001 |
|  |  |  |  |  |  |  |  |  |  |  |  |  |
| Sex (Female) |  |  |  |  |  |  |  |  |  |  |  |  |
| Male | 0.94 | 0.84 | 1.05 | 0.165 | 0.94 | 0.86 | 1.03 | 0.069 | 0.89 | 0.83 | 0.96 | <0.001 |
|  |  |  |  |  |  |  |  |  |  |  |  |  |
| Year of diagnosis (1998-2005) |  |  |  |  |  |  |  |  |  |  |  |  |
| 2006 to 2010 | 0.89 | 0.18 | 4.36 | 0.850 | 1.18 | 0.33 | 4.25 | 0.737 | 1.01 | 0.40 | 2.53 | 0.974 |
| 2011 to 2015 | 0.89 | 0.18 | 4.38 | 0.847 | 1.17 | 0.33 | 4.22 | 0.746 | 1.02 | 0.41 | 2.56 | 0.953 |
| 2016 to 2020 | 1.24 | 0.25 | 6.10 | 0.729 | 1.49 | 0.41 | 5.34 | 0.424 | 1.15 | 0.46 | 2.90 | 0.691 |
|  |  |  |  |  |  |  |  |  |  |  |  |  |
| Coronary heart disease | 1.33 | 0.99 | 1.80 | 0.014 | 1.20 | 0.95 | 1.52 | 0.048 | 1.11 | 0.92 | 1.35 | 0.143 |
| Stroke | 1.10 | 0.77 | 1.57 | 0.512 | 1.21 | 0.93 | 1.57 | 0.062 | 1.10 | 0.89 | 1.37 | 0.226 |
| Diabetes | 1.22 | 0.96 | 1.54 | 0.032 | 1.15 | 0.95 | 1.38 | 0.059 | 1.16 | 1.00 | 1.34 | 0.010 |
| Epilepsy | 0.89 | 0.54 | 1.46 | 0.540 | 0.98 | 0.67 | 1.44 | 0.910 | 1.04 | 0.77 | 1.39 | 0.761 |
| Hypothyroidism | 1.08 | 0.82 | 1.42 | 0.473 | 1.04 | 0.84 | 1.30 | 0.609 | 1.07 | 0.90 | 1.27 | 0.294 |
| Arthritis | 0.96 | 0.79 | 1.18 | 0.650 | 1.03 | 0.89 | 1.20 | 0.597 | 1.09 | 0.96 | 1.23 | 0.076 |
| Anxiety | 1.34 | 1.17 | 1.55 | <0.001 | 1.31 | 1.19 | 1.46 | <0.001 | 1.39 | 1.27 | 1.51 | <0.001 |
| Migraine | 1.00 | 0.82 | 1.23 | 0.950 | 1.06 | 0.91 | 1.23 | 0.347 | 1.10 | 0.98 | 1.25 | 0.036 |
| Cancer | 1.20 | 1.00 | 1.45 | 0.010 | 1.20 | 1.05 | 1.38 | 0.001 | 1.17 | 1.04 | 1.31 | <0.001 |
| Asthma | 1.18 | 1.03 | 1.35 | 0.001 | 1.22 | 1.10 | 1.35 | <0.001 | 1.26 | 1.16 | 1.37 | <0.001 |
| Renal failure | 0.88 | 0.36 | 2.15 | 0.715 | 1.12 | 0.59 | 2.10 | 0.648 | 1.10 | 0.64 | 1.87 | 0.659 |
| Liver failure | 1.03 | 0.58 | 1.83 | 0.905 | 1.19 | 0.80 | 1.76 | 0.252 | 1.32 | 0.97 | 1.79 | 0.021 |
| Osteoporosis | 0.81 | 0.47 | 1.38 | 0.309 | 0.91 | 0.61 | 1.36 | 0.562 | 0.99 | 0.72 | 1.35 | 0.909 |
| Suicidality | 1.07 | 0.78 | 1.46 | 0.595 | 1.16 | 0.93 | 1.45 | 0.079 | 1.13 | 0.94 | 1.37 | 0.082 |
| Antihypertensive | 0.89 | 0.73 | 1.07 | 0.105 | 1.01 | 0.87 | 1.17 | 0.913 | 0.99 | 0.88 | 1.13 | 0.916 |
| Aspirin | 0.93 | 0.68 | 1.28 | 0.578 | 0.97 | 0.78 | 1.21 | 0.729 | 0.98 | 0.82 | 1.18 | 0.822 |
| Anticoagulants | 1.24 | 0.78 | 1.95 | 0.233 | 1.27 | 0.88 | 1.84 | 0.094 | 1.27 | 0.94 | 1.72 | 0.040 |
| NSAIDs | 1.05 | 0.80 | 1.38 | 0.635 | 1.10 | 0.87 | 1.38 | 0.304 | 1.11 | 0.92 | 1.34 | 0.153 |
| Anticonvulsants | 1.33 | 0.86 | 2.07 | 0.095 | 1.25 | 0.86 | 1.81 | 0.119 | 1.29 | 0.95 | 1.75 | 0.035 |
| Hypnotics | 1.19 | 1.00 | 1.43 | 0.011 | 1.10 | 0.96 | 1.26 | 0.075 | 1.09 | 0.97 | 1.22 | 0.070 |
| Bisphosphonates | 1.30 | 0.54 | 3.12 | 0.443 | 1.18 | 0.59 | 2.34 | 0.536 | 1.26 | 0.71 | 2.22 | 0.302 |
| Contraceptives | 0.98 | 0.78 | 1.24 | 0.838 | 1.00 | 0.85 | 1.19 | 0.974 | 1.08 | 0.95 | 1.23 | 0.144 |
| *Constant* | 0.02 | 0.00 | 0.12 | <0.001 | 0.03 | 0.01 | 0.13 | <0.001 | 0.06 | 0.02 | 0.16 | <0.001 |

|  | **Tolerability *(FSA, N = 673,177)*** | | | | | | | | | | | |
| --- | --- | --- | --- | --- | --- | --- | --- | --- | --- | --- | --- | --- |
|  | **2 months** | | | | **6 months** | | | | **12 months** | | | |
|  | *OR* | *99% CI* | | *P* | *OR* | *99% CI* | | *P* | *OR* | *99% CI* | | *P* |
| ***Statins*** | 0.92 | 0.87 | 0.98 | 0.001 | 0.94 | 0.89 | 0.99 | 0.001 | 1.02 | 0.97 | 1.06 | 0.351 |
| Antidepressant category (SSRIs) |  |  |  |  |  |  |  |  |  |  |  |  |
| TCAs | 2.34 | 2.205 | 2.47 | 0.00 | 1.85 | 1.77 | 1.95 | <0.001 | 1.44 | 1.37 | 1.51 | <0.001 |
| MAOIs | 0.50 | 0.31 | 2.96 | 0.084 | 2.14 | 0.98 | 4.65 | 0.012 | 1.48 | 0.70 | 3.16 | 0.179 |
| Other antidepressants | 1.38 | 1.309 | 1.46 | 0.001 | 1.29 | 1.23 | 1.35 | <0.001 | 1.15 | 1.11 | 1.20 | <0.001 |
|  |  |  |  |  |  |  |  |  |  |  |  |  |
| PHQ-9 baseline | 1.00 | 0.998 | 1.01 | 0.13 | 1.00 | 1.00 | 1.01 | 0.031 | 1.00 | 1.00 | 1.00 | 0.083 |
| BMI | 0.99 | 0.990 | 1.00 | 0.00 | 1.00 | 0.99 | 1.00 | <0.001 | 1.00 | 1.00 | 1.00 | 0.466 |
|  |  |  |  |  |  |  |  |  |  |  |  |  |
| Read codes for depression (Major depression) |  |  |  |  |  |  |  |  |  |  |  |  |
| Minor depression | 1.43 | 1.375 | 1.48 | 0.00 | 1.33 | 1.29 | 1.37 | <0.001 | 1.25 | 1.22 | 1.28 | <0.001 |
| Other | 0.73 | 0.654 | 0.81 | 0.00 | 0.75 | 0.69 | 0.82 | <0.001 | 0.79 | 0.73 | 0.86 | <0.001 |
|  |  |  |  |  |  |  |  |  |  |  |  |  |
| Townsend deprivation score in fifths (1, least deprived) |  |  |  |  |  |  |  |  |  |  |  |  |
| 2 | 1.03 | 0.989 | 1.08 | 0.05 | 1.04 | 1.01 | 1.08 | 0.003 | 1.03 | 1.00 | 1.06 | 0.017 |
| 3 | 1.11 | 1.059 | 1.16 | 0.00 | 1.11 | 1.07 | 1.16 | <0.001 | 1.06 | 1.02 | 1.10 | <0.001 |
| 4 | 1.13 | 1.067 | 1.19 | 0.00 | 1.13 | 1.07 | 1.18 | <0.001 | 1.05 | 1.01 | 1.10 | 0.002 |
| 5, most deprived | 1.21 | 1.135 | 1.29 | 0.00 | 1.19 | 1.12 | 1.25 | <0.001 | 1.10 | 1.04 | 1.15 | <0.001 |
|  |  |  |  |  |  |  |  |  |  |  |  |  |
| Region of England (East Midlands) |  |  |  |  |  |  |  |  |  |  |  |  |
| East of England | 0.88 | 0.737 | 1.06 | 0.08 | 0.91 | 0.78 | 1.07 | 0.128 | 0.93 | 0.78 | 1.12 | 0.341 |
| London | 0.91 | 0.792 | 1.04 | 0.07 | 0.88 | 0.80 | 0.97 | 0.001 | 0.86 | 0.77 | 0.95 | <0.001 |
| North East | 0.98 | 0.810 | 1.18 | 0.74 | 0.99 | 0.86 | 1.15 | 0.901 | 1.01 | 0.86 | 1.19 | 0.859 |
| North West | 1.03 | 0.901 | 1.18 | 0.55 | 1.02 | 0.93 | 1.13 | 0.594 | 1.00 | 0.90 | 1.11 | 0.965 |
| South Central | 0.92 | 0.785 | 1.07 | 0.15 | 0.91 | 0.80 | 1.02 | 0.033 | 0.87 | 0.77 | 0.99 | 0.006 |
| South East | 0.99 | 0.853 | 1.14 | 0.79 | 0.96 | 0.85 | 1.07 | 0.308 | 0.93 | 0.82 | 1.06 | 0.142 |
| South West | 1.02 | 0.881 | 1.18 | 0.71 | 1.04 | 0.93 | 1.16 | 0.390 | 1.02 | 0.90 | 1.15 | 0.730 |
| West Midlands | 0.96 | 0.834 | 1.11 | 0.50 | 0.95 | 0.85 | 1.05 | 0.178 | 0.93 | 0.82 | 1.04 | 0.090 |
| Yorkshire & Humber | 0.94 | 0.785 | 1.13 | 0.40 | 0.94 | 0.82 | 1.09 | 0.275 | 0.99 | 0.85 | 1.15 | 0.879 |
|  |  |  |  |  |  |  |  |  |  |  |  |  |
| Smoking status (Non-smoker) |  |  |  |  |  |  |  |  |  |  |  |  |
| Ex-smoker | 1.00 | 0.963 | 1.04 | 0.89 | 1.00 | 0.96 | 1.03 | 0.770 | 1.03 | 1.00 | 1.06 | 0.011 |
| Light-smoker | 1.06 | 1.023 | 1.10 | 0.00 | 1.07 | 1.04 | 1.10 | <0.001 | 1.00 | 0.98 | 1.03 | 0.813 |
| Moderate-smoker | 0.99 | 0.876 | 1.11 | 0.75 | 0.99 | 0.90 | 1.10 | 0.837 | 0.92 | 0.84 | 1.01 | 0.021 |
| Heavy-smoker | 1.09 | 0.934 | 1.28 | 0.15 | 1.08 | 0.95 | 1.22 | 0.127 | 0.98 | 0.87 | 1.10 | 0.639 |
|  |  |  |  |  |  |  |  |  |  |  |  |  |
| Alcohol use (Non-drinker/trivial) |  |  |  |  |  |  |  |  |  |  |  |  |
| Light | 0.99 | 0.955 | 1.02 | 0.39 | 0.99 | 0.96 | 1.02 | 0.254 | 0.99 | 0.97 | 1.01 | 0.255 |
| Medium | 0.99 | 0.937 | 1.06 | 0.82 | 1.00 | 0.95 | 1.05 | 0.867 | 1.00 | 0.95 | 1.04 | 0.897 |
| Heavy | 1.02 | 0.880 | 1.19 | 0.68 | 0.98 | 0.86 | 1.11 | 0.621 | 0.95 | 0.85 | 1.08 | 0.298 |
| Very heavy | 0.94 | 0.815 | 1.09 | 0.28 | 0.97 | 0.85 | 1.10 | 0.472 | 0.94 | 0.84 | 1.05 | 0.150 |
|  |  |  |  |  |  |  |  |  |  |  |  |  |
| Ethnic group (White) |  |  |  |  |  |  |  |  |  |  |  |  |
| African/Caribbean | 1.15 | 1.064 | 1.24 | 0.00 | 1.12 | 1.06 | 1.18 | <0.001 | 1.00 | 0.95 | 1.05 | 0.932 |
| Asian | 1.45 | 1.362 | 1.55 | 0.00 | 1.35 | 1.28 | 1.43 | <0.001 | 1.18 | 1.12 | 1.24 | <0.001 |
| Other | 1.30 | 1.196 | 1.41 | 0.00 | 1.22 | 1.14 | 1.31 | <0.001 | 1.09 | 1.02 | 1.15 | <0.001 |
|  |  |  |  |  |  |  |  |  |  |  |  |  |
| Age | 1.01 | 1.008 | 1.01 | 0.00 | 1.01 | 1.01 | 1.01 | <0.001 | 1.01 | 1.01 | 1.01 | <0.001 |
|  |  |  |  |  |  |  |  |  |  |  |  |  |
| Sex (Female) |  |  |  |  |  |  |  |  |  |  |  |  |
| Male | 0.93 | 0.900 | 0.96 | 0.00 | 0.91 | 0.89 | 0.93 | <0.001 | 0.86 | 0.84 | 0.88 | <0.001 |
|  |  |  |  |  |  |  |  |  |  |  |  |  |
| Year of diagnosis (1998-2005) |  |  |  |  |  |  |  |  |  |  |  |  |
| 2006 to 2010 | 1.01 | 0.957 | 1.06 | 0.78 | 0.99 | 0.95 | 1.03 | 0.413 | 1.04 | 0.99 | 1.08 | 0.026 |
| 2011 to 2015 | 1.04 | 0.985 | 1.10 | 0.06 | 1.01 | 0.96 | 1.06 | 0.661 | 1.07 | 1.02 | 1.12 | <0.001 |
| 2016 to 2020 | 1.21 | 1.143 | 1.27 | 0.00 | 1.14 | 1.09 | 1.20 | <0.001 | 1.12 | 1.07 | 1.18 | <0.001 |
|  |  |  |  |  |  |  |  |  |  |  |  |  |
| Coronary heart disease | 1.16 | 1.071 | 1.25 | 0.00 | 1.15 | 1.08 | 1.23 | <0.001 | 1.11 | 1.05 | 1.17 | <0.001 |
| Stroke | 1.03 | 0.958 | 1.11 | 0.27 | 1.13 | 1.06 | 1.19 | <0.001 | 1.14 | 1.08 | 1.20 | <0.001 |
| Diabetes | 1.08 | 1.010 | 1.15 | 0.00 | 1.13 | 1.06 | 1.19 | <0.001 | 1.14 | 1.09 | 1.20 | <0.001 |
| Epilepsy | 1.01 | 0.887 | 1.14 | 0.91 | 1.03 | 0.93 | 1.13 | 0.474 | 1.07 | 0.98 | 1.16 | 0.042 |
| Hypothyroidism | 1.02 | 0.949 | 1.09 | 0.50 | 1.02 | 0.96 | 1.08 | 0.412 | 1.06 | 1.01 | 1.12 | 0.001 |
| Arthritis | 1.08 | 1.021 | 1.14 | 0.00 | 1.10 | 1.05 | 1.14 | <0.001 | 1.08 | 1.04 | 1.12 | <0.001 |
| Anxiety | 1.24 | 1.187 | 1.30 | 0.00 | 1.21 | 1.17 | 1.26 | <0.001 | 1.27 | 1.23 | 1.31 | <0.001 |
| Migraine | 1.08 | 1.018 | 1.14 | 0.00 | 1.09 | 1.04 | 1.14 | <0.001 | 1.14 | 1.10 | 1.18 | <0.001 |
| Cancer | 1.21 | 1.156 | 1.27 | 0.00 | 1.23 | 1.19 | 1.28 | <0.001 | 1.18 | 1.14 | 1.22 | <0.001 |
| Asthma | 1.14 | 1.098 | 1.18 | 0.00 | 1.19 | 1.15 | 1.22 | <0.001 | 1.25 | 1.22 | 1.28 | <0.001 |
| Renal failure | 1.24 | 1.023 | 1.51 | 0.00 | 1.29 | 1.09 | 1.52 | <0.001 | 1.23 | 1.07 | 1.42 | <0.001 |
| Liver failure | 1.14 | 0.991 | 1.32 | 0.02 | 1.20 | 1.07 | 1.34 | <0.001 | 1.21 | 1.10 | 1.33 | <0.001 |
| Osteoporosis | 1.08 | 0.965 | 1.20 | 0.08 | 1.13 | 1.04 | 1.24 | <0.001 | 1.10 | 1.02 | 1.19 | 0.001 |
| Suicidality | 1.19 | 1.099 | 1.30 | 0.00 | 1.21 | 1.13 | 1.29 | <0.001 | 1.16 | 1.10 | 1.24 | <0.001 |
| Antihypertensive | 1.01 | 0.954 | 1.07 | 0.68 | 0.99 | 0.95 | 1.04 | 0.594 | 1.01 | 0.97 | 1.05 | 0.514 |
| Aspirin | 1.05 | 0.975 | 1.13 | 0.09 | 1.09 | 1.03 | 1.16 | <0.001 | 1.08 | 1.03 | 1.14 | <0.001 |
| Anticoagulants | 1.26 | 1.142 | 1.39 | 0.00 | 1.37 | 1.26 | 1.49 | <0.001 | 1.36 | 1.26 | 1.46 | <0.001 |
| NSAIDs | 1.17 | 1.090 | 1.25 | 0.00 | 1.16 | 1.10 | 1.22 | <0.001 | 1.17 | 1.11 | 1.23 | <0.001 |
| Anticonvulsants | 1.16 | 1.040 | 1.28 | 0.00 | 1.19 | 1.09 | 1.29 | <0.001 | 1.32 | 1.22 | 1.41 | <0.001 |
| Hypnotics | 1.19 | 1.135 | 1.25 | 0.00 | 1.17 | 1.12 | 1.21 | <0.001 | 1.13 | 1.09 | 1.17 | <0.001 |
| Bisphosphonates | 1.05 | 0.879 | 1.26 | 0.48 | 1.10 | 0.94 | 1.28 | 0.123 | 1.16 | 1.02 | 1.32 | 0.002 |
| Contraceptives | 0.95 | 0.885 | 1.01 | 0.03 | 0.99 | 0.94 | 1.04 | 0.539 | 1.07 | 1.03 | 1.12 | <0.001 |
| *Constant* | 0.03 | 0.023 | 0.03 | 0.00 | 0.05 | 0.05 | 0.06 | <0.001 | 0.07 | 0.06 | 0.08 | <0.001 |

### **Table A10c.** Regression analyses for safety (any adverse event) at 2 months, 6 months, and 12 months; complete case analysis (CCA) and full set analysis (FSA)

|  | **Safety, any adverse event *(CCA, N= 63,801)*** | | | | | | | | | | | |
| --- | --- | --- | --- | --- | --- | --- | --- | --- | --- | --- | --- | --- |
|  | **2 months** | | | | **6 months** | | | | **12 months** | | | |
|  | *OR* | *99% CI* | | *P* | *OR* | *99% CI* | | *P* | *OR* | *99% CI* | | *P* |
| ***Statins*** | 0.94 | 0.85 | 1.05 | 0.140 | 0.98 | 0.88 | 1.09 | 0.605 | 1.00 | 0.89 | 1.12 | 0.961 |
| Antidepressant category (SSRIs) |  |  |  |  |  |  |  |  |  |  |  |  |
| TCAs | 1.02 | 0.87 | 1.20 | 0.767 | 1.05 | 0.90 | 1.22 | 0.444 | 1.07 | 0.90 | 1.27 | 0.314 |
| MAOIs |  |  |  |  |  |  |  |  |  |  |  |  |
| Other antidepressants | 1.06 | 0.95 | 1.19 | 0.170 | 1.07 | 0.96 | 1.20 | 0.119 | 1.07 | 0.96 | 1.19 | 0.112 |
|  |  |  |  |  |  |  |  |  |  |  |  |  |
| PHQ-9 baseline | 1.00 | 1.00 | 1.01 | 0.419 | 1.00 | 0.99 | 1.00 | 0.182 | 1.00 | 0.99 | 1.00 | 0.433 |
| BMI | 1.00 | 0.99 | 1.00 | 0.021 | 1.00 | 1.00 | 1.00 | 0.757 | 1.00 | 1.00 | 1.01 | 0.057 |
|  |  |  |  |  |  |  |  |  |  |  |  |  |
| Read codes for depression (Major depression) |  |  |  |  |  |  |  |  |  |  |  |  |
| Minor depression | 1.93 | 1.78 | 2.09 | <0.001 | 1.83 | 1.69 | 1.97 | <0.001 | 1.75 | 1.60 | 1.90 | <0.001 |
| Other | 0.57 | 0.46 | 0.70 | <0.001 | 0.65 | 0.54 | 0.79 | <0.001 | 0.70 | 0.58 | 0.84 | <0.001 |
|  |  |  |  |  |  |  |  |  |  |  |  |  |
| Townsend deprivation score in fifths (1, least deprived) |  |  |  |  |  |  |  |  |  |  |  |  |
| 2 | 0.98 | 0.91 | 1.05 | 0.484 | 1.00 | 0.93 | 1.07 | 0.941 | 1.01 | 0.94 | 1.08 | 0.825 |
| 3 | 1.05 | 0.96 | 1.14 | 0.161 | 1.05 | 0.97 | 1.14 | 0.121 | 1.05 | 0.96 | 1.15 | 0.186 |
| 4 | 1.04 | 0.94 | 1.16 | 0.299 | 1.10 | 0.99 | 1.22 | 0.019 | 1.09 | 0.98 | 1.21 | 0.032 |
| 5, most deprived | 1.11 | 0.99 | 1.24 | 0.023 | 1.15 | 1.03 | 1.29 | 0.001 | 1.15 | 1.02 | 1.29 | 0.002 |
|  |  |  |  |  |  |  |  |  |  |  |  |  |
| Region of England (East Midlands) |  |  |  |  |  |  |  |  |  |  |  |  |
| East of England | 0.68 | 0.41 | 1.11 | 0.042 | 0.70 | 0.43 | 1.15 | 0.066 | 0.72 | 0.43 | 1.21 | 0.106 |
| London | 0.75 | 0.51 | 1.09 | 0.049 | 0.79 | 0.55 | 1.13 | 0.088 | 0.80 | 0.57 | 1.14 | 0.107 |
| North East | 0.80 | 0.51 | 1.24 | 0.185 | 0.84 | 0.54 | 1.31 | 0.318 | 0.93 | 0.60 | 1.43 | 0.664 |
| North West | 0.83 | 0.56 | 1.23 | 0.223 | 0.87 | 0.60 | 1.27 | 0.357 | 0.93 | 0.64 | 1.34 | 0.604 |
| South Central | 0.66 | 0.44 | 0.99 | 0.009 | 0.70 | 0.47 | 1.03 | 0.018 | 0.73 | 0.50 | 1.08 | 0.037 |
| South East | 0.82 | 0.54 | 1.24 | 0.216 | 0.89 | 0.59 | 1.33 | 0.459 | 0.91 | 0.61 | 1.34 | 0.511 |
| South West | 0.77 | 0.51 | 1.17 | 0.105 | 0.81 | 0.54 | 1.22 | 0.190 | 0.86 | 0.57 | 1.29 | 0.323 |
| West Midlands | 0.72 | 0.47 | 1.11 | 0.051 | 0.78 | 0.52 | 1.17 | 0.109 | 0.82 | 0.56 | 1.22 | 0.200 |
| Yorkshire & Humber | 0.90 | 0.56 | 1.44 | 0.558 | 0.90 | 0.57 | 1.42 | 0.540 | 0.92 | 0.58 | 1.44 | 0.625 |
|  |  |  |  |  |  |  |  |  |  |  |  |  |
| Smoking status (Non-smoker) |  |  |  |  |  |  |  |  |  |  |  |  |
| Ex-smoker | 1.04 | 0.98 | 1.11 | 0.065 | 1.04 | 0.98 | 1.11 | 0.056 | 1.04 | 0.97 | 1.11 | 0.143 |
| Light-smoker | 0.94 | 0.89 | 1.00 | 0.006 | 0.97 | 0.92 | 1.03 | 0.163 | 0.98 | 0.92 | 1.04 | 0.385 |
| Moderate-smoker | 0.94 | 0.77 | 1.16 | 0.458 | 0.96 | 0.80 | 1.16 | 0.561 | 0.96 | 0.79 | 1.17 | 0.596 |
| Heavy-smoker | 0.99 | 0.76 | 1.28 | 0.898 | 0.89 | 0.69 | 1.15 | 0.240 | 0.82 | 0.62 | 1.08 | 0.068 |
|  |  |  |  |  |  |  |  |  |  |  |  |  |
| Alcohol use (Non-drinker/trivial) |  |  |  |  |  |  |  |  |  |  |  |  |
| Light | 1.02 | 0.97 | 1.08 | 0.359 | 1.02 | 0.97 | 1.08 | 0.296 | 1.01 | 0.96 | 1.07 | 0.612 |
| Medium | 1.00 | 0.93 | 1.09 | 0.874 | 1.06 | 0.98 | 1.15 | 0.064 | 1.04 | 0.95 | 1.13 | 0.271 |
| Heavy | 1.05 | 0.87 | 1.26 | 0.529 | 0.96 | 0.80 | 1.16 | 0.618 | 0.92 | 0.76 | 1.12 | 0.285 |
| Very heavy | 0.97 | 0.82 | 1.15 | 0.620 | 1.01 | 0.85 | 1.20 | 0.859 | 0.94 | 0.78 | 1.13 | 0.374 |
|  |  |  |  |  |  |  |  |  |  |  |  |  |
| Ethnic group (White) |  |  |  |  |  |  |  |  |  |  |  |  |
| African/Caribbean | 0.86 | 0.72 | 1.03 | 0.028 | 1.09 | 0.92 | 1.30 | 0.191 | 1.17 | 0.97 | 1.41 | 0.034 |
| Asian | 0.87 | 0.75 | 1.00 | 0.012 | 0.97 | 0.84 | 1.13 | 0.607 | 1.02 | 0.86 | 1.20 | 0.777 |
| Other | 1.00 | 0.87 | 1.15 | 1.000 | 1.09 | 0.94 | 1.26 | 0.124 | 1.06 | 0.92 | 1.24 | 0.286 |
|  |  |  |  |  |  |  |  |  |  |  |  |  |
| Age | 1.00 | 1.00 | 1.01 | 0.001 | 1.00 | 1.00 | 1.00 | 0.008 | 1.01 | 1.00 | 1.01 | <0.001 |
|  |  |  |  |  |  |  |  |  |  |  |  |  |
| Sex (Female) |  |  |  |  |  |  |  |  |  |  |  |  |
| Male | 0.95 | 0.91 | 1.00 | 0.013 | 0.86 | 0.82 | 0.90 | <0.001 | 0.79 | 0.75 | 0.83 | <0.001 |
|  |  |  |  |  |  |  |  |  |  |  |  |  |
| Year of diagnosis (1998-2005) |  |  |  |  |  |  |  |  |  |  |  |  |
| 2006 to 2010 | 1.90 | 0.62 | 5.78 | 0.138 | 2.20 | 1.08 | 4.48 | 0.004 | 2.36 | 1.43 | 3.88 | <0.001 |
| 2011 to 2015 | 2.35 | 0.77 | 7.17 | 0.048 | 2.51 | 1.24 | 5.10 | 0.001 | 2.61 | 1.59 | 4.29 | <0.001 |
| 2016 to 2020 | 3.21 | 1.05 | 9.82 | 0.007 | 3.10 | 1.52 | 6.33 | <0.001 | 2.86 | 1.74 | 4.72 | <0.001 |
|  |  |  |  |  |  |  |  |  |  |  |  |  |
| Coronary heart disease | 1.07 | 0.92 | 1.23 | 0.247 | 1.22 | 1.05 | 1.42 | 0.001 | 1.40 | 1.17 | 1.68 | <0.001 |
| Stroke | 1.14 | 0.97 | 1.34 | 0.039 | 1.23 | 1.04 | 1.45 | 0.002 | 1.25 | 1.01 | 1.53 | 0.007 |
| Diabetes | 1.02 | 0.92 | 1.13 | 0.634 | 1.18 | 1.06 | 1.31 | <0.001 | 1.33 | 1.18 | 1.51 | <0.001 |
| Epilepsy | 1.18 | 0.96 | 1.44 | 0.042 | 1.23 | 0.98 | 1.53 | 0.018 | 1.23 | 0.96 | 1.58 | 0.034 |
| Hypothyroidism | 1.00 | 0.88 | 1.12 | 0.928 | 1.00 | 0.88 | 1.14 | 0.947 | 0.99 | 0.86 | 1.14 | 0.855 |
| Arthritis | 1.10 | 0.99 | 1.21 | 0.015 | 1.17 | 1.06 | 1.29 | <0.001 | 1.26 | 1.12 | 1.41 | <0.001 |
| Anxiety | 1.80 | 1.67 | 1.93 | <0.001 | 1.79 | 1.67 | 1.92 | <0.001 | 1.78 | 1.64 | 1.92 | <0.001 |
| Migraine | 1.14 | 1.05 | 1.24 | <0.001 | 1.27 | 1.16 | 1.39 | <0.001 | 1.34 | 1.22 | 1.48 | <0.001 |
| Cancer | 1.08 | 1.00 | 1.18 | 0.012 | 1.17 | 1.07 | 1.28 | <0.001 | 1.20 | 1.09 | 1.32 | <0.001 |
| Asthma | 1.18 | 1.12 | 1.25 | <0.001 | 1.38 | 1.29 | 1.47 | <0.001 | 1.52 | 1.42 | 1.63 | <0.001 |
| Renal failure | 1.31 | 0.89 | 1.95 | 0.074 | 1.69 | 1.05 | 2.74 | 0.005 | 1.93 | 1.09 | 3.42 | 0.003 |
| Liver failure | 1.15 | 0.91 | 1.45 | 0.118 | 1.40 | 1.10 | 1.79 | <0.001 | 1.41 | 1.07 | 1.86 | 0.001 |
| Osteoporosis | 1.07 | 0.82 | 1.39 | 0.515 | 1.13 | 0.86 | 1.47 | 0.250 | 1.15 | 0.85 | 1.55 | 0.245 |
| Suicidality | 1.19 | 1.04 | 1.35 | 0.001 | 1.23 | 1.08 | 1.42 | <0.001 | 1.24 | 1.07 | 1.43 | <0.001 |
| Antihypertensive | 1.05 | 0.96 | 1.15 | 0.147 | 1.09 | 1.00 | 1.19 | 0.008 | 1.09 | 0.98 | 1.21 | 0.039 |
| Aspirin | 1.07 | 0.94 | 1.22 | 0.157 | 1.08 | 0.94 | 1.23 | 0.159 | 1.13 | 0.97 | 1.32 | 0.039 |
| Anticoagulants | 1.24 | 0.98 | 1.57 | 0.019 | 1.33 | 1.05 | 1.69 | 0.002 | 1.71 | 1.27 | 2.31 | <0.001 |
| NSAIDs | 1.04 | 0.92 | 1.18 | 0.418 | 1.11 | 0.97 | 1.26 | 0.047 | 1.24 | 1.07 | 1.43 | <0.001 |
| Anticonvulsants | 1.18 | 0.94 | 1.48 | 0.064 | 1.38 | 1.08 | 1.75 | 0.001 | 1.47 | 1.11 | 1.97 | 0.001 |
| Hypnotics | 1.22 | 1.12 | 1.34 | <0.001 | 1.16 | 1.06 | 1.26 | <0.001 | 1.14 | 1.03 | 1.25 | 0.001 |
| Bisphosphonates | 1.19 | 0.77 | 1.84 | 0.314 | 1.27 | 0.80 | 2.00 | 0.180 | 1.09 | 0.64 | 1.86 | 0.683 |
| Contraceptives | 1.00 | 0.92 | 1.10 | 0.893 | 1.07 | 0.97 | 1.17 | 0.063 | 1.08 | 0.97 | 1.20 | 0.054 |
| *Constant* | 0.29 | 0.09 | 0.95 | 0.007 | 0.47 | 0.21 | 1.05 | 0.015 | 0.60 | 0.32 | 1.13 | 0.040 |

|  | **Safety, any adverse event *(FSA, N = 673,177)*** | | | | | | | | | | | |
| --- | --- | --- | --- | --- | --- | --- | --- | --- | --- | --- | --- | --- |
|  | **2 months** | | | | **6 months** | | | | **12 months** | | | |
|  | *OR* | *99% CI* | | *P* | *OR* | *99% CI* | | *P* | *OR* | *99% CI* | | *P* |
| ***Statins*** | 0.96 | 0.92 | 0.99 | 0.001 | 0.98 | 0.95 | 1.02 | 0.174 | 0.99 | 0.95 | 1.03 | 0.468 |
| Antidepressant category (SSRIs) |  |  |  |  |  |  |  |  |  |  |  |  |
| TCAs | 1.18 | 1.13 | 1.24 | <0.001 | 1.16 | 1.11 | 1.21 | <0.001 | 1.16 | 1.11 | 1.22 | <0.001 |
| MAOIs | 0.91 | 0.52 | 1.60 | 0.655 | 0.92 | 0.53 | 1.60 | 0.691 | 0.85 | 0.48 | 1.52 | 0.474 |
| Other antidepressants | 1.04 | 1.01 | 1.08 | 0.001 | 1.05 | 1.01 | 1.09 | <0.001 | 1.04 | 1.00 | 1.07 | 0.015 |
|  |  |  |  |  |  |  |  |  |  |  |  |  |
| PHQ-9 baseline | 1.00 | 1.00 | 1.00 | 0.213 | 1.00 | 1.00 | 1.00 | 0.855 | 1.00 | 1.00 | 1.00 | 0.786 |
| BMI | 1.00 | 1.00 | 1.00 | 0.278 | 1.00 | 1.00 | 1.00 | 0.018 | 1.00 | 1.00 | 1.00 | <0.001 |
|  |  |  |  |  |  |  |  |  |  |  |  |  |
| Read codes for depression (Major depression) |  |  |  |  |  |  |  |  |  |  |  |  |
| Minor depression | 1.70 | 1.63 | 1.77 | <0.001 | 1.66 | 1.59 | 1.73 | <0.001 | 1.63 | 1.57 | 1.70 | <0.001 |
| Other | 0.52 | 0.47 | 0.56 | <0.001 | 0.61 | 0.57 | 0.65 | <0.001 | 0.65 | 0.60 | 0.70 | <0.001 |
|  |  |  |  |  |  |  |  |  |  |  |  |  |
| Townsend deprivation score in fifths (1, least deprived) |  |  |  |  |  |  |  |  |  |  |  |  |
| 2 | 1.00 | 0.97 | 1.04 | 0.902 | 1.02 | 0.99 | 1.06 | 0.124 | 1.02 | 0.99 | 1.06 | 0.117 |
| 3 | 1.00 | 0.96 | 1.04 | 0.896 | 1.04 | 1.00 | 1.09 | 0.014 | 1.04 | 0.99 | 1.09 | 0.030 |
| 4 | 0.98 | 0.93 | 1.03 | 0.313 | 1.03 | 0.98 | 1.09 | 0.129 | 1.03 | 0.97 | 1.09 | 0.287 |
| 5, most deprived | 1.06 | 0.99 | 1.13 | 0.020 | 1.12 | 1.04 | 1.20 | <0.001 | 1.09 | 1.01 | 1.17 | 0.002 |
|  |  |  |  |  |  |  |  |  |  |  |  |  |
| Region of England (East Midlands) |  |  |  |  |  |  |  |  |  |  |  |  |
| East of England | 0.81 | 0.60 | 1.11 | 0.091 | 0.82 | 0.60 | 1.12 | 0.104 | 0.84 | 0.62 | 1.14 | 0.137 |
| London | 0.79 | 0.66 | 0.94 | 0.001 | 0.83 | 0.69 | 1.00 | 0.009 | 0.85 | 0.70 | 1.02 | 0.024 |
| North East | 0.94 | 0.71 | 1.25 | 0.588 | 0.96 | 0.72 | 1.28 | 0.696 | 0.98 | 0.73 | 1.33 | 0.879 |
| North West | 0.91 | 0.75 | 1.09 | 0.176 | 0.95 | 0.79 | 1.16 | 0.531 | 0.99 | 0.81 | 1.21 | 0.879 |
| South Central | 0.72 | 0.58 | 0.89 | <0.001 | 0.75 | 0.60 | 0.93 | 0.001 | 0.77 | 0.62 | 0.97 | 0.003 |
| South East | 0.76 | 0.60 | 0.95 | 0.002 | 0.79 | 0.63 | 1.00 | 0.011 | 0.81 | 0.64 | 1.03 | 0.024 |
| South West | 0.93 | 0.76 | 1.15 | 0.379 | 0.96 | 0.78 | 1.19 | 0.637 | 0.98 | 0.79 | 1.22 | 0.817 |
| West Midlands | 0.75 | 0.61 | 0.91 | <0.001 | 0.80 | 0.65 | 0.98 | 0.004 | 0.84 | 0.68 | 1.04 | 0.032 |
| Yorkshire & Humber | 0.92 | 0.72 | 1.18 | 0.389 | 0.92 | 0.71 | 1.19 | 0.394 | 0.93 | 0.71 | 1.21 | 0.449 |
|  |  |  |  |  |  |  |  |  |  |  |  |  |
| Smoking status (Non-smoker) |  |  |  |  |  |  |  |  |  |  |  |  |
| Ex-smoker | 1.02 | 0.99 | 1.04 | 0.083 | 1.04 | 1.02 | 1.07 | <0.001 | 1.05 | 1.02 | 1.08 | <0.001 |
| Light-smoker | 0.94 | 0.92 | 0.96 | <0.001 | 0.97 | 0.95 | 0.99 | <0.001 | 0.98 | 0.96 | 1.00 | 0.029 |
| Moderate-smoker | 0.91 | 0.86 | 0.97 | <0.001 | 0.94 | 0.89 | 1.01 | 0.020 | 0.96 | 0.90 | 1.03 | 0.133 |
| Heavy-smoker | 0.96 | 0.87 | 1.05 | 0.266 | 0.97 | 0.88 | 1.06 | 0.359 | 0.96 | 0.87 | 1.05 | 0.244 |
|  |  |  |  |  |  |  |  |  |  |  |  |  |
| Alcohol use (Non-drinker/trivial) |  |  |  |  |  |  |  |  |  |  |  |  |
| Light | 0.99 | 0.98 | 1.01 | 0.423 | 0.99 | 0.97 | 1.01 | 0.173 | 0.98 | 0.96 | 1.00 | 0.031 |
| Medium | 1.01 | 0.98 | 1.04 | 0.414 | 1.01 | 0.98 | 1.04 | 0.232 | 1.00 | 0.97 | 1.04 | 0.725 |
| Heavy | 1.00 | 0.92 | 1.08 | 0.928 | 1.01 | 0.94 | 1.08 | 0.733 | 1.01 | 0.93 | 1.09 | 0.760 |
| Very heavy | 1.00 | 0.94 | 1.07 | 0.988 | 1.00 | 0.94 | 1.07 | 0.968 | 0.99 | 0.92 | 1.06 | 0.693 |
|  |  |  |  |  |  |  |  |  |  |  |  |  |
| Ethnic group (White) |  |  |  |  |  |  |  |  |  |  |  |  |
| African/Caribbean | 0.91 | 0.87 | 0.95 | <0.001 | 0.94 | 0.90 | 0.98 | <0.001 | 0.93 | 0.89 | 0.98 | <0.001 |
| Asian | 0.95 | 0.90 | 1.01 | 0.033 | 1.08 | 1.01 | 1.15 | 0.003 | 1.14 | 1.06 | 1.22 | <0.001 |
| Other | 0.97 | 0.92 | 1.01 | 0.057 | 1.03 | 0.98 | 1.08 | 0.181 | 1.03 | 0.98 | 1.09 | 0.109 |
|  |  |  |  |  |  |  |  |  |  |  |  |  |
| Age | 1.00 | 1.00 | 1.01 | <0.001 | 1.00 | 1.00 | 1.01 | <0.001 | 1.01 | 1.01 | 1.01 | <0.001 |
|  |  |  |  |  |  |  |  |  |  |  |  |  |
| Sex (Female) |  |  |  |  |  |  |  |  |  |  |  |  |
| Male | 0.93 | 0.92 | 0.94 | <0.001 | 0.83 | 0.81 | 0.84 | <0.001 | 0.76 | 0.75 | 0.78 | <0.001 |
|  |  |  |  |  |  |  |  |  |  |  |  |  |
| Year of diagnosis (1998-2005) |  |  |  |  |  |  |  |  |  |  |  |  |
| 2006 to 2010 | 1.10 | 1.04 | 1.16 | <0.001 | 1.07 | 1.01 | 1.13 | 0.002 | 1.04 | 0.98 | 1.10 | 0.102 |
| 2011 to 2015 | 1.26 | 1.18 | 1.35 | <0.001 | 1.20 | 1.13 | 1.28 | <0.001 | 1.15 | 1.07 | 1.22 | <0.001 |
| 2016 to 2020 | 1.40 | 1.31 | 1.51 | <0.001 | 1.22 | 1.14 | 1.31 | <0.001 | 1.06 | 0.99 | 1.13 | 0.043 |
|  |  |  |  |  |  |  |  |  |  |  |  |  |
| Coronary heart disease | 1.09 | 1.04 | 1.14 | <0.001 | 1.17 | 1.11 | 1.23 | <0.001 | 1.29 | 1.22 | 1.37 | <0.001 |
| Stroke | 1.08 | 1.03 | 1.13 | <0.001 | 1.18 | 1.12 | 1.24 | <0.001 | 1.24 | 1.16 | 1.31 | <0.001 |
| Diabetes | 1.04 | 1.00 | 1.08 | 0.004 | 1.15 | 1.11 | 1.19 | <0.001 | 1.27 | 1.22 | 1.33 | <0.001 |
| Epilepsy | 1.00 | 0.94 | 1.07 | 0.993 | 1.12 | 1.04 | 1.19 | <0.001 | 1.19 | 1.10 | 1.28 | <0.001 |
| Hypothyroidism | 1.01 | 0.98 | 1.05 | 0.349 | 1.04 | 1.00 | 1.09 | 0.009 | 1.08 | 1.03 | 1.13 | <0.001 |
| Arthritis | 1.07 | 1.03 | 1.10 | <0.001 | 1.17 | 1.13 | 1.21 | <0.001 | 1.25 | 1.20 | 1.30 | <0.001 |
| Anxiety | 1.57 | 1.51 | 1.62 | <0.001 | 1.61 | 1.56 | 1.67 | <0.001 | 1.63 | 1.57 | 1.69 | <0.001 |
| Migraine | 1.11 | 1.07 | 1.14 | <0.001 | 1.19 | 1.16 | 1.23 | <0.001 | 1.28 | 1.24 | 1.33 | <0.001 |
| Cancer | 1.10 | 1.07 | 1.13 | <0.001 | 1.17 | 1.14 | 1.21 | <0.001 | 1.26 | 1.22 | 1.31 | <0.001 |
| Asthma | 1.21 | 1.18 | 1.23 | <0.001 | 1.38 | 1.35 | 1.41 | <0.001 | 1.52 | 1.48 | 1.56 | <0.001 |
| Renal failure | 1.30 | 1.15 | 1.47 | <0.001 | 1.39 | 1.21 | 1.60 | <0.001 | 1.64 | 1.38 | 1.95 | <0.001 |
| Liver failure | 1.05 | 0.98 | 1.14 | 0.073 | 1.22 | 1.12 | 1.33 | <0.001 | 1.27 | 1.16 | 1.40 | <0.001 |
| Osteoporosis | 1.09 | 1.02 | 1.16 | 0.001 | 1.18 | 1.10 | 1.26 | <0.001 | 1.27 | 1.17 | 1.39 | <0.001 |
| Suicidality | 1.10 | 1.05 | 1.15 | <0.001 | 1.16 | 1.10 | 1.22 | <0.001 | 1.15 | 1.09 | 1.22 | <0.001 |
| Antihypertensive | 0.99 | 0.96 | 1.03 | 0.633 | 1.04 | 1.01 | 1.08 | 0.002 | 1.05 | 1.01 | 1.10 | <0.001 |
| Aspirin | 1.07 | 1.02 | 1.11 | <0.001 | 1.12 | 1.07 | 1.17 | <0.001 | 1.18 | 1.12 | 1.24 | <0.001 |
| Anticoagulants | 1.27 | 1.19 | 1.36 | <0.001 | 1.44 | 1.34 | 1.56 | <0.001 | 1.63 | 1.49 | 1.79 | <0.001 |
| NSAIDs | 1.11 | 1.07 | 1.15 | <0.001 | 1.16 | 1.12 | 1.20 | <0.001 | 1.20 | 1.15 | 1.25 | <0.001 |
| Anticonvulsants | 1.28 | 1.21 | 1.36 | <0.001 | 1.39 | 1.30 | 1.48 | <0.001 | 1.49 | 1.38 | 1.60 | <0.001 |
| Hypnotics | 1.23 | 1.20 | 1.27 | <0.001 | 1.18 | 1.15 | 1.22 | <0.001 | 1.15 | 1.12 | 1.19 | <0.001 |
| Bisphosphonates | 1.12 | 0.99 | 1.26 | 0.017 | 1.15 | 1.01 | 1.31 | 0.005 | 1.13 | 0.97 | 1.32 | 0.040 |
| Contraceptives | 1.03 | 1.00 | 1.06 | 0.029 | 1.08 | 1.04 | 1.11 | <0.001 | 1.11 | 1.07 | 1.15 | <0.001 |
| *Constant* | 0.55 | 0.46 | 0.66 | <0.001 | 0.96 | 0.79 | 1.16 | 0.548 | 1.42 | 1.16 | 1.74 | <0.001 |

### **Table A10d.** Regression analyses for safety (self-harm) at 2 months, 6 months, and 12 months; complete case analysis (CCA) and full set analysis (FSA)

|  | **Safety, self-harm *(CCA, N= 63,092)*** | | | | | | | | | | | |
| --- | --- | --- | --- | --- | --- | --- | --- | --- | --- | --- | --- | --- |
|  | **2 months** | | | | **6 months** | | | | **12 months** | | | |
|  | *OR* | *99% CI* | | *P* | *OR* | *99% CI* | | *P* | *OR* | *99% CI* | | *P* |
| ***Statins*** | 0.14 | 0.01 | 1.46 | 0.031 | 0.22 | 0.04 | 1.16 | 0.019 | 0.20 | 0.06 | 0.72 | 0.001 |
| Antidepressant category (SSRIs) |  |  |  |  |  |  |  |  |  |  |  |  |
| TCAs | 1.14 | 0.26 | 5.12 | 0.816 | 1.19 | 0.41 | 3.46 | 0.682 | 0.96 | 0.35 | 2.59 | 0.912 |
| MAOIs |  |  |  |  |  |  |  |  |  |  |  |  |
| Other antidepressants | 1.18 | 0.54 | 2.55 | 0.585 | 1.23 | 0.69 | 2.17 | 0.360 | 1.18 | 0.72 | 1.93 | 0.391 |
|  |  |  |  |  |  |  |  |  |  |  |  |  |
| PHQ-9 baseline | 1.08 | 1.03 | 1.13 | <0.001 | 1.07 | 1.03 | 1.11 | <0.001 | 1.07 | 1.03 | 1.10 | <0.001 |
| BMI | 0.97 | 0.92 | 1.02 | 0.144 | 0.99 | 0.96 | 1.02 | 0.285 | 0.97 | 0.95 | 1.00 | 0.015 |
|  |  |  |  |  |  |  |  |  |  |  |  |  |
| Read codes for depression (Major depression) |  |  |  |  |  |  |  |  |  |  |  |  |
| Minor depression | 0.81 | 0.50 | 1.32 | 0.260 | 0.91 | 0.65 | 1.27 | 0.475 | 0.91 | 0.69 | 1.21 | 0.408 |
| Other | 2.14 | 0.63 | 7.28 | 0.110 | 1.09 | 0.32 | 3.74 | 0.853 | 0.90 | 0.29 | 2.76 | 0.811 |
|  |  |  |  |  |  |  |  |  |  |  |  |  |
| Townsend deprivation score in fifths (1, least deprived) |  |  |  |  |  |  |  |  |  |  |  |  |
| 2 | 0.91 | 0.47 | 1.75 | 0.698 | 1.01 | 0.62 | 1.65 | 0.937 | 1.12 | 0.73 | 1.72 | 0.483 |
| 3 | 0.77 | 0.38 | 1.55 | 0.339 | 0.90 | 0.55 | 1.48 | 0.593 | 1.09 | 0.71 | 1.68 | 0.599 |
| 4 | 0.93 | 0.47 | 1.85 | 0.800 | 1.11 | 0.66 | 1.86 | 0.601 | 1.44 | 0.92 | 2.25 | 0.038 |
| 5, most deprived | 0.80 | 0.39 | 1.65 | 0.435 | 0.88 | 0.51 | 1.52 | 0.550 | 1.09 | 0.67 | 1.76 | 0.650 |
|  |  |  |  |  |  |  |  |  |  |  |  |  |
| Region of England (East Midlands) |  |  |  |  |  |  |  |  |  |  |  |  |
| East of England | 0.32 | 0.04 | 2.28 | 0.135 | 0.38 | 0.10 | 1.52 | 0.072 | 0.31 | 0.08 | 1.18 | 0.024 |
| London | 0.55 | 0.20 | 1.57 | 0.144 | 0.64 | 0.30 | 1.33 | 0.114 | 0.71 | 0.37 | 1.36 | 0.172 |
| North East | 0.31 | 0.07 | 1.48 | 0.054 | 0.66 | 0.25 | 1.73 | 0.268 | 0.85 | 0.39 | 1.87 | 0.607 |
| North West | 0.66 | 0.22 | 1.93 | 0.316 | 0.63 | 0.29 | 1.39 | 0.130 | 0.73 | 0.37 | 1.44 | 0.230 |
| South Central | 0.89 | 0.30 | 2.61 | 0.785 | 1.09 | 0.52 | 2.29 | 0.768 | 1.26 | 0.66 | 2.40 | 0.354 |
| South East | 0.82 | 0.28 | 2.38 | 0.634 | 0.81 | 0.37 | 1.76 | 0.476 | 0.87 | 0.43 | 1.79 | 0.630 |
| South West | 0.70 | 0.21 | 2.31 | 0.448 | 0.81 | 0.34 | 1.94 | 0.540 | 0.89 | 0.42 | 1.92 | 0.708 |
| West Midlands | 0.39 | 0.11 | 1.37 | 0.055 | 0.56 | 0.23 | 1.34 | 0.084 | 0.73 | 0.35 | 1.52 | 0.266 |
| Yorkshire & Humber | 0.95 | 0.31 | 2.88 | 0.902 | 0.76 | 0.34 | 1.71 | 0.388 | 0.86 | 0.42 | 1.76 | 0.584 |
|  |  |  |  |  |  |  |  |  |  |  |  |  |
| Smoking status (Non-smoker) |  |  |  |  |  |  |  |  |  |  |  |  |
| Ex-smoker | 1.02 | 0.58 | 1.81 | 0.918 | 0.98 | 0.63 | 1.54 | 0.930 | 1.05 | 0.73 | 1.52 | 0.708 |
| Light-smoker | 1.21 | 0.73 | 2.01 | 0.335 | 1.44 | 1.03 | 2.01 | 0.005 | 1.43 | 1.07 | 1.91 | 0.002 |
| Moderate-smoker | 1.69 | 0.50 | 5.67 | 0.265 | 1.48 | 0.58 | 3.80 | 0.279 | 1.84 | 0.90 | 3.79 | 0.029 |
| Heavy-smoker | 2.95 | 0.71 | 12.20 | 0.050 | 2.16 | 0.62 | 7.47 | 0.111 | 1.84 | 0.60 | 5.69 | 0.162 |
|  |  |  |  |  |  |  |  |  |  |  |  |  |
| Alcohol use (Non-drinker/trivial) |  |  |  |  |  |  |  |  |  |  |  |  |
| Light | 1.56 | 0.98 | 2.48 | 0.013 | 1.51 | 1.08 | 2.12 | 0.002 | 1.46 | 1.08 | 1.97 | 0.001 |
| Medium | 2.81 | 1.66 | 4.77 | <0.001 | 2.14 | 1.38 | 3.30 | <0.001 | 2.49 | 1.72 | 3.61 | <0.001 |
| Heavy | 1.56 | 0.33 | 7.48 | 0.463 | 1.87 | 0.67 | 5.23 | 0.119 | 1.93 | 0.82 | 4.51 | 0.047 |
| Very heavy | 2.28 | 0.77 | 6.76 | 0.050 | 2.28 | 1.06 | 4.90 | 0.006 | 1.88 | 0.94 | 3.77 | 0.020 |
|  |  |  |  |  |  |  |  |  |  |  |  |  |
| Ethnic group (White) |  |  |  |  |  |  |  |  |  |  |  |  |
| African/Caribbean | 2.04 | 0.71 | 5.90 | 0.083 | 1.67 | 0.77 | 3.64 | 0.090 | 1.18 | 0.53 | 2.62 | 0.592 |
| Asian | 1.22 | 0.41 | 3.58 | 0.639 | 1.00 | 0.43 | 2.29 | 0.990 | 1.17 | 0.55 | 2.48 | 0.589 |
| Other | 0.71 | 0.19 | 2.60 | 0.494 | 0.85 | 0.37 | 1.97 | 0.614 | 0.93 | 0.47 | 1.84 | 0.774 |
|  |  |  |  |  |  |  |  |  |  |  |  |  |
| Age | 0.97 | 0.95 | 0.99 | 0.001 | 0.96 | 0.94 | 0.98 | <0.001 | 0.97 | 0.95 | 0.98 | <0.001 |
|  |  |  |  |  |  |  |  |  |  |  |  |  |
| Sex (Female) |  |  |  |  |  |  |  |  |  |  |  |  |
| Male | 1.29 | 0.83 | 2.01 | 0.135 | 1.31 | 0.94 | 1.83 | 0.035 | 1.10 | 0.83 | 1.45 | 0.401 |
|  |  |  |  |  |  |  |  |  |  |  |  |  |
| Year of diagnosis (1998-2005) |  |  |  |  |  |  |  |  |  |  |  |  |
| 2006 to 2010 | 0.54 | 0.32 | 0.92 | 0.003 | 0.66 | 0.45 | 0.97 | 0.005 | 0.77 | 0.55 | 1.06 | 0.037 |
| 2011 to 2015 | 0.65 | 0.40 | 1.07 | 0.026 | 0.67 | 0.47 | 0.98 | 0.006 | 0.74 | 0.54 | 1.02 | 0.014 |
| 2016 to 2020 | 1.00 |  |  |  | 1.00 |  |  |  | 1.00 |  |  |  |
|  |  |  |  |  |  |  |  |  |  |  |  |  |
| Coronary heart disease | 7.08 | 1.65 | 30.40 | 0.001 | 3.43 | 0.70 | 16.91 | 0.046 | 3.03 | 0.70 | 13.05 | 0.051 |
| Stroke | 1.56 | 0.20 | 12.20 | 0.575 | 0.95 | 0.14 | 6.23 | 0.940 | 1.44 | 0.40 | 5.27 | 0.466 |
| Diabetes | 0.46 | 0.07 | 3.04 | 0.293 | 0.62 | 0.18 | 2.15 | 0.319 | 0.77 | 0.30 | 2.01 | 0.490 |
| Epilepsy | 2.37 | 0.62 | 9.08 | 0.099 | 1.91 | 0.66 | 5.49 | 0.114 | 1.36 | 0.49 | 3.81 | 0.436 |
| Hypothyroidism | 1.50 | 0.37 | 6.02 | 0.453 | 1.43 | 0.51 | 4.07 | 0.374 | 1.11 | 0.43 | 2.90 | 0.771 |
| Arthritis | 1.49 | 0.48 | 4.58 | 0.365 | 1.37 | 0.56 | 3.35 | 0.365 | 0.94 | 0.39 | 2.24 | 0.846 |
| Anxiety | 1.05 | 0.60 | 1.81 | 0.832 | 1.01 | 0.67 | 1.52 | 0.951 | 0.99 | 0.69 | 1.41 | 0.914 |
| Migraine | 0.87 | 0.39 | 1.96 | 0.658 | 1.41 | 0.81 | 2.46 | 0.110 | 1.26 | 0.78 | 2.05 | 0.216 |
| Cancer | 0.77 | 0.28 | 2.11 | 0.508 | 1.13 | 0.57 | 2.21 | 0.650 | 1.04 | 0.59 | 1.84 | 0.854 |
| Asthma | 1.14 | 0.68 | 1.90 | 0.521 | 1.02 | 0.70 | 1.47 | 0.910 | 1.14 | 0.84 | 1.53 | 0.269 |
| Renal failure | 6.51 | 0.75 | 56.74 | 0.026 | 6.72 | 1.33 | 33.89 | 0.002 | 4.30 | 0.84 | 22.13 | 0.022 |
| Liver failure | 0.85 | 0.07 | 10.09 | 0.864 | 1.81 | 0.48 | 6.82 | 0.251 | 1.65 | 0.50 | 5.45 | 0.281 |
| Osteoporosis | 1.00 |  |  |  | 1.00 |  |  |  | 1.00 |  |  |  |
| Suicidality | 6.03 | 3.51 | 10.37 | <0.001 | 5.80 | 3.88 | 8.65 | <0.001 | 5.93 | 4.25 | 8.28 | <0.001 |
| Antihypertensive | 1.06 | 0.37 | 2.98 | 0.891 | 0.90 | 0.34 | 2.34 | 0.767 | 1.15 | 0.53 | 2.48 | 0.638 |
| Aspirin | 0.33 | 0.02 | 6.57 | 0.341 | 1.55 | 0.26 | 9.15 | 0.522 | 1.37 | 0.34 | 5.53 | 0.561 |
| Anticoagulants | 2.60 | 0.41 | 16.38 | 0.180 | 2.18 | 0.38 | 12.62 | 0.255 | 1.44 | 0.24 | 8.50 | 0.594 |
| NSAIDs | 1.55 | 0.52 | 4.66 | 0.302 | 1.35 | 0.58 | 3.13 | 0.359 | 1.08 | 0.49 | 2.38 | 0.814 |
| Anticonvulsants | 0.39 | 0.03 | 5.28 | 0.356 | 0.70 | 0.14 | 3.59 | 0.572 | 0.61 | 0.13 | 2.96 | 0.421 |
| Hypnotics | 2.64 | 1.52 | 4.59 | <0.001 | 1.99 | 1.28 | 3.07 | <0.001 | 1.96 | 1.37 | 2.81 | <0.001 |
| Bisphosphonates | 1.00 |  |  |  | 1.00 |  |  |  | 1.00 |  |  |  |
| Contraceptives | 1.45 | 0.68 | 3.10 | 0.202 | 1.11 | 0.62 | 1.98 | 0.647 | 1.07 | 0.66 | 1.74 | 0.707 |
| *Constant* | 0.00 | 0.00 | 0.03 | <0.001 | 0.01 | 0.00 | 0.03 | <0.001 | 0.01 | 0.00 | 0.03 | <0.001 |

|  | **Safety, self-harm *(FSA, N = 673,177)*** | | | | | | | | | | | |
| --- | --- | --- | --- | --- | --- | --- | --- | --- | --- | --- | --- | --- |
|  | **2 months** | | | | **6 months** | | | | **12 months** | | | |
|  | *OR* | *99% CI* | | *P* | *OR* | *99% CI* | | *P* | *OR* | *99% CI* | | *P* |
| ***Statins*** | 0.70 | 0.44 | 1.12 | 0.049 | 0.73 | 0.51 | 1.05 | 0.026 | 0.81 | 0.60 | 1.09 | 0.068 |
| Antidepressant category (SSRIs) |  |  |  |  |  |  |  |  |  |  |  |  |
| TCAs | 1.20 | 0.94 | 1.53 | 0.055 | 1.13 | 0.93 | 1.36 | 0.104 | 1.08 | 0.92 | 1.28 | 0.220 |
| MAOIs | 1.00 |  |  |  | 2.43 | 0.17 | 35.18 | 0.392 | 1.82 | 0.13 | 26.09 | 0.564 |
| Other antidepressants | 1.46 | 1.21 | 1.77 | <0.001 | 1.49 | 1.29 | 1.73 | <0.001 | 1.48 | 1.31 | 1.68 | <0.001 |
|  |  |  |  |  |  |  |  |  |  |  |  |  |
| PHQ-9 baseline | 1.01 | 1.00 | 1.03 | 0.048 | 1.01 | 1.00 | 1.03 | 0.003 | 1.01 | 1.00 | 1.03 | 0.002 |
| BMI | 0.99 | 0.98 | 1.00 | 0.017 | 0.99 | 0.98 | 1.00 | 0.024 | 0.99 | 0.99 | 1.00 | 0.021 |
|  |  |  |  |  |  |  |  |  |  |  |  |  |
| Read codes for depression (Major depression) |  |  |  |  |  |  |  |  |  |  |  |  |
| Minor depression | 0.94 | 0.80 | 1.10 | 0.298 | 0.91 | 0.81 | 1.02 | 0.031 | 0.92 | 0.83 | 1.01 | 0.023 |
| Other | 0.75 | 0.52 | 1.09 | 0.050 | 0.74 | 0.56 | 0.97 | 0.004 | 0.79 | 0.63 | 0.99 | 0.008 |
|  |  |  |  |  |  |  |  |  |  |  |  |  |
| Townsend deprivation score in fifths (1, least deprived) |  |  |  |  |  |  |  |  |  |  |  |  |
| 2 | 1.03 | 0.84 | 1.27 | 0.673 | 1.04 | 0.89 | 1.21 | 0.515 | 1.06 | 0.93 | 1.22 | 0.238 |
| 3 | 1.08 | 0.89 | 1.32 | 0.314 | 1.16 | 1.00 | 1.36 | 0.012 | 1.21 | 1.06 | 1.39 | <0.001 |
| 4 | 1.12 | 0.91 | 1.38 | 0.164 | 1.28 | 1.09 | 1.50 | <0.001 | 1.32 | 1.14 | 1.52 | <0.001 |
| 5, most deprived | 1.28 | 1.00 | 1.63 | 0.009 | 1.34 | 1.11 | 1.60 | <0.001 | 1.45 | 1.24 | 1.69 | <0.001 |
|  |  |  |  |  |  |  |  |  |  |  |  |  |
| Region of England (East Midlands) |  |  |  |  |  |  |  |  |  |  |  |  |
| East of England | 0.92 | 0.57 | 1.47 | 0.638 | 0.88 | 0.61 | 1.27 | 0.366 | 0.80 | 0.57 | 1.11 | 0.081 |
| London | 0.61 | 0.41 | 0.91 | 0.001 | 0.59 | 0.45 | 0.77 | <0.001 | 0.59 | 0.48 | 0.73 | <0.001 |
| North East | 0.92 | 0.59 | 1.43 | 0.615 | 0.95 | 0.70 | 1.28 | 0.637 | 1.01 | 0.77 | 1.32 | 0.945 |
| North West | 0.72 | 0.50 | 1.03 | 0.018 | 0.74 | 0.57 | 0.96 | 0.003 | 0.75 | 0.61 | 0.92 | <0.001 |
| South Central | 0.82 | 0.54 | 1.24 | 0.215 | 0.81 | 0.61 | 1.09 | 0.065 | 0.86 | 0.69 | 1.09 | 0.100 |
| South East | 1.01 | 0.67 | 1.55 | 0.929 | 0.90 | 0.65 | 1.23 | 0.374 | 0.91 | 0.70 | 1.19 | 0.364 |
| South West | 0.89 | 0.58 | 1.36 | 0.487 | 0.88 | 0.66 | 1.18 | 0.273 | 0.91 | 0.71 | 1.15 | 0.296 |
| West Midlands | 0.61 | 0.41 | 0.91 | 0.001 | 0.58 | 0.43 | 0.78 | <0.001 | 0.63 | 0.50 | 0.80 | <0.001 |
| Yorkshire & Humber | 0.78 | 0.49 | 1.22 | 0.153 | 0.80 | 0.56 | 1.13 | 0.096 | 0.84 | 0.63 | 1.14 | 0.143 |
|  |  |  |  |  |  |  |  |  |  |  |  |  |
| Smoking status (Non-smoker) |  |  |  |  |  |  |  |  |  |  |  |  |
| Ex-smoker | 0.90 | 0.75 | 1.08 | 0.147 | 0.88 | 0.76 | 1.02 | 0.027 | 0.87 | 0.76 | 0.98 | 0.003 |
| Light-smoker | 1.16 | 1.01 | 1.33 | 0.004 | 1.22 | 1.10 | 1.35 | <0.001 | 1.28 | 1.17 | 1.40 | <0.001 |
| Moderate-smoker | 1.43 | 1.03 | 1.98 | 0.004 | 1.45 | 1.13 | 1.88 | <0.001 | 1.47 | 1.18 | 1.83 | <0.001 |
| Heavy-smoker | 1.29 | 0.76 | 2.19 | 0.221 | 1.37 | 0.89 | 2.09 | 0.060 | 1.49 | 1.05 | 2.11 | 0.003 |
|  |  |  |  |  |  |  |  |  |  |  |  |  |
| Alcohol use (Non-drinker/trivial) |  |  |  |  |  |  |  |  |  |  |  |  |
| Light | 1.02 | 0.86 | 1.20 | 0.805 | 1.05 | 0.92 | 1.19 | 0.321 | 1.07 | 0.97 | 1.18 | 0.084 |
| Medium | 1.20 | 0.96 | 1.51 | 0.034 | 1.21 | 1.03 | 1.43 | 0.003 | 1.20 | 1.04 | 1.39 | 0.001 |
| Heavy | 1.50 | 0.97 | 2.32 | 0.016 | 1.47 | 1.06 | 2.04 | 0.003 | 1.58 | 1.21 | 2.07 | <0.001 |
| Very heavy | 1.36 | 0.91 | 2.04 | 0.050 | 1.49 | 1.06 | 2.07 | 0.002 | 1.53 | 1.16 | 2.00 | <0.001 |
|  |  |  |  |  |  |  |  |  |  |  |  |  |
| Ethnic group (White) |  |  |  |  |  |  |  |  |  |  |  |  |
| African/Caribbean | 1.02 | 0.73 | 1.40 | 0.901 | 1.00 | 0.79 | 1.28 | 0.958 | 0.94 | 0.74 | 1.19 | 0.473 |
| Asian | 0.99 | 0.75 | 1.29 | 0.885 | 0.88 | 0.71 | 1.09 | 0.129 | 0.94 | 0.78 | 1.13 | 0.376 |
| Other | 0.89 | 0.65 | 1.22 | 0.334 | 1.04 | 0.82 | 1.32 | 0.650 | 1.03 | 0.84 | 1.26 | 0.750 |
|  |  |  |  |  |  |  |  |  |  |  |  |  |
| Age | 0.97 | 0.96 | 0.97 | <0.001 | 0.96 | 0.96 | 0.97 | <0.001 | 0.96 | 0.96 | 0.97 | <0.001 |
|  |  |  |  |  |  |  |  |  |  |  |  |  |
| Sex (Female) |  |  |  |  |  |  |  |  |  |  |  |  |
| Male | 1.30 | 1.15 | 1.46 | <0.001 | 1.18 | 1.08 | 1.30 | <0.001 | 1.12 | 1.03 | 1.22 | <0.001 |
|  |  |  |  |  |  |  |  |  |  |  |  |  |
| Year of diagnosis (1998-2005) |  |  |  |  |  |  |  |  |  |  |  |  |
| 2006 to 2010 | 0.99 | 0.79 | 1.23 | 0.873 | 1.02 | 0.87 | 1.19 | 0.793 | 1.00 | 0.87 | 1.15 | 0.970 |
| 2011 to 2015 | 1.06 | 0.85 | 1.31 | 0.525 | 1.08 | 0.90 | 1.28 | 0.282 | 1.07 | 0.91 | 1.25 | 0.285 |
| 2016 to 2020 | 1.28 | 1.01 | 1.62 | 0.008 | 1.32 | 1.10 | 1.59 | <0.001 | 1.27 | 1.09 | 1.49 | <0.001 |
|  |  |  |  |  |  |  |  |  |  |  |  |  |
| Coronary heart disease | 1.13 | 0.58 | 2.18 | 0.640 | 1.33 | 0.81 | 2.18 | 0.134 | 1.35 | 0.88 | 2.07 | 0.068 |
| Stroke | 0.89 | 0.48 | 1.65 | 0.620 | 1.03 | 0.63 | 1.66 | 0.885 | 1.01 | 0.66 | 1.55 | 0.939 |
| Diabetes | 1.19 | 0.80 | 1.75 | 0.261 | 1.15 | 0.84 | 1.57 | 0.261 | 1.12 | 0.85 | 1.46 | 0.291 |
| Epilepsy | 1.22 | 0.79 | 1.90 | 0.245 | 1.10 | 0.77 | 1.57 | 0.485 | 1.11 | 0.81 | 1.53 | 0.380 |
| Hypothyroidism | 0.87 | 0.55 | 1.39 | 0.447 | 0.88 | 0.61 | 1.26 | 0.362 | 0.88 | 0.64 | 1.21 | 0.292 |
| Arthritis | 1.55 | 1.11 | 2.17 | 0.001 | 1.36 | 1.03 | 1.80 | 0.004 | 1.23 | 0.97 | 1.58 | 0.026 |
| Anxiety | 1.07 | 0.90 | 1.27 | 0.345 | 1.02 | 0.89 | 1.17 | 0.700 | 1.02 | 0.92 | 1.14 | 0.581 |
| Migraine | 0.78 | 0.59 | 1.01 | 0.014 | 0.88 | 0.73 | 1.07 | 0.084 | 0.90 | 0.76 | 1.06 | 0.092 |
| Cancer | 1.03 | 0.78 | 1.36 | 0.818 | 1.03 | 0.83 | 1.28 | 0.713 | 0.99 | 0.82 | 1.20 | 0.943 |
| Asthma | 1.01 | 0.87 | 1.17 | 0.836 | 1.06 | 0.95 | 1.17 | 0.192 | 1.11 | 1.02 | 1.22 | 0.002 |
| Renal failure | 0.92 | 0.25 | 3.46 | 0.875 | 0.72 | 0.22 | 2.33 | 0.467 | 0.77 | 0.29 | 2.08 | 0.506 |
| Liver failure | 0.97 | 0.49 | 1.90 | 0.897 | 1.25 | 0.79 | 1.98 | 0.219 | 1.18 | 0.78 | 1.78 | 0.315 |
| Osteoporosis | 1.13 | 0.48 | 2.67 | 0.704 | 1.10 | 0.53 | 2.28 | 0.736 | 1.05 | 0.55 | 2.01 | 0.850 |
| Suicidality | 7.10 | 5.92 | 8.51 | <0.001 | 6.32 | 5.48 | 7.29 | <0.001 | 5.87 | 5.21 | 6.62 | <0.001 |
| Antihypertensive | 1.14 | 0.83 | 1.58 | 0.281 | 1.08 | 0.82 | 1.43 | 0.464 | 1.10 | 0.86 | 1.40 | 0.311 |
| Aspirin | 0.87 | 0.48 | 1.60 | 0.562 | 1.03 | 0.65 | 1.63 | 0.877 | 0.88 | 0.58 | 1.35 | 0.450 |
| Anticoagulants | 1.02 | 0.45 | 2.32 | 0.951 | 0.79 | 0.36 | 1.73 | 0.444 | 0.93 | 0.48 | 1.79 | 0.767 |
| NSAIDs | 0.85 | 0.59 | 1.22 | 0.253 | 1.03 | 0.81 | 1.32 | 0.730 | 1.05 | 0.85 | 1.30 | 0.526 |
| Anticonvulsants | 1.43 | 0.92 | 2.23 | 0.035 | 1.41 | 0.99 | 2.01 | 0.013 | 1.34 | 0.98 | 1.83 | 0.016 |
| Hypnotics | 2.18 | 1.84 | 2.58 | <0.001 | 1.99 | 1.74 | 2.28 | <0.001 | 1.92 | 1.71 | 2.15 | <0.001 |
| Bisphosphonates | 1.69 | 0.44 | 6.51 | 0.316 | 1.42 | 0.42 | 4.81 | 0.460 | 1.09 | 0.33 | 3.62 | 0.849 |
| Contraceptives | 1.01 | 0.79 | 1.29 | 0.942 | 0.90 | 0.76 | 1.06 | 0.104 | 0.86 | 0.74 | 1.00 | 0.013 |
| *Constant* | 0.01 | 0.00 | 0.01 | <0.001 | 0.02 | 0.01 | 0.03 | <0.001 | 0.02 | 0.01 | 0.03 | <0.001 |

### **Table A10e.** Regression analyses for safety (suicidality) at 2 months, 6 months, and 12 months; complete case analysis (CCA) and full set analysis (FSA)

|  | **Safety, suicidality *(CCA,* *N= 63,801)*** | | | | | | | | | | | |
| --- | --- | --- | --- | --- | --- | --- | --- | --- | --- | --- | --- | --- |
|  | **2 months*)*** | | | | **6 months** | | | | **12 months** | | | |
|  | *OR* | *99% CI* | | *P* | *OR* | *99% CI* | | *P* | *OR* | *99% CI* | | *P* |
| ***Statins*** | 1.07 | 0.70 | 1.64 | 0.684 | 0.94 | 0.63 | 1.40 | 0.693 | 0.97 | 0.66 | 1.43 | 0.840 |
| Antidepressant category (SSRIs) |  |  |  |  |  |  |  |  |  |  |  |  |
| TCAs | 1.40 | 0.81 | 2.42 | 0.117 | 1.46 | 0.94 | 2.28 | 0.028 | 1.32 | 0.84 | 2.10 | 0.115 |
| MAOIs |  |  |  |  |  |  |  |  |  |  |  |  |
| Other antidepressants | 1.04 | 0.71 | 1.54 | 0.786 | 1.04 | 0.75 | 1.42 | 0.780 | 1.08 | 0.82 | 1.43 | 0.458 |
|  |  |  |  |  |  |  |  |  |  |  |  |  |
| PHQ-9 baseline | 1.02 | 1.00 | 1.05 | 0.025 | 1.02 | 1.00 | 1.05 | 0.018 | 1.03 | 1.00 | 1.05 | 0.007 |
| BMI | 0.99 | 0.97 | 1.00 | 0.066 | 0.99 | 0.98 | 1.01 | 0.138 | 0.99 | 0.98 | 1.01 | 0.164 |
|  |  |  |  |  |  |  |  |  |  |  |  |  |
| Read codes for depression (Major depression) |  |  |  |  |  |  |  |  |  |  |  |  |
| Minor depression | 1.61 | 0.88 | 2.95 | 0.044 | 1.54 | 0.88 | 2.72 | 0.047 | 1.55 | 0.91 | 2.64 | 0.034 |
| Other | 1.42 | 0.81 | 2.49 | 0.107 | 1.00 | 0.59 | 1.71 | 0.995 | 1.01 | 0.61 | 1.65 | 0.975 |
|  |  |  |  |  |  |  |  |  |  |  |  |  |
| Townsend deprivation score in fifths (1, least deprived) |  |  |  |  |  |  |  |  |  |  |  |  |
| 2 | 0.88 | 0.61 | 1.26 | 0.357 | 0.86 | 0.60 | 1.23 | 0.281 | 0.88 | 0.63 | 1.23 | 0.318 |
| 3 | 1.08 | 0.67 | 1.72 | 0.690 | 1.08 | 0.71 | 1.65 | 0.648 | 1.07 | 0.71 | 1.62 | 0.664 |
| 4 | 1.76 | 1.06 | 2.94 | 0.004 | 1.77 | 1.09 | 2.90 | 0.003 | 1.72 | 1.08 | 2.74 | 0.003 |
| 5, most deprived | 2.57 | 1.48 | 4.46 | <0.001 | 2.48 | 1.53 | 4.01 | <0.001 | 2.43 | 1.53 | 3.85 | <0.001 |
|  |  |  |  |  |  |  |  |  |  |  |  |  |
| Region of England (East Midlands) |  |  |  |  |  |  |  |  |  |  |  |  |
| East of England | 0.60 | 0.19 | 1.94 | 0.265 | 0.37 | 0.12 | 1.09 | 0.017 | 0.46 | 0.16 | 1.34 | 0.062 |
| London | 1.66 | 0.55 | 5.02 | 0.235 | 1.54 | 0.57 | 4.16 | 0.264 | 1.54 | 0.61 | 3.91 | 0.232 |
| North East | 0.20 | 0.05 | 0.74 | 0.002 | 0.31 | 0.09 | 1.04 | 0.013 | 0.35 | 0.11 | 1.08 | 0.016 |
| North West | 0.55 | 0.16 | 1.84 | 0.201 | 0.49 | 0.17 | 1.42 | 0.085 | 0.49 | 0.18 | 1.31 | 0.062 |
| South Central | 0.75 | 0.20 | 2.77 | 0.568 | 0.58 | 0.17 | 1.94 | 0.245 | 0.56 | 0.19 | 1.72 | 0.187 |
| South East | 2.38 | 0.68 | 8.28 | 0.073 | 2.13 | 0.67 | 6.71 | 0.091 | 2.02 | 0.68 | 6.05 | 0.097 |
| South West | 0.48 | 0.13 | 1.87 | 0.166 | 0.49 | 0.13 | 1.83 | 0.165 | 0.49 | 0.14 | 1.67 | 0.133 |
| West Midlands | 0.37 | 0.12 | 1.18 | 0.028 | 0.44 | 0.16 | 1.21 | 0.037 | 0.53 | 0.20 | 1.36 | 0.082 |
| Yorkshire & Humber | 2.18 | 0.25 | 18.86 | 0.352 | 1.80 | 0.23 | 14.07 | 0.459 | 1.67 | 0.22 | 12.45 | 0.510 |
|  |  |  |  |  |  |  |  |  |  |  |  |  |
| Smoking status (Non-smoker) |  |  |  |  |  |  |  |  |  |  |  |  |
| Ex-smoker | 0.97 | 0.77 | 1.21 | 0.690 | 1.01 | 0.84 | 1.22 | 0.872 | 0.94 | 0.79 | 1.12 | 0.390 |
| Light-smoker | 0.87 | 0.69 | 1.11 | 0.150 | 0.96 | 0.79 | 1.15 | 0.533 | 0.97 | 0.80 | 1.16 | 0.624 |
| Moderate-smoker | 0.79 | 0.37 | 1.70 | 0.434 | 0.70 | 0.37 | 1.31 | 0.144 | 0.83 | 0.50 | 1.38 | 0.341 |
| Heavy-smoker | 1.11 | 0.48 | 2.56 | 0.753 | 0.86 | 0.38 | 1.95 | 0.642 | 1.06 | 0.49 | 2.26 | 0.853 |
|  |  |  |  |  |  |  |  |  |  |  |  |  |
| Alcohol use (Non-drinker/trivial) |  |  |  |  |  |  |  |  |  |  |  |  |
| Light | 0.93 | 0.75 | 1.15 | 0.392 | 0.98 | 0.82 | 1.19 | 0.813 | 0.97 | 0.82 | 1.14 | 0.636 |
| Medium | 0.94 | 0.69 | 1.28 | 0.587 | 0.99 | 0.74 | 1.32 | 0.925 | 1.02 | 0.80 | 1.30 | 0.850 |
| Heavy | 1.29 | 0.73 | 2.26 | 0.246 | 1.20 | 0.71 | 2.03 | 0.372 | 1.30 | 0.82 | 2.08 | 0.141 |
| Very heavy | 0.91 | 0.48 | 1.72 | 0.698 | 0.95 | 0.55 | 1.61 | 0.786 | 1.10 | 0.70 | 1.70 | 0.596 |
|  |  |  |  |  |  |  |  |  |  |  |  |  |
| Ethnic group (White) |  |  |  |  |  |  |  |  |  |  |  |  |
| African/Caribbean | 0.94 | 0.59 | 1.50 | 0.725 | 1.05 | 0.71 | 1.55 | 0.745 | 1.03 | 0.73 | 1.45 | 0.850 |
| Asian | 1.08 | 0.65 | 1.80 | 0.695 | 1.10 | 0.71 | 1.69 | 0.579 | 1.11 | 0.74 | 1.65 | 0.520 |
| Other | 0.92 | 0.61 | 1.38 | 0.584 | 0.84 | 0.58 | 1.21 | 0.218 | 0.82 | 0.59 | 1.15 | 0.128 |
|  |  |  |  |  |  |  |  |  |  |  |  |  |
| Age | 1.00 | 0.98 | 1.01 | 0.336 | 0.99 | 0.98 | 1.00 | 0.064 | 0.99 | 0.98 | 1.00 | 0.021 |
|  |  |  |  |  |  |  |  |  |  |  |  |  |
| Sex (Female) |  |  |  |  |  |  |  |  |  |  |  |  |
| Male | 1.27 | 1.07 | 1.50 | <0.001 | 1.28 | 1.10 | 1.49 | <0.001 | 1.28 | 1.11 | 1.47 | <0.001 |
|  |  |  |  |  |  |  |  |  |  |  |  |  |
| Year of diagnosis (1998-2005) |  |  |  |  |  |  |  |  |  |  |  |  |
| 2006 to 2010 | 0.69 | 0.11 | 4.37 | 0.600 | 1.08 | 0.17 | 6.64 | 0.917 | 0.68 | 0.21 | 2.22 | 0.404 |
| 2011 to 2015 | 0.66 | 0.10 | 4.19 | 0.564 | 0.90 | 0.15 | 5.45 | 0.886 | 0.59 | 0.19 | 1.84 | 0.234 |
| 2016 to 2020 | 0.71 | 0.11 | 4.46 | 0.634 | 1.03 | 0.17 | 6.11 | 0.962 | 0.63 | 0.21 | 1.90 | 0.279 |
|  |  |  |  |  |  |  |  |  |  |  |  |  |
| Coronary heart disease | 0.57 | 0.27 | 1.19 | 0.050 | 0.70 | 0.38 | 1.29 | 0.132 | 0.88 | 0.50 | 1.54 | 0.554 |
| Stroke | 0.57 | 0.21 | 1.55 | 0.147 | 0.86 | 0.38 | 1.94 | 0.627 | 0.93 | 0.47 | 1.86 | 0.784 |
| Diabetes | 0.87 | 0.56 | 1.33 | 0.389 | 1.02 | 0.70 | 1.48 | 0.905 | 0.97 | 0.68 | 1.37 | 0.803 |
| Epilepsy | 0.69 | 0.23 | 2.04 | 0.379 | 0.73 | 0.32 | 1.63 | 0.307 | 0.79 | 0.40 | 1.58 | 0.385 |
| Hypothyroidism | 0.90 | 0.53 | 1.52 | 0.594 | 0.88 | 0.55 | 1.40 | 0.470 | 0.80 | 0.51 | 1.25 | 0.191 |
| Arthritis | 0.70 | 0.39 | 1.25 | 0.109 | 0.84 | 0.55 | 1.29 | 0.290 | 0.85 | 0.57 | 1.28 | 0.308 |
| Anxiety | 0.98 | 0.80 | 1.21 | 0.813 | 1.01 | 0.85 | 1.21 | 0.839 | 1.03 | 0.87 | 1.22 | 0.629 |
| Migraine | 0.94 | 0.63 | 1.39 | 0.669 | 0.97 | 0.71 | 1.32 | 0.783 | 0.94 | 0.70 | 1.26 | 0.591 |
| Cancer | 0.87 | 0.62 | 1.23 | 0.303 | 0.82 | 0.62 | 1.09 | 0.073 | 0.81 | 0.62 | 1.05 | 0.038 |
| Asthma | 1.09 | 0.85 | 1.39 | 0.389 | 1.11 | 0.91 | 1.36 | 0.188 | 1.14 | 0.95 | 1.37 | 0.066 |
| Renal failure | 2.19 | 0.67 | 7.20 | 0.090 | 1.80 | 0.60 | 5.39 | 0.167 | 2.04 | 0.82 | 5.09 | 0.044 |
| Liver failure | 1.07 | 0.51 | 2.24 | 0.823 | 1.06 | 0.55 | 2.06 | 0.813 | 1.13 | 0.64 | 2.01 | 0.569 |
| Osteoporosis | 1.09 | 0.40 | 2.96 | 0.815 | 1.06 | 0.42 | 2.68 | 0.868 | 1.03 | 0.43 | 2.44 | 0.932 |
| Suicidality | 3.92 | 2.81 | 5.46 | <0.001 | 3.65 | 2.68 | 4.96 | <0.001 | 3.52 | 2.64 | 4.69 | <0.001 |
| Antihypertensive | 1.18 | 0.81 | 1.71 | 0.256 | 1.15 | 0.83 | 1.59 | 0.281 | 1.12 | 0.82 | 1.52 | 0.354 |
| Aspirin | 1.27 | 0.69 | 2.31 | 0.313 | 1.02 | 0.61 | 1.69 | 0.937 | 1.01 | 0.65 | 1.58 | 0.949 |
| Anticoagulants | 0.87 | 0.29 | 2.60 | 0.746 | 0.81 | 0.29 | 2.28 | 0.607 | 0.85 | 0.34 | 2.12 | 0.648 |
| NSAIDs | 1.30 | 0.80 | 2.09 | 0.164 | 1.35 | 0.93 | 1.96 | 0.040 | 1.39 | 0.98 | 1.97 | 0.016 |
| Anticonvulsants | 0.61 | 0.21 | 1.78 | 0.234 | 0.91 | 0.42 | 1.96 | 0.746 | 0.82 | 0.39 | 1.69 | 0.474 |
| Hypnotics | 1.09 | 0.73 | 1.63 | 0.583 | 1.04 | 0.76 | 1.42 | 0.771 | 1.00 | 0.75 | 1.34 | 0.975 |
| Bisphosphonates | 2.76 | 0.80 | 9.55 | 0.035 | 2.47 | 0.66 | 9.24 | 0.077 | 2.10 | 0.56 | 7.92 | 0.151 |
| Contraceptives | 1.04 | 0.77 | 1.42 | 0.731 | 1.05 | 0.81 | 1.35 | 0.638 | 0.98 | 0.79 | 1.22 | 0.791 |
| *Constant* | 0.01 | 0.00 | 0.11 | <0.001 | 0.01 | 0.00 | 0.11 | <0.001 | 0.03 | 0.01 | 0.11 | <0.001 |

|  | **Safety, suicidality *(FSA, N = 673,177)*** | | | | | | | | | | | |
| --- | --- | --- | --- | --- | --- | --- | --- | --- | --- | --- | --- | --- |
|  | **2 months** | | | | **6 months** | | | | **12 months** | | | |
|  | *OR* | *99% CI* | | *P* | *OR* | *99% CI* | | *P* | *OR* | *99% CI* | | *P* |
| ***Statins*** | 1.08 | 0.89 | 1.31 | 0.298 | 1.07 | 0.91 | 1.27 | 0.272 | 1.04 | 0.89 | 1.20 | 0.550 |
| Antidepressant category (SSRIs) |  |  |  |  |  |  |  |  |  |  |  |  |
| TCAs | 1.28 | 1.03 | 1.59 | 0.004 | 1.20 | 1.00 | 1.44 | 0.008 | 1.13 | 0.96 | 1.33 | 0.052 |
| MAOIs | 1.00 |  |  |  | 1.10 | 0.08 | 14.65 | 0.921 | 0.84 | 0.06 | 11.08 | 0.860 |
| Other antidepressants | 1.16 | 0.99 | 1.36 | 0.017 | 1.23 | 1.07 | 1.41 | <0.001 | 1.24 | 1.10 | 1.40 | <0.001 |
|  |  |  |  |  |  |  |  |  |  |  |  |  |
| PHQ-9 baseline | 1.01 | 1.00 | 1.02 | 0.040 | 1.01 | 1.00 | 1.02 | 0.023 | 1.01 | 1.00 | 1.02 | 0.004 |
| BMI | 0.99 | 0.99 | 1.00 | 0.060 | 1.00 | 0.99 | 1.00 | 0.117 | 1.00 | 0.99 | 1.00 | 0.536 |
|  |  |  |  |  |  |  |  |  |  |  |  |  |
| Read codes for depression (Major depression) |  |  |  |  |  |  |  |  |  |  |  |  |
| Minor depression | 1.23 | 0.93 | 1.64 | 0.060 | 1.15 | 0.89 | 1.48 | 0.153 | 1.12 | 0.89 | 1.40 | 0.203 |
| Other | 1.14 | 0.85 | 1.52 | 0.241 | 1.02 | 0.80 | 1.30 | 0.825 | 1.01 | 0.83 | 1.23 | 0.851 |
|  |  |  |  |  |  |  |  |  |  |  |  |  |
| Townsend deprivation score in fifths (1, least deprived) |  |  |  |  |  |  |  |  |  |  |  |  |
| 2 | 1.03 | 0.87 | 1.22 | 0.677 | 1.01 | 0.86 | 1.20 | 0.821 | 1.03 | 0.89 | 1.19 | 0.625 |
| 3 | 1.09 | 0.89 | 1.33 | 0.292 | 1.12 | 0.93 | 1.35 | 0.111 | 1.15 | 0.98 | 1.36 | 0.028 |
| 4 | 1.40 | 1.10 | 1.78 | <0.001 | 1.43 | 1.14 | 1.80 | <0.001 | 1.47 | 1.20 | 1.80 | <0.001 |
| 5, most deprived | 2.08 | 1.59 | 2.72 | <0.001 | 2.04 | 1.58 | 2.64 | <0.001 | 2.08 | 1.64 | 2.62 | <0.001 |
|  |  |  |  |  |  |  |  |  |  |  |  |  |
| Region of England (East Midlands) |  |  |  |  |  |  |  |  |  |  |  |  |
| East of England | 0.56 | 0.22 | 1.47 | 0.123 | 0.53 | 0.21 | 1.34 | 0.078 | 0.54 | 0.23 | 1.26 | 0.062 |
| London | 1.25 | 0.59 | 2.63 | 0.443 | 1.27 | 0.59 | 2.69 | 0.423 | 1.25 | 0.63 | 2.48 | 0.399 |
| North East | 0.52 | 0.21 | 1.25 | 0.054 | 0.59 | 0.24 | 1.42 | 0.123 | 0.63 | 0.27 | 1.47 | 0.158 |
| North West | 0.66 | 0.29 | 1.50 | 0.191 | 0.66 | 0.30 | 1.45 | 0.174 | 0.67 | 0.33 | 1.35 | 0.140 |
| South Central | 0.43 | 0.19 | 0.94 | 0.006 | 0.44 | 0.20 | 0.96 | 0.006 | 0.47 | 0.23 | 0.95 | 0.006 |
| South East | 1.33 | 0.51 | 3.50 | 0.447 | 1.29 | 0.50 | 3.34 | 0.486 | 1.26 | 0.53 | 2.99 | 0.500 |
| South West | 0.59 | 0.27 | 1.30 | 0.086 | 0.57 | 0.26 | 1.24 | 0.062 | 0.59 | 0.29 | 1.20 | 0.055 |
| West Midlands | 0.42 | 0.20 | 0.90 | 0.003 | 0.46 | 0.22 | 0.99 | 0.009 | 0.49 | 0.24 | 0.97 | 0.007 |
| Yorkshire & Humber | 0.76 | 0.19 | 2.99 | 0.610 | 0.73 | 0.20 | 2.68 | 0.532 | 0.73 | 0.23 | 2.37 | 0.496 |
|  |  |  |  |  |  |  |  |  |  |  |  |  |
| Smoking status (Non-smoker) |  |  |  |  |  |  |  |  |  |  |  |  |
| Ex-smoker | 0.96 | 0.85 | 1.08 | 0.355 | 0.95 | 0.86 | 1.05 | 0.200 | 0.94 | 0.86 | 1.03 | 0.071 |
| Light-smoker | 0.94 | 0.84 | 1.05 | 0.126 | 0.97 | 0.88 | 1.07 | 0.375 | 1.00 | 0.91 | 1.09 | 0.963 |
| Moderate-smoker | 0.89 | 0.64 | 1.24 | 0.365 | 0.85 | 0.64 | 1.12 | 0.133 | 0.89 | 0.71 | 1.13 | 0.229 |
| Heavy-smoker | 0.86 | 0.55 | 1.37 | 0.410 | 0.96 | 0.68 | 1.36 | 0.761 | 0.97 | 0.72 | 1.31 | 0.789 |
|  |  |  |  |  |  |  |  |  |  |  |  |  |
| Alcohol use (Non-drinker/trivial) |  |  |  |  |  |  |  |  |  |  |  |  |
| Light | 0.99 | 0.89 | 1.10 | 0.873 | 1.00 | 0.92 | 1.08 | 0.920 | 1.00 | 0.93 | 1.08 | 0.953 |
| Medium | 1.06 | 0.90 | 1.25 | 0.355 | 1.06 | 0.93 | 1.22 | 0.235 | 1.05 | 0.93 | 1.19 | 0.310 |
| Heavy | 1.19 | 0.83 | 1.70 | 0.191 | 1.11 | 0.85 | 1.46 | 0.294 | 1.14 | 0.87 | 1.48 | 0.202 |
| Very heavy | 1.20 | 0.92 | 1.56 | 0.082 | 1.17 | 0.93 | 1.47 | 0.068 | 1.23 | 1.02 | 1.48 | 0.004 |
|  |  |  |  |  |  |  |  |  |  |  |  |  |
| Ethnic group (White) |  |  |  |  |  |  |  |  |  |  |  |  |
| African/Caribbean | 0.93 | 0.77 | 1.12 | 0.336 | 0.96 | 0.82 | 1.13 | 0.532 | 0.94 | 0.81 | 1.08 | 0.236 |
| Asian | 1.36 | 1.09 | 1.70 | <0.001 | 1.34 | 1.09 | 1.65 | <0.001 | 1.32 | 1.08 | 1.62 | <0.001 |
| Other | 1.12 | 0.92 | 1.37 | 0.136 | 1.14 | 0.98 | 1.34 | 0.027 | 1.14 | 0.97 | 1.33 | 0.034 |
|  |  |  |  |  |  |  |  |  |  |  |  |  |
| Age | 0.99 | 0.98 | 0.99 | <0.001 | 0.99 | 0.98 | 0.99 | <0.001 | 0.98 | 0.98 | 0.99 | <0.001 |
|  |  |  |  |  |  |  |  |  |  |  |  |  |
| Sex (Female) |  |  |  |  |  |  |  |  |  |  |  |  |
| Male | 1.32 | 1.21 | 1.43 | <0.001 | 1.29 | 1.19 | 1.39 | <0.001 | 1.27 | 1.19 | 1.36 | <0.001 |
|  |  |  |  |  |  |  |  |  |  |  |  |  |
| Year of diagnosis (1998-2005) |  |  |  |  |  |  |  |  |  |  |  |  |
| 2006 to 2010 | 3.64 | 2.43 | 5.46 | <0.001 | 3.43 | 2.43 | 4.84 | <0.001 | 3.04 | 2.26 | 4.09 | <0.001 |
| 2011 to 2015 | 3.45 | 2.12 | 5.63 | <0.001 | 3.05 | 2.04 | 4.56 | <0.001 | 2.75 | 1.96 | 3.87 | <0.001 |
| 2016 to 2020 | 3.17 | 1.88 | 5.35 | <0.001 | 2.88 | 1.85 | 4.47 | <0.001 | 2.60 | 1.78 | 3.79 | <0.001 |
|  |  |  |  |  |  |  |  |  |  |  |  |  |
| Coronary heart disease | 0.87 | 0.64 | 1.16 | 0.210 | 0.86 | 0.67 | 1.11 | 0.125 | 0.92 | 0.74 | 1.15 | 0.344 |
| Stroke | 0.86 | 0.63 | 1.17 | 0.198 | 0.98 | 0.78 | 1.24 | 0.833 | 0.99 | 0.80 | 1.24 | 0.925 |
| Diabetes | 0.96 | 0.80 | 1.17 | 0.629 | 1.02 | 0.87 | 1.20 | 0.700 | 1.03 | 0.89 | 1.18 | 0.648 |
| Epilepsy | 0.91 | 0.66 | 1.24 | 0.426 | 0.89 | 0.69 | 1.15 | 0.252 | 0.91 | 0.73 | 1.14 | 0.281 |
| Hypothyroidism | 1.03 | 0.85 | 1.24 | 0.733 | 0.97 | 0.82 | 1.15 | 0.618 | 0.92 | 0.78 | 1.08 | 0.160 |
| Arthritis | 0.79 | 0.64 | 0.97 | 0.003 | 0.86 | 0.73 | 1.02 | 0.020 | 0.93 | 0.80 | 1.07 | 0.187 |
| Anxiety | 1.12 | 1.02 | 1.24 | 0.002 | 1.17 | 1.08 | 1.28 | <0.001 | 1.18 | 1.10 | 1.28 | <0.001 |
| Migraine | 1.00 | 0.86 | 1.16 | 0.995 | 0.97 | 0.86 | 1.09 | 0.489 | 0.97 | 0.87 | 1.08 | 0.465 |
| Cancer | 0.95 | 0.82 | 1.11 | 0.431 | 0.91 | 0.79 | 1.03 | 0.055 | 0.91 | 0.81 | 1.02 | 0.029 |
| Asthma | 1.01 | 0.91 | 1.12 | 0.802 | 1.03 | 0.94 | 1.12 | 0.425 | 1.05 | 0.98 | 1.13 | 0.089 |
| Renal failure | 0.74 | 0.36 | 1.54 | 0.292 | 0.86 | 0.49 | 1.52 | 0.500 | 0.97 | 0.59 | 1.61 | 0.885 |
| Liver failure | 0.80 | 0.56 | 1.16 | 0.123 | 0.84 | 0.62 | 1.14 | 0.148 | 0.86 | 0.65 | 1.14 | 0.164 |
| Osteoporosis | 1.04 | 0.71 | 1.53 | 0.787 | 0.95 | 0.67 | 1.34 | 0.683 | 0.92 | 0.67 | 1.26 | 0.472 |
| Suicidality | 3.53 | 3.01 | 4.14 | <0.001 | 3.55 | 3.07 | 4.10 | <0.001 | 3.42 | 2.98 | 3.92 | <0.001 |
| Antihypertensive | 1.12 | 0.96 | 1.32 | 0.062 | 1.11 | 0.97 | 1.27 | 0.052 | 1.06 | 0.94 | 1.20 | 0.198 |
| Aspirin | 0.94 | 0.72 | 1.22 | 0.520 | 0.89 | 0.71 | 1.11 | 0.164 | 0.91 | 0.76 | 1.09 | 0.172 |
| Anticoagulants | 0.78 | 0.49 | 1.23 | 0.155 | 0.70 | 0.47 | 1.05 | 0.025 | 0.69 | 0.48 | 0.99 | 0.008 |
| NSAIDs | 1.02 | 0.87 | 1.20 | 0.712 | 1.12 | 0.99 | 1.27 | 0.022 | 1.16 | 1.03 | 1.31 | 0.001 |
| Anticonvulsants | 1.41 | 1.11 | 1.78 | <0.001 | 1.39 | 1.13 | 1.70 | <0.001 | 1.37 | 1.14 | 1.66 | <0.001 |
| Hypnotics | 1.37 | 1.19 | 1.57 | <0.001 | 1.30 | 1.16 | 1.46 | <0.001 | 1.29 | 1.16 | 1.44 | <0.001 |
| Bisphosphonates | 1.19 | 0.59 | 2.38 | 0.531 | 1.40 | 0.79 | 2.47 | 0.131 | 1.22 | 0.71 | 2.09 | 0.348 |
| Contraceptives | 0.90 | 0.77 | 1.04 | 0.061 | 0.88 | 0.78 | 1.00 | 0.008 | 0.85 | 0.76 | 0.95 | <0.001 |
| *Constant* | 0.00 | 0.00 | 0.01 | <0.001 | 0.01 | 0.00 | 0.02 | <0.001 | 0.01 | 0.00 | 0.02 | <0.001 |

### **Table A10f.** Regression analyses for safety (completed suicide) at 2 months, 6 months, and 12 months; complete case analysis (CCA) and full set analysis (FSA)

|  | **Safety, completed suicide *(CCA)*** | | | | | | | | | | | |
| --- | --- | --- | --- | --- | --- | --- | --- | --- | --- | --- | --- | --- |
|  | **2 months *(N= 41,940)*** | | | | **6 months *(N= 49,496)*** | | | | **12 months *(N= 53,588)*** | | | |
|  | *OR* | *99% CI* | | *P* | *OR* | *99% CI* | | *P* | *OR* | *99% CI* | | *P* |
| ***Statins*** | 0.81 | 0.01 | 81.94 | 0.908 | 0.36 | 0.01 | 13.65 | 0.473 | 0.32 | 0.01 | 10.19 | 0.394 |
| Antidepressant category (SSRIs) |  |  |  |  |  |  |  |  |  |  |  |  |
| TCAs | 1.00 |  |  |  |  |  |  |  | 1.14 | 0.09 | 14.15 | 0.894 |
| MAOIs |  |  |  |  |  |  |  |  |  |  |  |  |
| Other antidepressants | 1.00 |  |  |  | 0.49 | 0.04 | 5.95 | 0.462 | 0.81 | 0.13 | 4.99 | 0.769 |
|  |  |  |  |  |  |  |  |  |  |  |  |  |
| PHQ-9 baseline | 0.96 | 0.78 | 1.18 | 0.610 | 1.03 | 0.88 | 1.20 | 0.675 | 1.03 | 0.88 | 1.21 | 0.642 |
| BMI | 0.99 | 0.89 | 1.09 | 0.745 | 1.00 | 0.93 | 1.07 | 0.973 | 1.02 | 0.95 | 1.09 | 0.513 |
|  |  |  |  |  |  |  |  |  |  |  |  |  |
| Read codes for depression (Major depression) |  |  |  |  |  |  |  |  |  |  |  |  |
| Minor depression | 2.21 | 0.52 | 9.33 | 0.156 | 1.43 | 0.51 | 4.06 | 0.372 | 1.80 | 0.76 | 4.24 | 0.077 |
| Other | 4.17 | 0.24 | 71.57 | 0.196 | 1.82 | 0.12 | 28.01 | 0.574 | 3.14 | 0.47 | 21.03 | 0.121 |
|  |  |  |  |  |  |  |  |  |  |  |  |  |
| Townsend deprivation score in fifths (1, least deprived) |  |  |  |  |  |  |  |  |  |  |  |  |
| 2 | 0.69 | 0.12 | 3.90 | 0.585 | 0.40 | 0.09 | 1.76 | 0.112 | 0.53 | 0.14 | 2.04 | 0.222 |
| 3 | 0.76 | 0.09 | 6.37 | 0.738 | 0.51 | 0.11 | 2.45 | 0.271 | 1.08 | 0.31 | 3.71 | 0.877 |
| 4 | 1.22 | 0.12 | 12.06 | 0.820 | 0.84 | 0.19 | 3.75 | 0.757 | 1.14 | 0.29 | 4.50 | 0.805 |
| 5, most deprived | 0.90 | 0.05 | 17.57 | 0.925 | 0.66 | 0.11 | 3.97 | 0.548 | 0.97 | 0.18 | 5.34 | 0.966 |
|  |  |  |  |  |  |  |  |  |  |  |  |  |
| Region of England (East Midlands) |  |  |  |  |  |  |  |  |  |  |  |  |
| East of England | 1.00 |  |  |  |  |  |  |  | 1.13 | 0.04 | 36.45 | 0.928 |
| London | 0.13 | 0.00 | 4.67 | 0.141 | 1.01 | 0.08 | 13.46 | 0.992 | 1.26 | 0.11 | 14.47 | 0.810 |
| North East | 1.00 |  |  |  | 0.74 | 0.02 | 24.43 | 0.826 | 0.68 | 0.02 | 21.58 | 0.777 |
| North West | 0.18 | 0.00 | 7.75 | 0.242 | 0.52 | 0.03 | 10.37 | 0.578 | 0.70 | 0.04 | 10.93 | 0.736 |
| South Central | 1.51 | 0.14 | 15.94 | 0.653 | 1.38 | 0.10 | 19.25 | 0.750 | 1.88 | 0.14 | 25.23 | 0.533 |
| South East | 0.27 | 0.01 | 9.27 | 0.336 | 0.58 | 0.03 | 11.80 | 0.641 | 0.88 | 0.06 | 13.13 | 0.906 |
| South West | 0.80 | 0.04 | 14.95 | 0.843 | 2.33 | 0.17 | 31.03 | 0.400 | 2.26 | 0.18 | 28.80 | 0.410 |
| West Midlands | 0.96 | 0.07 | 12.80 | 0.968 | 1.16 | 0.08 | 16.81 | 0.885 | 1.40 | 0.11 | 18.21 | 0.735 |
| Yorkshire & Humber | 1.25 | 0.06 | 24.69 | 0.847 | 1.76 | 0.10 | 31.10 | 0.611 | 2.27 | 0.15 | 34.12 | 0.436 |
|  |  |  |  |  |  |  |  |  |  |  |  |  |
| Smoking status (Non-smoker) |  |  |  |  |  |  |  |  |  |  |  |  |
| Ex-smoker | 0.52 | 0.06 | 4.21 | 0.424 | 1.64 | 0.50 | 5.33 | 0.284 | 0.93 | 0.32 | 2.69 | 0.853 |
| Light-smoker | 1.03 | 0.19 | 5.49 | 0.968 | 1.65 | 0.47 | 5.74 | 0.302 | 1.12 | 0.41 | 3.12 | 0.767 |
| Moderate-smoker | 1.00 |  |  |  |  |  |  |  | 1.00 |  |  |  |
| Heavy-smoker | 1.00 |  |  |  |  |  |  |  | 1.00 |  |  |  |
|  |  |  |  |  |  |  |  |  |  |  |  |  |
| Alcohol use (Non-drinker/trivial) |  |  |  |  |  |  |  |  |  |  |  |  |
| Light | 1.85 | 0.42 | 8.25 | 0.287 | 1.92 | 0.62 | 5.99 | 0.138 | 2.07 | 0.77 | 5.54 | 0.057 |
| Medium | 2.10 | 0.21 | 20.49 | 0.402 | 3.20 | 0.81 | 12.62 | 0.029 | 2.28 | 0.60 | 8.59 | 0.110 |
| Heavy | 6.92 | 0.34 | 141.55 | 0.099 | 6.23 | 0.82 | 47.46 | 0.020 | 4.39 | 0.63 | 30.57 | 0.050 |
| Very heavy | 9.25 | 1.14 | 75.23 | 0.006 | 5.79 | 0.94 | 35.79 | 0.013 | 4.44 | 0.78 | 25.39 | 0.028 |
|  |  |  |  |  |  |  |  |  |  |  |  |  |
| Ethnic group (White) |  |  |  |  |  |  |  |  |  |  |  |  |
| African/Caribbean | 1.00 |  |  |  |  |  |  |  | 1.00 |  |  |  |
| Asian | 1.00 |  |  |  |  |  |  |  | 0.84 | 0.06 | 12.12 | 0.869 |
| Other | 2.82 | 0.14 | 58.39 | 0.379 | 0.99 | 0.06 | 16.20 | 0.994 | 1.50 | 0.21 | 10.64 | 0.592 |
|  |  |  |  |  |  |  |  |  |  |  |  |  |
| Age | 0.98 | 0.93 | 1.04 | 0.413 | 0.99 | 0.95 | 1.04 | 0.664 | 1.00 | 0.96 | 1.04 | 0.905 |
|  |  |  |  |  |  |  |  |  |  |  |  |  |
| Sex (Female) |  |  |  |  |  |  |  |  |  |  |  |  |
| Male | 2.80 | 0.60 | 13.11 | 0.085 | \| 1.70198 | 0.66 | 4.38 | 0.148 | 1.84 | 0.81 | 4.22 | 0.057 |
|  |  |  |  |  |  |  |  |  |  |  |  |  |
| Year of diagnosis (1998-2005) |  |  |  |  |  |  |  |  |  |  |  |  |
| 2006 to 2010 | 1.33 | 0.19 | 9.05 | 0.705 | 1.87 | 0.37 | 9.43 | 0.321 | 2.65 | 0.55 | 12.84 | 0.112 |
| 2011 to 2015 | 1.33 | 0.18 | 9.88 | 0.714 | 1.50 | 0.29 | 7.66 | 0.520 | 2.23 | 0.46 | 10.74 | 0.190 |
| 2016 to 2020 | 1.00 |  |  |  |  |  |  |  | 1.00 |  |  |  |
|  |  |  |  |  |  |  |  |  |  |  |  |  |
| Coronary heart disease | 1.00 |  |  |  |  |  |  |  | 1.00 |  |  |  |
| Stroke | 1.00 |  |  |  |  |  |  |  | 1.00 |  |  |  |
| Diabetes | 4.42 | 0.22 | 87.31 | 0.199 | 4.07 | 0.49 | 33.97 | 0.088 | 2.93 | 0.42 | 20.65 | 0.157 |
| Epilepsy | 1.00 |  |  |  |  |  |  |  | 1.00 |  |  |  |
| Hypothyroidism | 1.00 |  |  |  |  |  |  |  | 1.00 |  |  |  |
| Arthritis | 1.78 | 0.08 | 39.36 | 0.630 | 0.71 | 0.04 | 13.25 | 0.764 | 0.52 | 0.03 | 8.74 | 0.550 |
| Anxiety | 0.94 | 0.12 | 7.16 | 0.938 | 0.91 | 0.21 | 3.90 | 0.873 | 1.16 | 0.34 | 3.87 | 0.759 |
| Migraine | 1.00 |  |  |  | 0.53 | 0.04 | 6.82 | 0.522 | 0.40 | 0.03 | 4.93 | 0.343 |
| Cancer | 1.41 | 0.09 | 23.31 | 0.751 | 0.60 | 0.04 | 8.49 | 0.617 | 0.44 | 0.03 | 6.25 | 0.428 |
| Asthma | 0.29 | 0.02 | 4.04 | 0.226 | 0.62 | 0.16 | 2.44 | 0.375 | 0.77 | 0.25 | 2.34 | 0.541 |
| Renal failure | 1.00 |  |  |  |  |  |  |  | 1.00 |  |  |  |
| Liver failure | 6.41 | 0.25 | 166.47 | 0.142 | 2.42 | 0.11 | 53.42 | 0.463 | 2.16 | 0.12 | 38.26 | 0.492 |
| Osteoporosis | 1.00 |  |  |  |  |  |  |  | 1.00 |  |  |  |
| Suicidality | 6.67 | 1.22 | 36.50 | 0.004 | 4.62 | 1.17 | 18.25 | 0.004 | 3.44 | 0.96 | 12.35 | 0.013 |
| Antihypertensive | 0.73 | 0.03 | 19.61 | 0.807 | 0.92 | 0.09 | 9.23 | 0.930 | 0.71 | 0.08 | 6.37 | 0.690 |
| Aspirin | 1.00 |  |  |  |  |  |  |  | 1.00 |  |  |  |
| Anticoagulants | 1.00 |  |  |  |  |  |  |  | 1.00 |  |  |  |
| NSAIDs | 2.54 | 0.14 | 47.84 | 0.413 | 1.11 | 0.07 | 16.34 | 0.924 | 1.72 | 0.25 | 11.68 | 0.463 |
| Anticonvulsants | 1.00 |  |  |  | 6.86 | 0.30 | 155.14 | 0.112 | 4.89 | 0.30 | 78.92 | 0.142 |
| Hypnotics | 3.13 | 0.59 | 16.73 | 0.079 | 1.52 | 0.37 | 6.35 | 0.447 | 2.15 | 0.70 | 6.60 | 0.079 |
| Bisphosphonates | 1.00 |  |  |  |  |  |  |  | 1.00 |  |  |  |
| Contraceptives | 3.92 | 0.35 | 44.04 | 0.146 | 1.39 | 0.17 | 11.22 | 0.682 | 1.06 | 0.14 | 8.11 | 0.940 |
| *Constant* | 0.00 | 0.00 | 0.67 | 0.007 | 0.00 | 4.69 | 0.03 | <0.001 | 0.00 | 0.00 | 0.01 | <0.001 |

|  | **Safety, completed suicide *(FSA, N = 673,177)*** | | | | | | | | | | | |
| --- | --- | --- | --- | --- | --- | --- | --- | --- | --- | --- | --- | --- |
|  | **2 months** | | | | **6 months** | | | | **12 months** | | | |
|  | *OR* | *99% CI* | | *P* | *OR* | *99% CI* | | *P* | *OR* | *99% CI* | | *P* |
| ***Statins*** | 0.98 | 0.24 | 3.98 | 0.977 | 0.81 | 0.28 | 2.36 | 0.609 | 1.09 | 0.48 | 2.47 | 0.794 |
| Antidepressant category (SSRIs) |  |  |  |  |  |  |  |  |  |  |  |  |
| TCAs | 0.70 | 0.27 | 1.76 | 0.314 | 0.90 | 0.47 | 1.73 | 0.680 | 0.96 | 0.56 | 1.62 | 0.832 |
| MAOIs | 1.00 |  |  |  |  |  |  |  | 10.96 | 0.78 | 154.60 | 0.020 |
| Other antidepressants | 1.29 | 0.65 | 2.56 | 0.344 | 1.41 | 0.85 | 2.33 | 0.080 | 1.69 | 1.14 | 2.51 | 0.001 |
|  |  |  |  |  |  |  |  |  |  |  |  |  |
| PHQ-9 baseline | 1.00 | 0.96 | 1.05 | 0.896 | 1.01 | 0.97 | 1.05 | 0.585 | 1.02 | 0.98 | 1.05 | 0.225 |
| BMI | 1.00 | 0.96 | 1.04 | 0.859 | 1.00 | 0.97 | 1.03 | 0.851 | 1.00 | 0.97 | 1.02 | 0.747 |
|  |  |  |  |  |  |  |  |  |  |  |  |  |
| Read codes for depression (Major depression) |  |  |  |  |  |  |  |  |  |  |  |  |
| Minor depression | 0.86 | 0.54 | 1.36 | 0.399 | 0.77 | 0.52 | 1.14 | 0.087 | 0.88 | 0.64 | 1.22 | 0.318 |
| Other | 0.75 | 0.24 | 2.39 | 0.529 | 0.95 | 0.44 | 2.07 | 0.871 | 1.08 | 0.58 | 2.01 | 0.762 |
|  |  |  |  |  |  |  |  |  |  |  |  |  |
| Townsend deprivation score in fifths (1, least deprived) |  |  |  |  |  |  |  |  |  |  |  |  |
| 2 | 1.05 | 0.54 | 2.07 | 0.838 | 0.86 | 0.50 | 1.46 | 0.456 | 0.96 | 0.61 | 1.51 | 0.826 |
| 3 | 1.36 | 0.73 | 2.56 | 0.204 | 1.26 | 0.77 | 2.06 | 0.222 | 1.48 | 1.00 | 2.21 | 0.011 |
| 4 | 1.49 | 0.76 | 2.92 | 0.125 | 1.25 | 0.74 | 2.10 | 0.271 | 1.38 | 0.88 | 2.19 | 0.067 |
| 5, most deprived | 1.01 | 0.48 | 2.14 | 0.973 | 1.03 | 0.58 | 1.85 | 0.888 | 1.29 | 0.77 | 2.15 | 0.202 |
|  |  |  |  |  |  |  |  |  |  |  |  |  |
| Region of England (East Midlands) |  |  |  |  |  |  |  |  |  |  |  |  |
| East of England | 1.01 | 0.28 | 3.62 | 0.985 | 1.23 | 0.42 | 3.63 | 0.626 | 1.67 | 0.64 | 4.34 | 0.170 |
| London | 0.79 | 0.27 | 2.27 | 0.558 | 1.01 | 0.45 | 2.30 | 0.971 | 1.21 | 0.56 | 2.60 | 0.528 |
| North East | 1.43 | 0.39 | 5.18 | 0.478 | 1.54 | 0.41 | 5.79 | 0.402 | 1.58 | 0.40 | 6.19 | 0.387 |
| North West | 1.06 | 0.36 | 3.06 | 0.894 | 1.11 | 0.48 | 2.60 | 0.746 | 1.44 | 0.66 | 3.14 | 0.230 |
| South Central | 1.21 | 0.41 | 3.54 | 0.649 | 1.17 | 0.49 | 2.77 | 0.639 | 1.59 | 0.70 | 3.60 | 0.144 |
| South East | 0.96 | 0.31 | 2.92 | 0.918 | 0.91 | 0.38 | 2.21 | 0.793 | 1.22 | 0.54 | 2.77 | 0.536 |
| South West | 1.27 | 0.42 | 3.86 | 0.580 | 1.35 | 0.55 | 3.31 | 0.394 | 1.77 | 0.76 | 4.07 | 0.080 |
| West Midlands | 0.70 | 0.20 | 2.40 | 0.456 | 0.79 | 0.31 | 2.01 | 0.515 | 0.90 | 0.37 | 2.20 | 0.751 |
| Yorkshire & Humber | 1.11 | 0.33 | 3.72 | 0.817 | 1.10 | 0.39 | 3.12 | 0.809 | 1.62 | 0.58 | 4.48 | 0.224 |
|  |  |  |  |  |  |  |  |  |  |  |  |  |
| Smoking status (Non-smoker) |  |  |  |  |  |  |  |  |  |  |  |  |
| Ex-smoker | 0.91 | 0.45 | 1.84 | 0.731 | 1.09 | 0.68 | 1.76 | 0.645 | 0.87 | 0.57 | 1.32 | 0.396 |
| Light-smoker | 1.31 | 0.81 | 2.12 | 0.151 | 1.15 | 0.78 | 1.69 | 0.350 | 1.07 | 0.77 | 1.48 | 0.604 |
| Moderate-smoker | 2.57 | 0.97 | 6.83 | 0.013 | 1.50 | 0.58 | 3.87 | 0.275 | 1.44 | 0.68 | 3.07 | 0.213 |
| Heavy-smoker | 0.92 | 0.15 | 5.79 | 0.909 | 0.88 | 0.20 | 3.78 | 0.817 | 0.74 | 0.20 | 2.67 | 0.543 |
|  |  |  |  |  |  |  |  |  |  |  |  |  |
| Alcohol use (Non-drinker/trivial) |  |  |  |  |  |  |  |  |  |  |  |  |
| Light | 1.20 | 0.67 | 2.17 | 0.400 | 1.21 | 0.78 | 1.87 | 0.255 | 1.18 | 0.81 | 1.72 | 0.245 |
| Medium | 1.33 | 0.64 | 2.75 | 0.309 | 1.31 | 0.79 | 2.18 | 0.171 | 1.21 | 0.77 | 1.90 | 0.273 |
| Heavy | 1.54 | 0.35 | 6.82 | 0.447 | 1.76 | 0.60 | 5.19 | 0.174 | 1.63 | 0.61 | 4.35 | 0.194 |
| Very heavy | 2.18 | 0.73 | 6.56 | 0.066 | 2.20 | 0.97 | 5.00 | 0.014 | 2.06 | 0.97 | 4.36 | 0.013 |
|  |  |  |  |  |  |  |  |  |  |  |  |  |
| Ethnic group (White) |  |  |  |  |  |  |  |  |  |  |  |  |
| African/Caribbean | 1.14 | 0.41 | 3.21 | 0.737 | 1.00 | 0.38 | 2.68 | 0.989 | 0.89 | 0.40 | 1.95 | 0.688 |
| Asian | 0.61 | 0.17 | 2.21 | 0.322 | 0.72 | 0.30 | 1.71 | 0.329 | 0.97 | 0.47 | 1.98 | 0.900 |
| Other | 0.93 | 0.24 | 3.55 | 0.889 | 1.11 | 0.46 | 2.69 | 0.765 | 1.14 | 0.55 | 2.36 | 0.644 |
|  |  |  |  |  |  |  |  |  |  |  |  |  |
| Age | 0.98 | 0.96 | 0.99 | <0.001 | 0.98 | 0.96 | 0.99 | <0.001 | 0.98 | 0.97 | 0.99 | <0.001 |
|  |  |  |  |  |  |  |  |  |  |  |  |  |
| Sex (Female) |  |  |  |  |  |  |  |  |  |  |  |  |
| Male | 1.92 | 1.28 | 2.88 | <0.001 | 1.73 | 1.27 | 2.35 | <0.001 | 1.61 | 1.23 | 2.10 | <0.001 |
|  |  |  |  |  |  |  |  |  |  |  |  |  |
| Year of diagnosis (1998-2005) |  |  |  |  |  |  |  |  |  |  |  |  |
| 2006 to 2010 | 0.83 | 0.51 | 1.37 | 0.340 | 0.83 | 0.56 | 1.24 | 0.244 | 0.82 | 0.58 | 1.14 | 0.119 |
| 2011 to 2015 | 0.48 | 0.27 | 0.85 | 0.001 | 0.52 | 0.33 | 0.82 | <0.001 | 0.48 | 0.33 | 0.71 | <0.001 |
| 2016 to 2020 | 0.27 | 0.15 | 0.50 | <0.001 | 0.34 | 0.20 | 0.56 | <0.001 | 0.31 | 0.20 | 0.48 | <0.001 |
|  |  |  |  |  |  |  |  |  |  |  |  |  |
| Coronary heart disease | 0.76 | 0.15 | 3.78 | 0.659 | 0.57 | 0.14 | 2.29 | 0.297 | 0.58 | 0.18 | 1.91 | 0.242 |
| Stroke | 1.18 | 0.21 | 6.67 | 0.801 | 1.19 | 0.33 | 4.35 | 0.725 | 0.92 | 0.28 | 2.96 | 0.846 |
| Diabetes | 0.88 | 0.23 | 3.41 | 0.811 | 0.92 | 0.31 | 2.75 | 0.850 | 0.68 | 0.26 | 1.83 | 0.319 |
| Epilepsy | 1.00 | 0.21 | 4.69 | 0.995 | 0.92 | 0.23 | 3.58 | 0.870 | 1.63 | 0.59 | 4.49 | 0.217 |
| Hypothyroidism | 1.00 |  |  |  | 0.18 | 0.01 | 2.38 | 0.088 | 0.12 | 0.01 | 1.58 | 0.034 |
| Arthritis | 1.81 | 0.74 | 4.45 | 0.088 | 1.23 | 0.56 | 2.73 | 0.493 | 1.14 | 0.58 | 2.25 | 0.606 |
| Anxiety | 0.67 | 0.32 | 1.40 | 0.158 | 0.83 | 0.51 | 1.35 | 0.318 | 0.93 | 0.62 | 1.41 | 0.668 |
| Migraine | 0.19 | 0.03 | 1.20 | 0.021 | 0.35 | 0.12 | 0.99 | 0.009 | 0.50 | 0.24 | 1.03 | 0.013 |
| Cancer | 0.96 | 0.36 | 2.53 | 0.908 | 1.14 | 0.53 | 2.46 | 0.671 | 1.24 | 0.68 | 2.26 | 0.355 |
| Asthma | 0.97 | 0.57 | 1.64 | 0.872 | 1.15 | 0.77 | 1.74 | 0.371 | 1.04 | 0.72 | 1.48 | 0.805 |
| Renal failure | 1.00 |  |  |  |  |  |  |  | 0.95 | 0.07 | 12.91 | 0.957 |
| Liver failure | 3.12 | 0.83 | 11.68 | 0.027 | 1.84 | 0.50 | 6.75 | 0.228 | 1.66 | 0.51 | 5.39 | 0.269 |
| Osteoporosis | 0.92 | 0.03 | 24.09 | 0.946 | 1.28 | 0.15 | 11.29 | 0.768 | 1.33 | 0.25 | 7.22 | 0.661 |
| Suicidality | 7.10 | 4.27 | 11.79 | <0.001 | 6.10 | 4.02 | 9.26 | <0.001 | 5.76 | 4.01 | 8.29 | <0.001 |
| Antihypertensive | 1.21 | 0.45 | 3.26 | 0.624 | 1.02 | 0.45 | 2.32 | 0.948 | 0.89 | 0.45 | 1.76 | 0.657 |
| Aspirin | 0.82 | 0.18 | 3.79 | 0.745 | 1.24 | 0.42 | 3.65 | 0.601 | 1.09 | 0.43 | 2.78 | 0.803 |
| Anticoagulants | 0.86 | 0.06 | 12.44 | 0.882 | 0.56 | 0.04 | 7.69 | 0.571 | 0.38 | 0.03 | 5.14 | 0.340 |
| NSAIDs | 0.92 | 0.34 | 2.48 | 0.832 | 1.14 | 0.56 | 2.33 | 0.640 | 1.00 | 0.54 | 1.88 | 0.986 |
| Anticonvulsants | 1.85 | 0.46 | 7.46 | 0.257 | 1.80 | 0.54 | 5.93 | 0.207 | 1.16 | 0.38 | 3.52 | 0.726 |
| Hypnotics | 2.02 | 1.23 | 3.32 | <0.001 | 2.33 | 1.60 | 3.39 | <0.001 | 2.42 | 1.77 | 3.32 | <0.001 |
| Bisphosphonates | 3.99 | 0.15 | 104.21 | 0.275 | 1.92 | 0.09 | 41.21 | 0.585 | 1.14 | 0.06 | 21.11 | 0.906 |
| Contraceptives | 1.15 | 0.49 | 2.69 | 0.668 | 0.75 | 0.36 | 1.59 | 0.331 | 0.73 | 0.39 | 1.37 | 0.199 |
| *Constant* | 0.00 | 0.00 | 0.00 | <0.001 | 0.00 | 0.00 | 0.00 | <0.001 | 0.00 | 0.00 | 0.00 | <0.001 |

### **Table A10g.** Regression analyses for safety (all-cause mortality) at 2 months, 6 months, and 12 months; complete case analysis (CCA) and full set analysis (FSA)

|  | **Safety, all-cause mortality *(CCA)*** | | | | | | | | | | | |
| --- | --- | --- | --- | --- | --- | --- | --- | --- | --- | --- | --- | --- |
|  | **2 months *(N= 63,282)*** | | | | **6 months *(N= 63,282)*** | | | | **12 months *(N= 63,706)*** | | | |
|  | *OR* | *99% CI* | | *P* | *OR* | *99% CI* | | *P* | *OR* | *99% CI* | | *P* |
| ***Statins*** | 0.66 | 0.36 | 1.19 | 0.066 | 0.66 | 0.36 | 1.19 | 0.066 | 0.69 | 0.52 | 0.92 | 0.001 |
| Antidepressant category (SSRIs) |  |  |  |  |  |  |  |  |  |  |  |  |
| TCAs | 0.49 | 0.07 | 3.27 | 0.331 | 0.49 | 0.07 | 3.27 | 0.331 | 1.03 | 0.54 | 1.96 | 0.919 |
| MAOIs |  |  |  |  |  |  |  |  |  |  |  |  |
| Other antidepressants | 1.53 | 0.76 | 3.09 | 0.118 | 1.53 | 0.76 | 3.09 | 0.118 | 1.01 | 0.68 | 1.52 | 0.943 |
|  |  |  |  |  |  |  |  |  |  |  |  |  |
| PHQ-9 baseline | 1.07 | 1.02 | 1.11 | <0.001 | 1.07 | 1.02 | 1.11 | <0.001 | 1.05 | 1.02 | 1.07 | <0.001 |
| BMI | 0.95 | 0.90 | 1.00 | 0.010 | 0.95 | 0.90 | 1.00 | 0.010 | 0.96 | 0.93 | 0.99 | <0.001 |
|  |  |  |  |  |  |  |  |  |  |  |  |  |
| Read codes for depression (Major depression) |  |  |  |  |  |  |  |  |  |  |  |  |
| Minor depression | 0.69 | 0.40 | 1.20 | 0.087 | 0.69 | 0.40 | 1.20 | 0.087 | 0.95 | 0.73 | 1.24 | 0.603 |
| Other | 2.00 | 0.59 | 6.74 | 0.141 | 2.00 | 0.59 | 6.74 | 0.141 | 1.65 | 0.82 | 3.33 | 0.066 |
|  |  |  |  |  |  |  |  |  |  |  |  |  |
| Townsend deprivation score in fifths (1, least deprived) |  |  |  |  |  |  |  |  |  |  |  |  |
| 2 | 1.14 | 0.64 | 2.03 | 0.572 | 1.14 | 0.64 | 2.03 | 0.572 | 1.38 | 1.01 | 1.89 | 0.007 |
| 3 | 1.26 | 0.70 | 2.26 | 0.308 | 1.26 | 0.70 | 2.26 | 0.308 | 1.30 | 0.94 | 1.80 | 0.039 |
| 4 | 0.62 | 0.26 | 1.47 | 0.155 | 0.62 | 0.26 | 1.47 | 0.155 | 1.39 | 0.96 | 2.02 | 0.021 |
| 5, most deprived | 1.06 | 0.45 | 2.52 | 0.856 | 1.06 | 0.45 | 2.52 | 0.856 | 1.05 | 0.68 | 1.62 | 0.774 |
|  |  |  |  |  |  |  |  |  |  |  |  |  |
| Region of England (East Midlands) |  |  |  |  |  |  |  |  |  |  |  |  |
| East of England | 0.77 | 0.13 | 4.72 | 0.710 | 0.77 | 0.13 | 4.72 | 0.710 | 0.90 | 0.38 | 2.15 | 0.759 |
| London | 1.34 | 0.34 | 5.32 | 0.580 | 1.34 | 0.34 | 5.32 | 0.580 | 1.27 | 0.67 | 2.42 | 0.334 |
| North East | 0.67 | 0.09 | 4.74 | 0.594 | 0.67 | 0.09 | 4.74 | 0.594 | 0.83 | 0.28 | 2.46 | 0.655 |
| North West | 0.97 | 0.24 | 3.86 | 0.948 | 0.97 | 0.24 | 3.86 | 0.948 | 1.16 | 0.63 | 2.15 | 0.535 |
| South Central | 1.03 | 0.26 | 4.05 | 0.955 | 1.03 | 0.26 | 4.05 | 0.955 | 1.01 | 0.53 | 1.92 | 0.973 |
| South East | 0.63 | 0.13 | 3.05 | 0.447 | 0.63 | 0.13 | 3.05 | 0.447 | 0.77 | 0.39 | 1.49 | 0.302 |
| South West | 0.63 | 0.13 | 3.01 | 0.448 | 0.63 | 0.13 | 3.01 | 0.448 | 0.88 | 0.44 | 1.75 | 0.624 |
| West Midlands | 1.66 | 0.43 | 6.44 | 0.335 | 1.66 | 0.43 | 6.44 | 0.335 | 1.25 | 0.65 | 2.39 | 0.378 |
| Yorkshire & Humber | 1.16 | 0.25 | 5.44 | 0.809 | 1.16 | 0.25 | 5.44 | 0.809 | 1.09 | 0.50 | 2.35 | 0.782 |
|  |  |  |  |  |  |  |  |  |  |  |  |  |
| Smoking status (Non-smoker) |  |  |  |  |  |  |  |  |  |  |  |  |
| Ex-smoker | 1.15 | 0.68 | 1.94 | 0.482 | 1.15 | 0.68 | 1.94 | 0.482 | 1.20 | 0.91 | 1.59 | 0.083 |
| Light-smoker | 1.05 | 0.54 | 2.03 | 0.852 | 1.05 | 0.54 | 2.03 | 0.852 | 1.73 | 1.24 | 2.41 | <0.001 |
| Moderate-smoker | 0.82 | 0.05 | 12.90 | 0.851 | 0.82 | 0.05 | 12.90 | 0.851 | 1.18 | 0.33 | 4.18 | 0.738 |
| Heavy-smoker | 1.00 |  |  |  | 1.00 |  |  |  | 1.32 | 0.27 | 6.34 | 0.651 |
|  |  |  |  |  |  |  |  |  |  |  |  |  |
| Alcohol use (Non-drinker/trivial) |  |  |  |  |  |  |  |  |  |  |  |  |
| Light | 1.13 | 0.68 | 1.89 | 0.537 | 1.13 | 0.68 | 1.89 | 0.537 | 1.28 | 0.98 | 1.66 | 0.018 |
| Medium | 0.86 | 0.37 | 1.99 | 0.652 | 0.86 | 0.37 | 1.99 | 0.652 | 1.31 | 0.89 | 1.92 | 0.068 |
| Heavy | 1.28 | 0.20 | 8.27 | 0.729 | 1.28 | 0.20 | 8.27 | 0.729 | 1.28 | 0.51 | 3.21 | 0.492 |
| Very heavy | 1.74 | 0.42 | 7.17 | 0.316 | 1.74 | 0.42 | 7.17 | 0.316 | 2.09 | 1.05 | 4.17 | 0.006 |
|  |  |  |  |  |  |  |  |  |  |  |  |  |
| Ethnic group (White) |  |  |  |  |  |  |  |  |  |  |  |  |
| African/Caribbean | 0.47 | 0.04 | 5.93 | 0.442 | 0.47 | 0.04 | 5.93 | 0.442 | 0.73 | 0.25 | 2.08 | 0.434 |
| Asian | 0.44 | 0.04 | 5.39 | 0.399 | 0.44 | 0.04 | 5.39 | 0.399 | 0.70 | 0.24 | 1.98 | 0.371 |
| Other | 1.23 | 0.17 | 8.88 | 0.784 | 1.23 | 0.17 | 8.88 | 0.784 | 0.77 | 0.27 | 2.22 | 0.522 |
|  |  |  |  |  |  |  |  |  |  |  |  |  |
| Age | 1.09 | 1.07 | 1.11 | <0.001 | 1.09 | 1.07 | 1.11 | <0.001 | 1.09 | 1.08 | 1.10 | <0.001 |
|  |  |  |  |  |  |  |  |  |  |  |  |  |
| Sex (Female) |  |  |  |  |  |  |  |  |  |  |  |  |
| Male | 1.69 | 1.01 | 2.82 | 0.008 | 1.69 | 1.01 | 2.82 | 0.008 | 1.64 | 1.27 | 2.11 | <0.001 |
|  |  |  |  |  |  |  |  |  |  |  |  |  |
| Year of diagnosis (1998-2005) |  |  |  |  |  |  |  |  |  |  |  |  |
| 2006 to 2010 | 1.27 | 0.57 | 2.85 | 0.449 | 1.27 | 0.57 | 2.85 | 0.449 | 0.73 | 0.53 | 1.02 | 0.017 |
| 2011 to 2015 | 1.17 | 0.52 | 2.65 | 0.612 | 1.17 | 0.52 | 2.65 | 0.612 | 0.78 | 0.56 | 1.08 | 0.050 |
| 2016 to 2020 | 1.00 |  |  |  | 1.00 |  |  |  | 1.00 |  |  |  |
|  |  |  |  |  |  |  |  |  |  |  |  |  |
| Coronary heart disease | 1.00 | 0.50 | 2.01 | 0.993 | 1.00 | 0.50 | 2.01 | 0.993 | 1.32 | 0.95 | 1.82 | 0.029 |
| Stroke | 1.40 | 0.74 | 2.65 | 0.180 | 1.40 | 0.74 | 2.65 | 0.180 | 1.40 | 1.00 | 1.96 | 0.010 |
| Diabetes | 1.16 | 0.62 | 2.16 | 0.552 | 1.16 | 0.62 | 2.16 | 0.552 | 1.47 | 1.07 | 2.03 | 0.002 |
| Epilepsy | 2.62 | 0.53 | 13.11 | 0.122 | 2.62 | 0.53 | 13.11 | 0.122 | 1.00 | 0.38 | 2.61 | 0.991 |
| Hypothyroidism | 1.02 | 0.42 | 2.48 | 0.954 | 1.02 | 0.42 | 2.48 | 0.954 | 0.98 | 0.62 | 1.55 | 0.910 |
| Arthritis | 0.81 | 0.45 | 1.47 | 0.365 | 0.81 | 0.45 | 1.47 | 0.365 | 0.94 | 0.71 | 1.25 | 0.576 |
| Anxiety | 0.53 | 0.22 | 1.26 | 0.059 | 0.53 | 0.22 | 1.26 | 0.059 | 0.74 | 0.50 | 1.10 | 0.052 |
| Migraine | 0.84 | 0.24 | 2.91 | 0.720 | 0.84 | 0.24 | 2.91 | 0.720 | 1.25 | 0.77 | 2.05 | 0.240 |
| Cancer | 2.11 | 1.29 | 3.47 | <0.001 | 2.11 | 1.29 | 3.47 | <0.001 | 2.51 | 1.94 | 3.24 | <0.001 |
| Asthma | 1.16 | 0.67 | 2.02 | 0.489 | 1.16 | 0.67 | 2.02 | 0.489 | 1.24 | 0.93 | 1.65 | 0.054 |
| Renal failure | 3.40 | 1.00 | 11.55 | 0.010 | 3.40 | 1.00 | 11.55 | 0.010 | 2.99 | 1.48 | 6.04 | <0.001 |
| Liver failure | 2.23 | 0.58 | 8.54 | 0.124 | 2.23 | 0.58 | 8.54 | 0.124 | 1.94 | 0.95 | 3.95 | 0.017 |
| Osteoporosis | 0.95 | 0.28 | 3.22 | 0.922 | 0.95 | 0.28 | 3.22 | 0.922 | 1.27 | 0.74 | 2.20 | 0.259 |
| Suicidality | 1.57 | 0.35 | 7.11 | 0.444 | 1.57 | 0.35 | 7.11 | 0.444 | 0.69 | 0.24 | 2.05 | 0.384 |
| Antihypertensive | 1.32 | 0.75 | 2.32 | 0.201 | 1.32 | 0.75 | 2.32 | 0.201 | 1.19 | 0.90 | 1.57 | 0.116 |
| Aspirin | 1.31 | 0.67 | 2.55 | 0.302 | 1.31 | 0.67 | 2.55 | 0.302 | 1.26 | 0.90 | 1.76 | 0.081 |
| Anticoagulants | 2.71 | 1.28 | 5.74 | 0.001 | 2.71 | 1.28 | 5.74 | 0.001 | 2.17 | 1.39 | 3.36 | <0.001 |
| NSAIDs | 1.93 | 0.73 | 5.07 | 0.081 | 1.93 | 0.73 | 5.07 | 0.081 | 1.12 | 0.65 | 1.91 | 0.601 |
| Anticonvulsants | 0.51 | 0.08 | 3.32 | 0.350 | 0.51 | 0.08 | 3.32 | 0.350 | 1.35 | 0.59 | 3.11 | 0.348 |
| Hypnotics | 1.31 | 0.67 | 2.53 | 0.298 | 1.31 | 0.67 | 2.53 | 0.298 | 0.98 | 0.67 | 1.42 | 0.874 |
| Bisphosphonates | 0.50 | 0.03 | 8.98 | 0.538 | 0.50 | 0.03 | 8.98 | 0.538 | 0.68 | 0.21 | 2.16 | 0.386 |
| Contraceptives | 1.21 | 0.08 | 18.00 | 0.858 | 1.21 | 0.08 | 18.00 | 0.858 | 0.57 | 0.09 | 3.59 | 0.426 |
| *Constant* | 0.00 | 0.00 | 0.00 | <0.001 | 0.00 | 0.00 | 0.00 | <0.001 | 0.00 | 0.00 | 0.00 | <0.001 |

|  | **Safety, all-cause mortality *(FSA, N = 673,177)*** | | | | | | | | | | | |
| --- | --- | --- | --- | --- | --- | --- | --- | --- | --- | --- | --- | --- |
|  | **2 months** | | | | **6 months** | | | | **12 months** | | | |
|  | *OR* | *99% CI* | | *P* | *OR* | *99% CI* | | *P* | *OR* | *99% CI* | | *P* |
| ***Statins*** | 0.69 | 0.62 | 0.78 | <0.001 | 0.69 | 0.62 | 0.78 | <0.001 | 0.72 | 0.67 | 0.77 | <0.001 |
| Antidepressant category (SSRIs) |  |  |  |  |  |  |  |  |  |  |  |  |
| TCAs | 0.92 | 0.79 | 1.07 | 0.144 | 0.92 | 0.79 | 1.07 | 0.144 | 0.96 | 0.88 | 1.06 | 0.297 |
| MAOIs | 1.50 | 0.08 | 28.09 | 0.719 | 1.50 | 0.08 | 28.09 | 0.719 | 0.77 | 0.11 | 5.19 | 0.719 |
| Other antidepressants | 1.22 | 1.09 | 1.38 | <0.001 | 1.22 | 1.09 | 1.38 | <0.001 | 1.18 | 1.09 | 1.27 | <0.001 |
|  |  |  |  |  |  |  |  |  |  |  |  |  |
| PHQ-9 baseline | 1.01 | 0.99 | 1.02 | 0.117 | 1.01 | 0.99 | 1.02 | 0.117 | 1.01 | 1.00 | 1.01 | 0.008 |
| BMI | 0.98 | 0.97 | 0.99 | <0.001 | 0.98 | 0.97 | 0.99 | <0.001 | 0.99 | 0.98 | 0.99 | <0.001 |
|  |  |  |  |  |  |  |  |  |  |  |  |  |
| Read codes for depression (Major depression) |  |  |  |  |  |  |  |  |  |  |  |  |
| Minor depression | 1.23 | 1.11 | 1.35 | <0.001 | 1.23 | 1.11 | 1.35 | <0.001 | 1.25 | 1.18 | 1.33 | <0.001 |
| Other | 0.96 | 0.73 | 1.28 | 0.738 | 0.96 | 0.73 | 1.28 | 0.738 | 1.15 | 0.98 | 1.33 | 0.021 |
|  |  |  |  |  |  |  |  |  |  |  |  |  |
| Townsend deprivation score in fifths (1, least deprived) |  |  |  |  |  |  |  |  |  |  |  |  |
| 2 | 1.08 | 0.96 | 1.21 | 0.092 | 1.08 | 0.96 | 1.21 | 0.092 | 1.04 | 0.96 | 1.11 | 0.225 |
| 3 | 1.14 | 1.00 | 1.29 | 0.008 | 1.14 | 1.00 | 1.29 | 0.008 | 1.09 | 1.00 | 1.18 | 0.011 |
| 4 | 1.07 | 0.92 | 1.24 | 0.274 | 1.07 | 0.92 | 1.24 | 0.274 | 1.08 | 0.98 | 1.19 | 0.036 |
| 5, most deprived | 1.14 | 0.95 | 1.36 | 0.071 | 1.14 | 0.95 | 1.36 | 0.071 | 1.11 | 1.00 | 1.24 | 0.012 |
|  |  |  |  |  |  |  |  |  |  |  |  |  |
| Region of England (East Midlands) |  |  |  |  |  |  |  |  |  |  |  |  |
| East of England | 0.92 | 0.63 | 1.33 | 0.547 | 0.92 | 0.63 | 1.33 | 0.547 | 0.94 | 0.74 | 1.19 | 0.495 |
| London | 0.80 | 0.59 | 1.09 | 0.064 | 0.80 | 0.59 | 1.09 | 0.064 | 0.82 | 0.67 | 1.00 | 0.008 |
| North East | 0.83 | 0.60 | 1.16 | 0.154 | 0.83 | 0.60 | 1.16 | 0.154 | 0.97 | 0.76 | 1.24 | 0.766 |
| North West | 1.01 | 0.76 | 1.34 | 0.949 | 1.01 | 0.76 | 1.34 | 0.949 | 1.03 | 0.85 | 1.25 | 0.660 |
| South Central | 0.91 | 0.67 | 1.23 | 0.405 | 0.91 | 0.67 | 1.23 | 0.405 | 0.88 | 0.72 | 1.08 | 0.113 |
| South East | 0.88 | 0.64 | 1.21 | 0.297 | 0.88 | 0.64 | 1.21 | 0.297 | 0.84 | 0.68 | 1.04 | 0.040 |
| South West | 1.03 | 0.76 | 1.39 | 0.790 | 1.03 | 0.76 | 1.39 | 0.790 | 0.99 | 0.81 | 1.21 | 0.913 |
| West Midlands | 0.97 | 0.72 | 1.30 | 0.791 | 0.97 | 0.72 | 1.30 | 0.791 | 0.96 | 0.79 | 1.17 | 0.624 |
| Yorkshire & Humber | 0.96 | 0.69 | 1.34 | 0.743 | 0.96 | 0.69 | 1.34 | 0.743 | 0.93 | 0.75 | 1.17 | 0.435 |
|  |  |  |  |  |  |  |  |  |  |  |  |  |
| Smoking status (Non-smoker) |  |  |  |  |  |  |  |  |  |  |  |  |
| Ex-smoker | 0.97 | 0.87 | 1.08 | 0.469 | 0.97 | 0.87 | 1.08 | 0.469 | 1.03 | 0.97 | 1.10 | 0.250 |
| Light-smoker | 1.18 | 1.03 | 1.37 | 0.002 | 1.18 | 1.03 | 1.37 | 0.002 | 1.36 | 1.26 | 1.46 | <0.001 |
| Moderate-smoker | 1.41 | 0.89 | 2.23 | 0.052 | 1.41 | 0.89 | 2.23 | 0.052 | 1.37 | 1.06 | 1.78 | 0.002 |
| Heavy-smoker | 1.01 | 0.50 | 2.03 | 0.965 | 1.01 | 0.50 | 2.03 | 0.965 | 1.45 | 1.04 | 2.02 | 0.004 |
|  |  |  |  |  |  |  |  |  |  |  |  |  |
| Alcohol use (Non-drinker/trivial) |  |  |  |  |  |  |  |  |  |  |  |  |
| Light | 0.99 | 0.87 | 1.13 | 0.903 | 0.99 | 0.87 | 1.13 | 0.903 | 1.02 | 0.95 | 1.10 | 0.441 |
| Medium | 1.10 | 0.92 | 1.33 | 0.164 | 1.10 | 0.92 | 1.33 | 0.164 | 1.15 | 1.03 | 1.27 | 0.001 |
| Heavy | 1.12 | 0.61 | 2.06 | 0.612 | 1.12 | 0.61 | 2.06 | 0.612 | 1.08 | 0.84 | 1.40 | 0.406 |
| Very heavy | 1.20 | 0.76 | 1.89 | 0.291 | 1.20 | 0.76 | 1.89 | 0.291 | 1.38 | 1.09 | 1.75 | 0.001 |
|  |  |  |  |  |  |  |  |  |  |  |  |  |
| Ethnic group (White) |  |  |  |  |  |  |  |  |  |  |  |  |
| African/Caribbean | 1.26 | 0.90 | 1.77 | 0.065 | 1.26 | 0.90 | 1.77 | 0.065 | 1.37 | 1.14 | 1.64 | <0.001 |
| Asian | 0.75 | 0.50 | 1.12 | 0.068 | 0.75 | 0.50 | 1.12 | 0.068 | 0.61 | 0.49 | 0.77 | <0.001 |
| Other | 0.75 | 0.42 | 1.34 | 0.203 | 0.75 | 0.42 | 1.34 | 0.203 | 0.67 | 0.49 | 0.92 | 0.001 |
|  |  |  |  |  |  |  |  |  |  |  |  |  |
| Age | 1.10 | 1.10 | 1.10 | <0.001 | 1.10 | 1.10 | 1.10 | <0.001 | 1.10 | 1.10 | 1.10 | <0.001 |
|  |  |  |  |  |  |  |  |  |  |  |  |  |
| Sex (Female) |  |  |  |  |  |  |  |  |  |  |  |  |
| Male | 1.74 | 1.58 | 1.91 | <0.001 | 1.74 | 1.58 | 1.91 | <0.001 | 1.72 | 1.63 | 1.81 | <0.001 |
|  |  |  |  |  |  |  |  |  |  |  |  |  |
| Year of diagnosis (1998-2005) |  |  |  |  |  |  |  |  |  |  |  |  |
| 2006 to 2010 | 1.12 | 0.99 | 1.28 | 0.017 | 1.12 | 0.99 | 1.28 | 0.017 | 1.01 | 0.93 | 1.09 | 0.791 |
| 2011 to 2015 | 0.96 | 0.84 | 1.10 | 0.424 | 0.96 | 0.84 | 1.10 | 0.424 | 0.89 | 0.81 | 0.97 | <0.001 |
| 2016 to 2020 | 0.79 | 0.69 | 0.91 | <0.001 | 0.79 | 0.69 | 0.91 | <0.001 | 0.74 | 0.68 | 0.82 | <0.001 |
|  |  |  |  |  |  |  |  |  |  |  |  |  |
| Coronary heart disease | 1.17 | 1.04 | 1.32 | 0.001 | 1.17 | 1.04 | 1.32 | 0.001 | 1.21 | 1.12 | 1.31 | <0.001 |
| Stroke | 1.07 | 0.94 | 1.22 | 0.204 | 1.07 | 0.94 | 1.22 | 0.204 | 1.22 | 1.13 | 1.31 | <0.001 |
| Diabetes | 1.27 | 1.11 | 1.44 | <0.001 | 1.27 | 1.11 | 1.44 | <0.001 | 1.37 | 1.28 | 1.48 | <0.001 |
| Epilepsy | 0.85 | 0.61 | 1.18 | 0.209 | 0.85 | 0.61 | 1.18 | 0.209 | 0.94 | 0.77 | 1.15 | 0.452 |
| Hypothyroidism | 0.88 | 0.75 | 1.03 | 0.040 | 0.88 | 0.75 | 1.03 | 0.040 | 1.00 | 0.91 | 1.09 | 0.978 |
| Arthritis | 0.75 | 0.67 | 0.83 | <0.001 | 0.75 | 0.67 | 0.83 | <0.001 | 0.78 | 0.73 | 0.83 | <0.001 |
| Anxiety | 0.80 | 0.69 | 0.93 | <0.001 | 0.80 | 0.69 | 0.93 | <0.001 | 0.79 | 0.73 | 0.86 | <0.001 |
| Migraine | 0.50 | 0.37 | 0.69 | <0.001 | 0.50 | 0.37 | 0.69 | <0.001 | 0.64 | 0.55 | 0.74 | <0.001 |
| Cancer | 1.93 | 1.75 | 2.13 | <0.001 | 1.93 | 1.75 | 2.13 | <0.001 | 2.17 | 2.04 | 2.31 | <0.001 |
| Asthma | 1.09 | 0.98 | 1.21 | 0.041 | 1.09 | 0.98 | 1.21 | 0.041 | 1.21 | 1.13 | 1.29 | <0.001 |
| Renal failure | 1.91 | 1.46 | 2.51 | <0.001 | 1.91 | 1.46 | 2.51 | <0.001 | 2.33 | 1.96 | 2.78 | <0.001 |
| Liver failure | 1.74 | 1.25 | 2.43 | <0.001 | 1.74 | 1.25 | 2.43 | <0.001 | 1.86 | 1.52 | 2.27 | <0.001 |
| Osteoporosis | 1.04 | 0.87 | 1.25 | 0.578 | 1.04 | 0.87 | 1.25 | 0.578 | 1.12 | 1.01 | 1.25 | 0.004 |
| Suicidality | 1.36 | 0.96 | 1.93 | 0.023 | 1.36 | 0.96 | 1.93 | 0.023 | 1.28 | 1.03 | 1.59 | 0.003 |
| Antihypertensive | 0.78 | 0.70 | 0.86 | <0.001 | 0.78 | 0.70 | 0.86 | <0.001 | 0.87 | 0.81 | 0.93 | <0.001 |
| Aspirin | 1.10 | 0.97 | 1.24 | 0.053 | 1.10 | 0.97 | 1.24 | 0.053 | 1.15 | 1.07 | 1.24 | <0.001 |
| Anticoagulants | 1.53 | 1.29 | 1.80 | <0.001 | 1.53 | 1.29 | 1.80 | <0.001 | 1.58 | 1.43 | 1.75 | <0.001 |
| NSAIDs | 1.35 | 1.13 | 1.62 | <0.001 | 1.35 | 1.13 | 1.62 | <0.001 | 1.19 | 1.07 | 1.32 | <0.001 |
| Anticonvulsants | 1.71 | 1.36 | 2.14 | <0.001 | 1.71 | 1.36 | 2.14 | <0.001 | 1.76 | 1.53 | 2.02 | <0.001 |
| Hypnotics | 1.31 | 1.16 | 1.48 | <0.001 | 1.31 | 1.16 | 1.48 | <0.001 | 1.40 | 1.31 | 1.51 | <0.001 |
| Bisphosphonates | 1.19 | 0.90 | 1.58 | 0.110 | 1.19 | 0.90 | 1.58 | 0.110 | 1.26 | 1.04 | 1.52 | 0.002 |
| Contraceptives | 0.61 | 0.23 | 1.63 | 0.194 | 0.61 | 0.23 | 1.63 | 0.194 | 0.48 | 0.27 | 0.84 | 0.001 |
| *Constant* | 0.00 | 0.00 | 0.00 | <0.001 | 0.00 | 0.00 | 0.00 | <0.001 | 0.00 | 0.00 | 0.00 | <0.001 |

### **Table A10h.** Regression analyses for efficacy (response) at 2 months, 6 months, and 12 months; complete case analysis (CCA) and full set analysis (FSA)

|  | **Efficacy, response *(CCA)*** | | | | | | | | | | | |
| --- | --- | --- | --- | --- | --- | --- | --- | --- | --- | --- | --- | --- |
|  | **2 months (*N= 22,804)*** | | | | **6 months (*N= 5,035)*** | | | | **12 months (*N= 2,938)*** | | | |
|  | *OR* | *99% CI* | | *P* | *OR* | *99% CI* | | *P* | *OR* | *99% CI* | | *P* |
| ***Statins*** | 1.10 | 0.93 | 1.29 | 0.155 | 1.06 | 0.74 | 1.52 | 0.685 | 1.47 | 0.87 | 2.49 | 0.057 |
| Antidepressant category (SSRIs) |  |  |  |  |  |  |  |  |  |  |  |  |
| TCAs | 0.60 | 0.45 | 0.81 | <0.001 | 0.74 | 0.42 | 1.30 | 0.166 | 0.85 | 0.43 | 1.67 | 0.526 |
| MAOIs |  |  |  |  |  |  |  |  |  |  |  |  |
| Other antidepressants | 0.77 | 0.65 | 0.92 | <0.001 | 0.77 | 0.53 | 1.13 | 0.083 | 0.80 | 0.48 | 1.35 | 0.282 |
|  |  |  |  |  |  |  |  |  |  |  |  |  |
| PHQ-9 baseline | 1.02 | 1.01 | 1.03 | <0.001 | 1.04 | 1.02 | 1.05 | <0.001 | 1.05 | 1.03 | 1.07 | <0.001 |
| BMI | 0.99 | 0.99 | 1.00 | <0.001 | 0.98 | 0.97 | 1.00 | 0.001 | 0.99 | 0.97 | 1.01 | 0.243 |
|  |  |  |  |  |  |  |  |  |  |  |  |  |
| Read codes for depression (Major depression) |  |  |  |  |  |  |  |  |  |  |  |  |
| Minor depression | 0.93 | 0.84 | 1.02 | 0.044 | 0.86 | 0.72 | 1.03 | 0.035 | 0.88 | 0.67 | 1.15 | 0.204 |
| Other | 0.93 | 0.66 | 1.31 | 0.584 | 0.69 | 0.34 | 1.38 | 0.169 | 0.86 | 0.40 | 1.87 | 0.622 |
|  |  |  |  |  |  |  |  |  |  |  |  |  |
| Townsend deprivation score in fifths (1, least deprived) |  |  |  |  |  |  |  |  |  |  |  |  |
| 2 | 0.91 | 0.82 | 1.01 | 0.014 | 0.90 | 0.71 | 1.15 | 0.277 | 0.77 | 0.55 | 1.06 | 0.037 |
| 3 | 0.77 | 0.69 | 0.86 | <0.001 | 0.86 | 0.67 | 1.09 | 0.097 | 0.66 | 0.48 | 0.92 | 0.001 |
| 4 | 0.73 | 0.65 | 0.83 | <0.001 | 0.64 | 0.50 | 0.84 | <0.001 | 0.61 | 0.43 | 0.86 | <0.001 |
| 5, most deprived | 0.67 | 0.57 | 0.77 | <0.001 | 0.68 | 0.51 | 0.91 | 0.001 | 0.79 | 0.53 | 1.18 | 0.134 |
|  |  |  |  |  |  |  |  |  |  |  |  |  |
| Region of England (East Midlands) |  |  |  |  |  |  |  |  |  |  |  |  |
| East of England | 0.89 | 0.57 | 1.41 | 0.525 | 1.23 | 0.65 | 2.36 | 0.404 | 0.66 | 0.30 | 1.45 | 0.176 |
| London | 0.97 | 0.72 | 1.29 | 0.756 | 1.16 | 0.68 | 1.97 | 0.472 | 0.90 | 0.47 | 1.72 | 0.673 |
| North East | 0.95 | 0.67 | 1.34 | 0.708 | 1.00 | 0.53 | 1.89 | 0.999 | 0.75 | 0.26 | 2.12 | 0.470 |
| North West | 0.88 | 0.66 | 1.18 | 0.257 | 1.05 | 0.62 | 1.79 | 0.797 | 0.91 | 0.47 | 1.75 | 0.707 |
| South Central | 1.10 | 0.81 | 1.48 | 0.430 | 1.20 | 0.69 | 2.09 | 0.405 | 0.92 | 0.46 | 1.82 | 0.745 |
| South East | 0.99 | 0.74 | 1.32 | 0.937 | 1.01 | 0.57 | 1.76 | 0.981 | 0.72 | 0.36 | 1.41 | 0.204 |
| South West | 0.98 | 0.73 | 1.31 | 0.859 | 0.86 | 0.49 | 1.52 | 0.501 | 0.77 | 0.38 | 1.57 | 0.339 |
| West Midlands | 0.94 | 0.68 | 1.29 | 0.602 | 1.04 | 0.60 | 1.79 | 0.864 | 1.06 | 0.54 | 2.09 | 0.831 |
| Yorkshire & Humber | 0.86 | 0.62 | 1.20 | 0.255 | 1.05 | 0.57 | 1.93 | 0.843 | 1.20 | 0.46 | 3.10 | 0.623 |
|  |  |  |  |  |  |  |  |  |  |  |  |  |
| Smoking status (Non-smoker) |  |  |  |  |  |  |  |  |  |  |  |  |
| Ex-smoker | 1.05 | 0.97 | 1.14 | 0.136 | 1.03 | 0.83 | 1.27 | 0.737 | 1.02 | 0.78 | 1.34 | 0.831 |
| Light-smoker | 0.75 | 0.68 | 0.83 | <0.001 | 0.69 | 0.57 | 0.84 | <0.001 | 0.75 | 0.58 | 0.97 | 0.004 |
| Moderate-smoker | 0.60 | 0.43 | 0.83 | <0.001 | 0.77 | 0.43 | 1.38 | 0.248 | 0.27 | 0.09 | 0.78 | 0.002 |
| Heavy-smoker | 0.66 | 0.40 | 1.08 | 0.029 | 0.74 | 0.31 | 1.78 | 0.377 | 0.45 | 0.12 | 1.73 | 0.125 |
|  |  |  |  |  |  |  |  |  |  |  |  |  |
| Alcohol use (Non-drinker/trivial) |  |  |  |  |  |  |  |  |  |  |  |  |
| Light | 1.11 | 1.03 | 1.20 | 0.001 | 1.13 | 0.95 | 1.35 | 0.074 | 1.06 | 0.86 | 1.31 | 0.466 |
| Medium | 1.10 | 0.96 | 1.25 | 0.085 | 1.02 | 0.77 | 1.37 | 0.830 | 0.69 | 0.46 | 1.03 | 0.018 |
| Heavy | 1.01 | 0.74 | 1.39 | 0.923 | 0.64 | 0.34 | 1.23 | 0.080 | 0.45 | 0.16 | 1.27 | 0.047 |
| Very heavy | 0.86 | 0.61 | 1.20 | 0.240 | 0.72 | 0.39 | 1.34 | 0.172 | 0.85 | 0.35 | 2.06 | 0.644 |
|  |  |  |  |  |  |  |  |  |  |  |  |  |
| Ethnic group (White) |  |  |  |  |  |  |  |  |  |  |  |  |
| African/Caribbean | 0.64 | 0.47 | 0.89 | 0.001 | 0.79 | 0.47 | 1.34 | 0.254 | 0.61 | 0.27 | 1.41 | 0.130 |
| Asian | 0.76 | 0.58 | 0.98 | 0.005 | 0.80 | 0.49 | 1.31 | 0.246 | 0.81 | 0.39 | 1.67 | 0.447 |
| Other | 0.79 | 0.63 | 0.99 | 0.007 | 0.59 | 0.35 | 1.01 | 0.012 | 0.55 | 0.26 | 1.20 | 0.048 |
|  |  |  |  |  |  |  |  |  |  |  |  |  |
| Age | 1.01 | 1.00 | 1.01 | <0.001 | 1.01 | 1.00 | 1.02 | <0.001 | 1.01 | 1.00 | 1.02 | 0.013 |
|  |  |  |  |  |  |  |  |  |  |  |  |  |
| Sex (Female) |  |  |  |  |  |  |  |  |  |  |  |  |
| Male | 0.85 | 0.79 | 0.93 | <0.001 | 0.89 | 0.74 | 1.06 | 0.087 | 1.06 | 0.85 | 1.33 | 0.479 |
|  |  |  |  |  |  |  |  |  |  |  |  |  |
| Year of diagnosis (1998-2005) |  |  |  |  |  |  |  |  |  |  |  |  |
| 2006 to 2010 | 1.85 | 0.46 | 7.50 | 0.259 | 1.04 | 0.03 | 38.72 | 0.977 | 1.81 | 0.07 | 45.84 | 0.636 |
| 2011 to 2015 | 1.72 | 0.43 | 6.95 | 0.317 | 1.06 | 0.03 | 39.14 | 0.969 | 1.84 | 0.07 | 46.67 | 0.629 |
| 2016 to 2020 | 1.46 | 0.36 | 6.00 | 0.490 | 1.09 | 0.03 | 40.94 | 0.952 | 1.81 | 0.07 | 46.83 | 0.639 |
|  |  |  |  |  |  |  |  |  |  |  |  |  |
| Coronary heart disease | 0.65 | 0.50 | 0.84 | <0.001 | 0.76 | 0.41 | 1.41 | 0.251 | 0.76 | 0.36 | 1.62 | 0.346 |
| Stroke | 0.90 | 0.68 | 1.20 | 0.348 | 0.91 | 0.50 | 1.65 | 0.678 | 0.67 | 0.26 | 1.70 | 0.266 |
| Diabetes | 0.91 | 0.76 | 1.09 | 0.195 | 0.72 | 0.46 | 1.12 | 0.053 | 0.90 | 0.52 | 1.57 | 0.628 |
| Epilepsy | 1.06 | 0.74 | 1.51 | 0.694 | 0.66 | 0.31 | 1.40 | 0.150 | 0.99 | 0.40 | 2.46 | 0.968 |
| Hypothyroidism | 0.95 | 0.78 | 1.17 | 0.527 | 1.00 | 0.62 | 1.62 | 0.999 | 1.18 | 0.63 | 2.19 | 0.495 |
| Arthritis | 0.98 | 0.84 | 1.13 | 0.693 | 0.95 | 0.67 | 1.33 | 0.671 | 1.08 | 0.71 | 1.66 | 0.622 |
| Anxiety | 0.96 | 0.87 | 1.07 | 0.351 | 1.06 | 0.84 | 1.33 | 0.518 | 0.94 | 0.66 | 1.33 | 0.634 |
| Migraine | 0.94 | 0.81 | 1.08 | 0.224 | 0.99 | 0.73 | 1.33 | 0.924 | 0.85 | 0.56 | 1.28 | 0.300 |
| Cancer | 1.06 | 0.93 | 1.21 | 0.264 | 1.01 | 0.74 | 1.37 | 0.958 | 1.17 | 0.78 | 1.74 | 0.325 |
| Asthma | 0.99 | 0.90 | 1.09 | 0.777 | 0.98 | 0.80 | 1.20 | 0.767 | 1.04 | 0.76 | 1.41 | 0.770 |
| Renal failure | 0.47 | 0.21 | 1.03 | 0.013 | 0.37 | 0.05 | 2.96 | 0.217 | 1.98 | 0.28 | 14.04 | 0.367 |
| Liver failure | 0.88 | 0.58 | 1.35 | 0.450 | 1.09 | 0.50 | 2.35 | 0.776 | 0.92 | 0.26 | 3.34 | 0.874 |
| Osteoporosis | 0.93 | 0.62 | 1.41 | 0.671 | 0.64 | 0.22 | 1.86 | 0.278 | 0.50 | 0.10 | 2.49 | 0.266 |
| Suicidality | 0.82 | 0.65 | 1.04 | 0.031 | 1.11 | 0.72 | 1.72 | 0.530 | 0.64 | 0.32 | 1.27 | 0.095 |
| Antihypertensive | 0.98 | 0.85 | 1.14 | 0.756 | 1.05 | 0.74 | 1.48 | 0.724 | 0.95 | 0.59 | 1.51 | 0.759 |
| Aspirin | 1.11 | 0.89 | 1.38 | 0.232 | 1.01 | 0.59 | 1.71 | 0.974 | 0.75 | 0.38 | 1.50 | 0.288 |
| Anticoagulants | 0.90 | 0.58 | 1.40 | 0.527 | 1.06 | 0.28 | 4.01 | 0.903 | 0.90 | 0.22 | 3.72 | 0.846 |
| NSAIDs | 0.79 | 0.63 | 0.98 | 0.005 | 0.95 | 0.61 | 1.49 | 0.774 | 0.87 | 0.45 | 1.71 | 0.603 |
| Anticonvulsants | 0.71 | 0.49 | 1.03 | 0.019 | 0.87 | 0.36 | 2.12 | 0.695 | 0.78 | 0.26 | 2.35 | 0.568 |
| Hypnotics | 0.96 | 0.84 | 1.09 | 0.394 | 0.76 | 0.57 | 1.01 | 0.013 | 0.99 | 0.68 | 1.44 | 0.930 |
| Bisphosphonates | 1.44 | 0.66 | 3.14 | 0.224 | 1.39 | 0.22 | 8.82 | 0.643 | 1.26 | 0.13 | 12.25 | 0.792 |
| Contraceptives | 1.10 | 0.95 | 1.28 | 0.105 | 1.09 | 0.81 | 1.47 | 0.454 | 1.34 | 0.88 | 2.04 | 0.077 |
| *Constant* | 0.37 | 0.09 | 1.55 | 0.073 | 0.49 | 0.01 | 19.45 | 0.618 | 0.19 | 0.01 | 5.47 | 0.202 |

|  | **Efficacy, response *(FSA, N = 673,177)*** | | | | | | | | | | | |
| --- | --- | --- | --- | --- | --- | --- | --- | --- | --- | --- | --- | --- |
|  | **2 months** | | | | **6 months** | | | | **12 months** | | | |
|  | *OR* | *99% CI* | | *P* | *OR* | *99% CI* | | *P* | *OR* | *99% CI* | | *P* |
| ***Statins*** | 1.01 | 0.94 | 1.09 | 0.715 | 1.01 | 0.95 | 1.06 | 0.723 | 1.00 | 0.96 | 1.04 | 0.992 |
| Antidepressant category (SSRIs) |  |  |  |  |  |  |  |  |  |  |  |  |
| TCAs | 0.95 | 0.92 | 0.99 | 0.001 | 0.98 | 0.94 | 1.03 | 0.307 | 0.99 | 0.94 | 1.04 | 0.550 |
| MAOIs | 0.82 | 0.31 | 2.20 | 0.594 | 0.99 | 0.39 | 2.50 | 0.969 | 0.83 | 0.34 | 2.00 | 0.565 |
| Other antidepressants | 0.97 | 0.94 | 1.01 | 0.027 | 0.99 | 0.93 | 1.04 | 0.481 | 0.99 | 0.94 | 1.03 | 0.398 |
|  |  |  |  |  |  |  |  |  |  |  |  |  |
| PHQ-9 baseline | 1.00 | 0.99 | 1.01 | 0.944 | 1.04 | 1.03 | 1.04 | <0.001 | 1.04 | 1.03 | 1.05 | <0.001 |
| BMI | 1.00 | 0.99 | 1.00 | 0.002 | 0.99 | 0.98 | 1.00 | 0.005 | 0.99 | 0.99 | 1.00 | 0.054 |
|  |  |  |  |  |  |  |  |  |  |  |  |  |
| Read codes for depression (Major depression) |  |  |  |  |  |  |  |  |  |  |  |  |
| Minor depression | 0.98 | 0.96 | 1.00 | 0.009 | 0.99 | 0.97 | 1.01 | 0.068 | 0.99 | 0.96 | 1.02 | 0.427 |
| Other | 0.98 | 0.93 | 1.04 | 0.355 | 0.99 | 0.93 | 1.05 | 0.628 | 0.99 | 0.93 | 1.05 | 0.678 |
|  |  |  |  |  |  |  |  |  |  |  |  |  |
| Townsend deprivation score in fifths (1, least deprived) |  |  |  |  |  |  |  |  |  |  |  |  |
| 2 | 0.90 | 0.85 | 0.96 | <0.001 | 0.87 | 0.78 | 0.97 | 0.003 | 0.86 | 0.70 | 1.05 | 0.039 |
| 3 | 0.78 | 0.74 | 0.82 | <0.001 | 0.76 | 0.70 | 0.81 | <0.001 | 0.73 | 0.65 | 0.83 | <0.001 |
| 4 | 0.69 | 0.66 | 0.73 | <0.001 | 0.67 | 0.60 | 0.75 | <0.001 | 0.69 | 0.59 | 0.81 | <0.001 |
| 5, most deprived | 0.62 | 0.59 | 0.66 | <0.001 | 0.63 | 0.56 | 0.72 | <0.001 | 0.74 | 0.63 | 0.86 | <0.001 |
|  |  |  |  |  |  |  |  |  |  |  |  |  |
| Region of England (East Midlands) |  |  |  |  |  |  |  |  |  |  |  |  |
| East of England | 1.01 | 0.95 | 1.08 | 0.653 | 1.00 | 0.93 | 1.07 | 0.932 | 1.00 | 0.92 | 1.09 | 0.881 |
| London | 1.01 | 0.95 | 1.08 | 0.615 | 1.01 | 0.95 | 1.07 | 0.754 | 1.00 | 0.93 | 1.08 | 0.897 |
| North East | 1.00 | 0.92 | 1.08 | 0.970 | 1.00 | 0.94 | 1.06 | 0.855 | 0.99 | 0.92 | 1.07 | 0.809 |
| North West | 1.00 | 0.95 | 1.06 | 0.905 | 1.00 | 0.95 | 1.05 | 0.920 | 1.00 | 0.93 | 1.07 | 0.964 |
| South Central | 1.03 | 0.96 | 1.09 | 0.287 | 1.00 | 0.94 | 1.07 | 0.875 | 1.01 | 0.93 | 1.09 | 0.755 |
| South East | 1.01 | 0.94 | 1.09 | 0.607 | 1.00 | 0.94 | 1.06 | 0.972 | 1.00 | 0.94 | 1.06 | 0.961 |
| South West | 1.01 | 0.95 | 1.07 | 0.666 | 1.00 | 0.95 | 1.06 | 0.897 | 1.00 | 0.93 | 1.08 | 0.874 |
| West Midlands | 1.02 | 0.96 | 1.08 | 0.505 | 1.00 | 0.94 | 1.06 | 0.947 | 1.00 | 0.93 | 1.08 | 0.859 |
| Yorkshire & Humber | 1.01 | 0.94 | 1.08 | 0.796 | 1.00 | 0.92 | 1.09 | 0.990 | 1.00 | 0.93 | 1.08 | 0.880 |
|  |  |  |  |  |  |  |  |  |  |  |  |  |
| Smoking status (Non-smoker) |  |  |  |  |  |  |  |  |  |  |  |  |
| Ex-smoker | 1.07 | 1.02 | 1.13 | 0.001 | 1.02 | 0.92 | 1.13 | 0.574 | 1.02 | 0.90 | 1.15 | 0.707 |
| Light-smoker | 0.75 | 0.71 | 0.80 | <0.001 | 0.66 | 0.58 | 0.76 | <0.001 | 0.67 | 0.57 | 0.78 | <0.001 |
| Moderate-smoker | 0.67 | 0.54 | 0.82 | <0.001 | 0.69 | 0.49 | 0.96 | 0.006 | 0.57 | 0.35 | 0.92 | 0.004 |
| Heavy-smoker | 0.77 | 0.60 | 0.99 | 0.008 | 0.50 | 0.24 | 1.04 | 0.014 | 0.42 | 0.22 | 0.81 | 0.002 |
|  |  |  |  |  |  |  |  |  |  |  |  |  |
| Alcohol use (Non-drinker/trivial) |  |  |  |  |  |  |  |  |  |  |  |  |
| Light | 1.04 | 0.97 | 1.12 | 0.079 | 1.02 | 0.92 | 1.13 | 0.555 | 1.13 | 1.05 | 1.22 | 0.001 |
| Medium | 1.02 | 0.92 | 1.12 | 0.600 | 0.96 | 0.78 | 1.16 | 0.474 | 1.03 | 0.83 | 1.29 | 0.660 |
| Heavy | 1.00 | 0.85 | 1.17 | 0.947 | 0.86 | 0.56 | 1.31 | 0.282 | 1.00 | 0.64 | 1.58 | 0.974 |
| Very heavy | 0.94 | 0.72 | 1.24 | 0.525 | 0.94 | 0.69 | 1.29 | 0.573 | 1.03 | 0.59 | 1.78 | 0.869 |
|  |  |  |  |  |  |  |  |  |  |  |  |  |
| Ethnic group (White) |  |  |  |  |  |  |  |  |  |  |  |  |
| African/Caribbean | 0.69 | 0.60 | 0.80 | <0.001 | 0.70 | 0.53 | 0.93 | 0.003 | 0.86 | 0.61 | 1.21 | 0.180 |
| Asian | 0.65 | 0.61 | 0.69 | <0.001 | 0.78 | 0.69 | 0.89 | <0.001 | 0.89 | 0.72 | 1.10 | 0.107 |
| Other | 0.70 | 0.62 | 0.78 | <0.001 | 0.72 | 0.59 | 0.87 | <0.001 | 0.76 | 0.63 | 0.93 | 0.001 |
|  |  |  |  |  |  |  |  |  |  |  |  |  |
| Age | 1.00 | 1.00 | 1.00 | 0.006 | 1.00 | 1.00 | 1.00 | 0.104 | 1.00 | 1.00 | 1.00 | 0.154 |
|  |  |  |  |  |  |  |  |  |  |  |  |  |
| Sex (Female) |  |  |  |  |  |  |  |  |  |  |  |  |
| Male | 0.99 | 0.97 | 1.01 | 0.167 | 1.00 | 0.97 | 1.02 | 0.729 | 1.00 | 0.97 | 1.02 | 0.754 |
|  |  |  |  |  |  |  |  |  |  |  |  |  |
| Year of diagnosis (1998-2005) |  |  |  |  |  |  |  |  |  |  |  |  |
| 2006 to 2010 | 1.01 | 0.98 | 1.04 | 0.346 | 1.01 | 0.97 | 1.04 | 0.576 | 1.00 | 0.98 | 1.03 | 0.727 |
| 2011 to 2015 | 1.02 | 0.99 | 1.05 | 0.064 | 1.01 | 0.98 | 1.04 | 0.407 | 1.01 | 0.98 | 1.03 | 0.434 |
| 2016 to 2020 | 0.99 | 0.96 | 1.02 | 0.335 | 1.00 | 0.97 | 1.04 | 0.858 | 1.00 | 0.97 | 1.03 | 0.898 |
|  |  |  |  |  |  |  |  |  |  |  |  |  |
| Coronary heart disease | 0.97 | 0.90 | 1.05 | 0.262 | 0.99 | 0.94 | 1.05 | 0.687 | 0.99 | 0.92 | 1.06 | 0.673 |
| Stroke | 1.00 | 0.92 | 1.08 | 0.906 | 1.01 | 0.94 | 1.08 | 0.750 | 1.00 | 0.91 | 1.10 | 0.930 |
| Diabetes | 1.00 | 0.95 | 1.05 | 0.807 | 0.99 | 0.93 | 1.05 | 0.614 | 1.00 | 0.95 | 1.07 | 0.862 |
| Epilepsy | 1.00 | 0.92 | 1.10 | 0.893 | 1.00 | 0.93 | 1.08 | 0.913 | 1.01 | 0.93 | 1.10 | 0.789 |
| Hypothyroidism | 1.01 | 0.94 | 1.07 | 0.810 | 1.01 | 0.96 | 1.06 | 0.653 | 1.01 | 0.93 | 1.09 | 0.836 |
| Arthritis | 1.00 | 0.96 | 1.04 | 0.982 | 1.00 | 0.96 | 1.04 | 0.983 | 1.00 | 0.96 | 1.05 | 0.997 |
| Anxiety | 1.00 | 0.98 | 1.03 | 0.826 | 1.00 | 0.97 | 1.03 | 0.967 | 0.99 | 0.96 | 1.03 | 0.658 |
| Migraine | 1.00 | 0.96 | 1.04 | 0.898 | 1.00 | 0.95 | 1.04 | 0.806 | 1.00 | 0.96 | 1.04 | 0.876 |
| Cancer | 1.01 | 0.97 | 1.05 | 0.585 | 1.00 | 0.97 | 1.04 | 0.799 | 1.01 | 0.97 | 1.05 | 0.528 |
| Asthma | 1.00 | 0.97 | 1.03 | 0.922 | 1.00 | 0.97 | 1.02 | 0.750 | 1.00 | 0.98 | 1.03 | 0.998 |
| Renal failure | 0.99 | 0.82 | 1.19 | 0.857 | 1.01 | 0.81 | 1.25 | 0.913 | 1.01 | 0.81 | 1.25 | 0.917 |
| Liver failure | 0.97 | 0.87 | 1.08 | 0.424 | 1.00 | 0.88 | 1.13 | 0.954 | 1.00 | 0.89 | 1.13 | 0.953 |
| Osteoporosis | 0.99 | 0.90 | 1.08 | 0.682 | 1.00 | 0.90 | 1.10 | 0.917 | 1.00 | 0.91 | 1.10 | 0.949 |
| Suicidality | 0.98 | 0.92 | 1.05 | 0.482 | 0.99 | 0.94 | 1.04 | 0.574 | 0.98 | 0.92 | 1.05 | 0.465 |
| Antihypertensive | 1.01 | 0.96 | 1.05 | 0.725 | 1.00 | 0.95 | 1.05 | 0.894 | 1.00 | 0.95 | 1.05 | 0.909 |
| Aspirin | 0.99 | 0.91 | 1.07 | 0.753 | 0.99 | 0.93 | 1.05 | 0.601 | 1.00 | 0.94 | 1.06 | 0.833 |
| Anticoagulants | 1.00 | 0.90 | 1.10 | 0.968 | 1.00 | 0.91 | 1.11 | 0.915 | 0.98 | 0.90 | 1.07 | 0.565 |
| NSAIDs | 0.97 | 0.92 | 1.02 | 0.102 | 0.98 | 0.93 | 1.03 | 0.266 | 0.99 | 0.93 | 1.05 | 0.665 |
| Anticonvulsants | 0.97 | 0.87 | 1.07 | 0.371 | 0.99 | 0.91 | 1.07 | 0.731 | 0.99 | 0.92 | 1.07 | 0.742 |
| Hypnotics | 0.99 | 0.94 | 1.03 | 0.359 | 0.99 | 0.96 | 1.03 | 0.522 | 1.00 | 0.96 | 1.04 | 0.792 |
| Bisphosphonates | 1.03 | 0.88 | 1.22 | 0.586 | 0.98 | 0.84 | 1.15 | 0.739 | 0.97 | 0.79 | 1.18 | 0.650 |
| Contraceptives | 1.03 | 0.99 | 1.07 | 0.082 | 1.02 | 0.97 | 1.06 | 0.315 | 1.01 | 0.96 | 1.06 | 0.598 |

### **Table A10i.** Regression analyses for efficacy (remission) at 2 months, 6 months, and 12 months; complete case analysis (CCA) and full set analysis (FSA)

|  | **Efficacy, remission *(CCA)*** | | | | | | | | | | | |
| --- | --- | --- | --- | --- | --- | --- | --- | --- | --- | --- | --- | --- |
|  | **2 months (*N= 22,804)*** | | | | **6 months (*N= 5,025)*** | | | | **12 months (*N= 2,935)*** | | | |
|  | *OR* | *99% CI* | | *P* | *OR* | *99% CI* | | *P* | *OR* | *99% CI* | | *P* |
| ***Statins*** | 1.16 | 0.95 | 1.41 | 0.052 | 0.99 | 0.63 | 1.54 | 0.938 | 1.37 | 0.73 | 2.55 | 0.199 |
| Antidepressant category (SSRIs) |  |  |  |  |  |  |  |  |  |  |  |  |
| TCAs | 0.57 | 0.38 | 0.86 | <0.001 | 0.82 | 0.41 | 1.65 | 0.461 | 0.91 | 0.41 | 2.05 | 0.768 |
| MAOIs |  |  |  |  |  |  |  |  |  |  |  |  |
| Other antidepressants | 0.81 | 0.63 | 1.05 | 0.034 | 0.72 | 0.42 | 1.22 | 0.110 | 1.05 | 0.57 | 1.94 | 0.838 |
|  |  |  |  |  |  |  |  |  |  |  |  |  |
| PHQ-9 baseline | 0.89 | 0.88 | 0.91 | <0.001 | 0.93 | 0.91 | 0.96 | <0.001 | 0.93 | 0.90 | 0.96 | <0.001 |
| BMI | 0.99 | 0.98 | 1.00 | 0.002 | 0.98 | 0.96 | 1.00 | 0.020 | 0.99 | 0.97 | 1.01 | 0.194 |
|  |  |  |  |  |  |  |  |  |  |  |  |  |
| Read codes for depression (Major depression) |  |  |  |  |  |  |  |  |  |  |  |  |
| Minor depression | 0.90 | 0.79 | 1.01 | 0.023 | 0.86 | 0.68 | 1.08 | 0.091 | 1.01 | 0.75 | 1.36 | 0.939 |
| Other | 1.06 | 0.70 | 1.61 | 0.705 | 1.08 | 0.46 | 2.56 | 0.816 | 0.90 | 0.39 | 2.07 | 0.735 |
|  |  |  |  |  |  |  |  |  |  |  |  |  |
| Townsend deprivation score in fifths (1, least deprived) |  |  |  |  |  |  |  |  |  |  |  |  |
| 2 | 0.91 | 0.80 | 1.04 | 0.080 | 0.82 | 0.62 | 1.08 | 0.060 | 1.00 | 0.66 | 1.52 | 0.981 |
| 3 | 0.77 | 0.67 | 0.90 | <0.001 | 0.79 | 0.59 | 1.06 | 0.038 | 0.80 | 0.53 | 1.21 | 0.166 |
| 4 | 0.75 | 0.64 | 0.88 | <0.001 | 0.64 | 0.47 | 0.86 | <0.001 | 0.77 | 0.49 | 1.21 | 0.138 |
| 5, most deprived | 0.70 | 0.58 | 0.84 | <0.001 | 0.65 | 0.46 | 0.92 | 0.001 | 0.86 | 0.51 | 1.46 | 0.470 |
|  |  |  |  |  |  |  |  |  |  |  |  |  |
| Region of England (East Midlands) |  |  |  |  |  |  |  |  |  |  |  |  |
| East of England | 0.91 | 0.47 | 1.74 | 0.695 | 1.09 | 0.53 | 2.23 | 0.764 | 0.65 | 0.25 | 1.72 | 0.253 |
| London | 0.97 | 0.59 | 1.60 | 0.883 | 0.98 | 0.55 | 1.75 | 0.937 | 0.80 | 0.41 | 1.59 | 0.411 |
| North East | 1.06 | 0.62 | 1.82 | 0.773 | 1.22 | 0.60 | 2.48 | 0.460 | 1.04 | 0.35 | 3.10 | 0.931 |
| North West | 0.86 | 0.53 | 1.42 | 0.446 | 0.87 | 0.48 | 1.57 | 0.539 | 0.86 | 0.43 | 1.72 | 0.577 |
| South Central | 1.16 | 0.70 | 1.94 | 0.450 | 1.00 | 0.53 | 1.91 | 0.994 | 0.88 | 0.44 | 1.78 | 0.640 |
| South East | 0.97 | 0.59 | 1.61 | 0.896 | 0.86 | 0.46 | 1.60 | 0.533 | 0.54 | 0.27 | 1.07 | 0.020 |
| South West | 0.98 | 0.59 | 1.64 | 0.925 | 0.73 | 0.37 | 1.41 | 0.214 | 0.55 | 0.25 | 1.20 | 0.048 |
| West Midlands | 0.94 | 0.55 | 1.59 | 0.760 | 1.00 | 0.53 | 1.87 | 0.985 | 0.97 | 0.45 | 2.09 | 0.920 |
| Yorkshire & Humber | 0.79 | 0.46 | 1.35 | 0.255 | 0.91 | 0.46 | 1.78 | 0.705 | 0.77 | 0.31 | 1.92 | 0.468 |
|  |  |  |  |  |  |  |  |  |  |  |  |  |
| Smoking status (Non-smoker) |  |  |  |  |  |  |  |  |  |  |  |  |
| Ex-smoker | 1.10 | 0.99 | 1.23 | 0.022 | 1.00 | 0.78 | 1.29 | 0.984 | 1.16 | 0.83 | 1.62 | 0.254 |
| Light-smoker | 0.84 | 0.74 | 0.94 | <0.001 | 0.73 | 0.57 | 0.94 | 0.001 | 0.76 | 0.54 | 1.07 | 0.038 |
| Moderate-smoker | 0.71 | 0.45 | 1.12 | 0.052 | 0.67 | 0.31 | 1.44 | 0.175 | 0.22 | 0.03 | 1.32 | 0.029 |
| Heavy-smoker | 0.74 | 0.36 | 1.51 | 0.278 | 0.62 | 0.17 | 2.23 | 0.333 | 0.22 | 0.01 | 3.25 | 0.145 |
|  |  |  |  |  |  |  |  |  |  |  |  |  |
| Alcohol use (Non-drinker/trivial) |  |  |  |  |  |  |  |  |  |  |  |  |
| Light | 1.03 | 0.93 | 1.14 | 0.455 | 1.03 | 0.83 | 1.27 | 0.750 | 1.20 | 0.92 | 1.55 | 0.072 |
| Medium | 1.03 | 0.87 | 1.23 | 0.626 | 0.88 | 0.61 | 1.25 | 0.336 | 0.91 | 0.56 | 1.46 | 0.597 |
| Heavy | 0.91 | 0.58 | 1.42 | 0.579 | 0.65 | 0.27 | 1.57 | 0.205 | 0.35 | 0.07 | 1.68 | 0.084 |
| Very heavy | 0.89 | 0.55 | 1.44 | 0.540 | 0.70 | 0.29 | 1.71 | 0.309 | 0.45 | 0.08 | 2.40 | 0.220 |
|  |  |  |  |  |  |  |  |  |  |  |  |  |
| Ethnic group (White) |  |  |  |  |  |  |  |  |  |  |  |  |
| African/Caribbean | 0.73 | 0.46 | 1.16 | 0.079 | 0.52 | 0.21 | 1.28 | 0.061 | 0.96 | 0.31 | 2.98 | 0.917 |
| Asian | 0.63 | 0.44 | 0.92 | 0.002 | 0.71 | 0.39 | 1.29 | 0.139 | 0.94 | 0.38 | 2.33 | 0.871 |
| Other | 0.94 | 0.68 | 1.30 | 0.620 | 0.61 | 0.31 | 1.20 | 0.058 | 0.39 | 0.11 | 1.38 | 0.054 |
|  |  |  |  |  |  |  |  |  |  |  |  |  |
| Age | 1.01 | 1.00 | 1.01 | <0.001 | 1.01 | 1.00 | 1.02 | 0.002 | 1.00 | 0.99 | 1.01 | 0.451 |
|  |  |  |  |  |  |  |  |  |  |  |  |  |
| Sex (Female) |  |  |  |  |  |  |  |  |  |  |  |  |
| Male | 0.87 | 0.79 | 0.97 | 0.001 | 0.95 | 0.77 | 1.18 | 0.566 | 1.01 | 0.78 | 1.32 | 0.915 |
|  |  |  |  |  |  |  |  |  |  |  |  |  |
| Year of diagnosis (1998-2005) |  |  |  |  |  |  |  |  |  |  |  |  |
| 2006 to 2010 | 1.02 | 0.25 | 4.18 | 0.975 | 0.80 | 0.27 | 2.34 | 0.596 | 0.93 | 0.55 | 1.58 | 0.738 |
| 2011 to 2015 | 0.92 | 0.22 | 3.78 | 0.881 | 0.78 | 0.27 | 2.27 | 0.552 | 0.89 | 0.54 | 1.47 | 0.560 |
| 2016 to 2020 | 0.76 | 0.18 | 3.12 | 0.614 | 0.78 | 0.26 | 2.32 | 0.554 | 1.00 |  |  |  |
|  |  |  |  |  |  |  |  |  |  |  |  |  |
| Coronary heart disease | 0.55 | 0.40 | 0.78 | <0.001 | 0.88 | 0.43 | 1.79 | 0.638 | 0.79 | 0.32 | 1.95 | 0.505 |
| Stroke | 0.85 | 0.59 | 1.24 | 0.274 | 0.72 | 0.30 | 1.69 | 0.319 | 0.17 | 0.04 | 0.73 | 0.002 |
| Diabetes | 0.83 | 0.65 | 1.07 | 0.060 | 0.63 | 0.38 | 1.07 | 0.025 | 1.20 | 0.63 | 2.28 | 0.469 |
| Epilepsy | 0.86 | 0.54 | 1.37 | 0.395 | 0.59 | 0.24 | 1.49 | 0.143 | 1.32 | 0.53 | 3.31 | 0.437 |
| Hypothyroidism | 1.03 | 0.81 | 1.31 | 0.730 | 0.92 | 0.52 | 1.61 | 0.692 | 1.45 | 0.72 | 2.92 | 0.173 |
| Arthritis | 1.04 | 0.87 | 1.25 | 0.582 | 1.20 | 0.80 | 1.79 | 0.255 | 0.94 | 0.53 | 1.68 | 0.800 |
| Anxiety | 0.93 | 0.81 | 1.07 | 0.202 | 1.10 | 0.85 | 1.43 | 0.351 | 0.93 | 0.63 | 1.37 | 0.626 |
| Migraine | 0.80 | 0.66 | 0.98 | 0.004 | 0.89 | 0.62 | 1.28 | 0.414 | 0.83 | 0.50 | 1.37 | 0.344 |
| Cancer | 1.00 | 0.84 | 1.18 | 0.961 | 0.97 | 0.67 | 1.41 | 0.832 | 1.16 | 0.67 | 2.01 | 0.490 |
| Asthma | 0.99 | 0.88 | 1.12 | 0.895 | 0.95 | 0.74 | 1.22 | 0.596 | 1.11 | 0.76 | 1.62 | 0.466 |
| Renal failure | 0.55 | 0.19 | 1.63 | 0.157 | 1.00 |  |  |  | 0.46 | 0.02 | 10.01 | 0.513 |
| Liver failure | 0.80 | 0.46 | 1.40 | 0.310 | 1.32 | 0.51 | 3.38 | 0.448 | 0.91 | 0.17 | 5.01 | 0.889 |
| Osteoporosis | 1.15 | 0.72 | 1.83 | 0.451 | 0.84 | 0.24 | 2.96 | 0.720 | 0.70 | 0.13 | 3.71 | 0.578 |
| Suicidality | 0.79 | 0.58 | 1.09 | 0.060 | 1.04 | 0.60 | 1.82 | 0.842 | 0.58 | 0.22 | 1.57 | 0.163 |
| Antihypertensive | 0.96 | 0.80 | 1.14 | 0.533 | 1.17 | 0.78 | 1.77 | 0.314 | 0.63 | 0.32 | 1.21 | 0.068 |
| Aspirin | 1.11 | 0.83 | 1.49 | 0.336 | 0.95 | 0.53 | 1.73 | 0.840 | 1.58 | 0.71 | 3.50 | 0.138 |
| Anticoagulants | 0.85 | 0.49 | 1.47 | 0.456 | 1.28 | 0.36 | 4.61 | 0.616 | 1.27 | 0.21 | 7.61 | 0.728 |
| NSAIDs | 0.82 | 0.60 | 1.12 | 0.100 | 0.99 | 0.56 | 1.73 | 0.955 | 1.21 | 0.55 | 2.70 | 0.532 |
| Anticonvulsants | 0.77 | 0.47 | 1.28 | 0.188 | 1.30 | 0.45 | 3.72 | 0.523 | 0.82 | 0.26 | 2.62 | 0.661 |
| Hypnotics | 0.98 | 0.82 | 1.17 | 0.796 | 0.81 | 0.56 | 1.18 | 0.150 | 1.06 | 0.67 | 1.67 | 0.758 |
| Bisphosphonates | 0.85 | 0.34 | 2.13 | 0.644 | 1.77 | 0.27 | 11.81 | 0.437 | 2.53 | 0.38 | 17.03 | 0.209 |
| Contraceptives | 1.08 | 0.90 | 1.30 | 0.289 | 1.03 | 0.70 | 1.51 | 0.864 | 1.05 | 0.60 | 1.81 | 0.836 |
| *Constant* | 1.79 | 0.40 | 8.00 | 0.315 | 1.74 | 0.48 | 6.29 | 0.270 | 1.23 | 0.41 | 3.71 | 0.635 |

|  | **Efficacy, remission *(FSA, N = 673,177)*** | | | | | | | | | | | |
| --- | --- | --- | --- | --- | --- | --- | --- | --- | --- | --- | --- | --- |
|  | **2 months** | | | | **6 months** | | | | **12 months** | | | |
|  | *OR* | *99% CI* | | *P* | *OR* | *99% CI* | | *P* | *OR* | *99% CI* | | *P* |
| ***Statins*** | 1.02 | 0.95 | 1.08 | 0.502 | 1.01 | 0.95 | 1.08 | 0.541 | 1.00 | 0.92 | 1.08 | 0.973 |
| Antidepressant category (SSRIs) |  |  |  |  |  |  |  |  |  |  |  |  |
| TCAs | 0.95 | 0.89 | 1.02 | 0.060 | 0.99 | 0.93 | 1.05 | 0.532 | 0.98 | 0.93 | 1.04 | 0.451 |
| MAOIs | 0.81 | 0.17 | 3.78 | 0.703 | 1.01 | 0.30 | 3.41 | 0.975 | 0.77 | 0.17 | 3.58 | 0.650 |
| Other antidepressants | 0.97 | 0.92 | 1.03 | 0.178 | 0.99 | 0.94 | 1.04 | 0.559 | 0.99 | 0.93 | 1.04 | 0.521 |
|  |  |  |  |  |  |  |  |  |  |  |  |  |
| PHQ-9 baseline | 0.87 | 0.86 | 0.87 | <0.001 | 0.91 | 0.90 | 0.92 | <0.001 | 0.92 | 0.91 | 0.93 | <0.001 |
| BMI | 1.00 | 0.99 | 1.00 | 0.011 | 0.99 | 0.98 | 1.00 | 0.004 | 0.99 | 0.99 | 1.00 | 0.073 |
|  |  |  |  |  |  |  |  |  |  |  |  |  |
| Read codes for depression (Major depression) |  |  |  |  |  |  |  |  |  |  |  |  |
| Minor depression | 0.95 | 0.93 | 0.98 | <0.001 | 0.99 | 0.95 | 1.02 | 0.281 | 0.99 | 0.94 | 1.04 | 0.481 |
| Other | 0.99 | 0.91 | 1.07 | 0.650 | 1.00 | 0.91 | 1.09 | 0.984 | 1.00 | 0.92 | 1.08 | 0.933 |
|  |  |  |  |  |  |  |  |  |  |  |  |  |
| Townsend deprivation score in fifths (1, least deprived) |  |  |  |  |  |  |  |  |  |  |  |  |
| 2 | 0.90 | 0.83 | 0.98 | 0.002 | 0.88 | 0.79 | 0.98 | 0.004 | 0.87 | 0.69 | 1.08 | 0.068 |
| 3 | 0.78 | 0.74 | 0.82 | <0.001 | 0.76 | 0.71 | 0.82 | <0.001 | 0.73 | 0.64 | 0.85 | <0.001 |
| 4 | 0.70 | 0.65 | 0.74 | <0.001 | 0.67 | 0.60 | 0.75 | <0.001 | 0.69 | 0.57 | 0.84 | <0.001 |
| 5, most deprived | 0.62 | 0.58 | 0.67 | <0.001 | 0.63 | 0.56 | 0.71 | <0.001 | 0.74 | 0.62 | 0.88 | <0.001 |
|  |  |  |  |  |  |  |  |  |  |  |  |  |
| Region of England (East Midlands) |  |  |  |  |  |  |  |  |  |  |  |  |
| East of England | 1.04 | 0.93 | 1.16 | 0.323 | 0.99 | 0.89 | 1.11 | 0.853 | 1.00 | 0.90 | 1.10 | 0.991 |
| London | 1.04 | 0.95 | 1.14 | 0.231 | 1.02 | 0.93 | 1.11 | 0.606 | 1.01 | 0.93 | 1.10 | 0.690 |
| North East | 1.03 | 0.92 | 1.15 | 0.455 | 1.01 | 0.90 | 1.13 | 0.815 | 1.00 | 0.87 | 1.16 | 0.940 |
| North West | 1.02 | 0.94 | 1.10 | 0.520 | 1.01 | 0.92 | 1.10 | 0.841 | 1.00 | 0.92 | 1.09 | 0.939 |
| South Central | 1.05 | 0.96 | 1.16 | 0.142 | 1.01 | 0.92 | 1.11 | 0.792 | 1.01 | 0.92 | 1.11 | 0.728 |
| South East | 1.03 | 0.94 | 1.14 | 0.364 | 1.00 | 0.91 | 1.10 | 0.917 | 1.00 | 0.92 | 1.08 | 0.977 |
| South West | 1.03 | 0.93 | 1.14 | 0.473 | 1.00 | 0.91 | 1.10 | 0.987 | 1.01 | 0.92 | 1.11 | 0.792 |
| West Midlands | 1.04 | 0.96 | 1.12 | 0.250 | 1.01 | 0.92 | 1.11 | 0.824 | 1.01 | 0.92 | 1.11 | 0.808 |
| Yorkshire & Humber | 1.01 | 0.90 | 1.13 | 0.814 | 1.01 | 0.88 | 1.14 | 0.908 | 1.01 | 0.91 | 1.11 | 0.888 |
|  |  |  |  |  |  |  |  |  |  |  |  |  |
| Smoking status (Non-smoker) |  |  |  |  |  |  |  |  |  |  |  |  |
| Ex-smoker | 1.08 | 1.01 | 1.15 | 0.003 | 1.02 | 0.91 | 1.13 | 0.653 | 1.02 | 0.88 | 1.17 | 0.727 |
| Light-smoker | 0.75 | 0.71 | 0.80 | <0.001 | 0.66 | 0.57 | 0.76 | <0.001 | 0.66 | 0.56 | 0.78 | <0.001 |
| Moderate-smoker | 0.66 | 0.53 | 0.82 | <0.001 | 0.69 | 0.49 | 0.99 | 0.009 | 0.56 | 0.33 | 0.95 | 0.006 |
| Heavy-smoker | 0.76 | 0.56 | 1.05 | 0.026 | 0.47 | 0.19 | 1.13 | 0.021 | 0.38 | 0.17 | 0.87 | 0.004 |
|  |  |  |  |  |  |  |  |  |  |  |  |  |
| Alcohol use (Non-drinker/trivial) |  |  |  |  |  |  |  |  |  |  |  |  |
| Light | 1.04 | 0.97 | 1.11 | 0.108 | 1.02 | 0.92 | 1.14 | 0.547 | 1.14 | 1.05 | 1.24 | 0.001 |
| Medium | 1.01 | 0.90 | 1.13 | 0.846 | 0.95 | 0.77 | 1.18 | 0.486 | 1.04 | 0.83 | 1.30 | 0.635 |
| Heavy | 0.97 | 0.82 | 1.15 | 0.659 | 0.85 | 0.53 | 1.34 | 0.277 | 1.00 | 0.61 | 1.65 | 0.986 |
| Very heavy | 0.94 | 0.67 | 1.30 | 0.551 | 0.94 | 0.65 | 1.36 | 0.614 | 1.01 | 0.55 | 1.86 | 0.945 |
|  |  |  |  |  |  |  |  |  |  |  |  |  |
| Ethnic group (White) |  |  |  |  |  |  |  |  |  |  |  |  |
| African/Caribbean | 0.68 | 0.58 | 0.79 | <0.001 | 0.69 | 0.52 | 0.93 | 0.003 | 0.86 | 0.59 | 1.24 | 0.211 |
| Asian | 0.64 | 0.59 | 0.71 | <0.001 | 0.78 | 0.67 | 0.91 | <0.001 | 0.90 | 0.74 | 1.10 | 0.131 |
| Other | 0.71 | 0.63 | 0.80 | <0.001 | 0.71 | 0.56 | 0.91 | 0.001 | 0.76 | 0.60 | 0.96 | 0.004 |
|  |  |  |  |  |  |  |  |  |  |  |  |  |
| Age | 1.00 | 1.00 | 1.00 | 0.035 | 1.00 | 1.00 | 1.00 | 0.118 | 1.00 | 1.00 | 1.00 | 0.360 |
|  |  |  |  |  |  |  |  |  |  |  |  |  |
| Sex (Female) |  |  |  |  |  |  |  |  |  |  |  |  |
| Male | 0.99 | 0.96 | 1.02 | 0.438 | 0.99 | 0.97 | 1.02 | 0.385 | 0.99 | 0.96 | 1.03 | 0.639 |
|  |  |  |  |  |  |  |  |  |  |  |  |  |
| Year of diagnosis (1998-2005) |  |  |  |  |  |  |  |  |  |  |  |  |
| 2006 to 2010 | 1.08 | 1.03 | 1.13 | <0.001 | 1.02 | 0.99 | 1.07 | 0.101 | 1.02 | 0.98 | 1.07 | 0.184 |
| 2011 to 2015 | 1.08 | 1.03 | 1.13 | <0.001 | 1.02 | 0.98 | 1.06 | 0.175 | 1.02 | 0.97 | 1.06 | 0.341 |
| 2016 to 2020 | 1.01 | 0.96 | 1.05 | 0.743 | 1.01 | 0.97 | 1.05 | 0.642 | 1.01 | 0.97 | 1.05 | 0.609 |
|  |  |  |  |  |  |  |  |  |  |  |  |  |
| Coronary heart disease | 0.96 | 0.88 | 1.05 | 0.206 | 0.98 | 0.88 | 1.10 | 0.680 | 0.99 | 0.90 | 1.08 | 0.727 |
| Stroke | 0.98 | 0.89 | 1.08 | 0.607 | 1.00 | 0.91 | 1.10 | 0.967 | 0.99 | 0.91 | 1.08 | 0.806 |
| Diabetes | 0.98 | 0.92 | 1.05 | 0.482 | 0.99 | 0.91 | 1.07 | 0.681 | 1.01 | 0.95 | 1.08 | 0.651 |
| Epilepsy | 1.01 | 0.88 | 1.16 | 0.885 | 1.01 | 0.90 | 1.13 | 0.863 | 1.00 | 0.88 | 1.13 | 0.980 |
| Hypothyroidism | 1.00 | 0.92 | 1.09 | 0.885 | 1.01 | 0.91 | 1.11 | 0.822 | 1.02 | 0.91 | 1.14 | 0.642 |
| Arthritis | 1.01 | 0.94 | 1.08 | 0.786 | 1.00 | 0.93 | 1.07 | 0.959 | 0.99 | 0.92 | 1.07 | 0.851 |
| Anxiety | 1.00 | 0.95 | 1.06 | 0.879 | 1.00 | 0.97 | 1.04 | 0.840 | 0.99 | 0.95 | 1.03 | 0.641 |
| Migraine | 1.00 | 0.95 | 1.06 | 0.951 | 0.99 | 0.93 | 1.06 | 0.802 | 1.00 | 0.94 | 1.06 | 0.869 |
| Cancer | 1.01 | 0.95 | 1.06 | 0.782 | 1.00 | 0.95 | 1.05 | 0.892 | 1.01 | 0.96 | 1.07 | 0.630 |
| Asthma | 1.00 | 0.96 | 1.05 | 0.838 | 1.00 | 0.97 | 1.03 | 0.965 | 1.00 | 0.97 | 1.04 | 0.931 |
| Renal failure | 0.98 | 0.78 | 1.24 | 0.840 | 0.98 | 0.73 | 1.32 | 0.876 | 1.01 | 0.74 | 1.38 | 0.936 |
| Liver failure | 0.96 | 0.82 | 1.12 | 0.518 | 1.00 | 0.84 | 1.19 | 0.990 | 1.00 | 0.80 | 1.26 | 0.998 |
| Osteoporosis | 0.99 | 0.85 | 1.16 | 0.914 | 0.98 | 0.88 | 1.10 | 0.691 | 1.01 | 0.89 | 1.14 | 0.884 |
| Suicidality | 0.99 | 0.87 | 1.12 | 0.817 | 0.99 | 0.92 | 1.08 | 0.856 | 0.99 | 0.91 | 1.07 | 0.695 |
| Antihypertensive | 1.00 | 0.93 | 1.08 | 0.979 | 1.00 | 0.95 | 1.06 | 0.848 | 1.00 | 0.93 | 1.07 | 0.946 |
| Aspirin | 0.99 | 0.91 | 1.06 | 0.624 | 0.99 | 0.92 | 1.07 | 0.663 | 1.00 | 0.91 | 1.09 | 0.880 |
| Anticoagulants | 0.99 | 0.86 | 1.14 | 0.818 | 1.01 | 0.89 | 1.15 | 0.843 | 0.98 | 0.86 | 1.11 | 0.649 |
| NSAIDs | 0.97 | 0.89 | 1.07 | 0.416 | 0.98 | 0.91 | 1.05 | 0.363 | 0.99 | 0.90 | 1.08 | 0.757 |
| Anticonvulsants | 0.97 | 0.86 | 1.10 | 0.518 | 0.99 | 0.87 | 1.12 | 0.783 | 0.99 | 0.88 | 1.10 | 0.761 |
| Hypnotics | 0.98 | 0.93 | 1.02 | 0.190 | 0.99 | 0.94 | 1.05 | 0.680 | 1.00 | 0.94 | 1.05 | 0.879 |
| Bisphosphonates | 1.01 | 0.79 | 1.28 | 0.951 | 0.98 | 0.74 | 1.28 | 0.805 | 0.99 | 0.75 | 1.32 | 0.955 |
| Contraceptives | 1.02 | 0.95 | 1.10 | 0.409 | 1.01 | 0.94 | 1.08 | 0.768 | 1.01 | 0.92 | 1.11 | 0.734 |
| *Constant* | 1.77 | 1.57 | 2.00 | <0.001 | 1.28 | 0.94 | 1.74 | 0.031 | 0.83 | 0.61 | 1.14 | 0.094 |

### **Table A10j.** Regression analyses for efficacy (change from baseline) at 2 months, 6 months, and 12 months; complete case analysis (CCA) and full set analysis (FSA)

|  | **Efficacy, change from baseline *(CCA)*** | | | | | | | | | | | |
| --- | --- | --- | --- | --- | --- | --- | --- | --- | --- | --- | --- | --- |
|  | **2 months (*N= 22,804)*** | | | | **6 months (*N= 5,035)*** | | | | **12 months (*N= 2,938)*** | | | |
|  | *MD* | *99% CI* | | *P* | *MD* | *99% CI* | | *P* | *MD* | *99% CI* | | *P* |
| ***Statins*** | -0.11 | -0.54 | 0.32 | 0.509 | -0.02 | -1.10 | 1.06 | 0.962 | -1.31 | -2.92 | 0.30 | 0.035 |
| Antidepressant category (SSRIs) |  |  |  |  |  |  |  |  |  |  |  |  |
| TCAs | 1.69 | 0.94 | 2.43 | <0.001 | 0.80 | -0.70 | 2.31 | 0.169 | 1.10 | -0.69 | 2.90 | 0.113 |
| MAOIs |  |  |  |  |  |  |  |  |  |  |  |  |
| Other antidepressants | 0.50 | 0.04 | 0.95 | 0.005 | 0.71 | -0.42 | 1.84 | 0.105 | 0.61 | -0.89 | 2.10 | 0.295 |
|  |  |  |  |  |  |  |  |  |  |  |  |  |
| PHQ-9 baseline | -0.53 | -0.55 | -0.50 | <0.001 | -0.64 | -0.70 | -0.59 | <0.001 | -0.67 | -0.74 | -0.60 | <0.001 |
| BMI | 0.03 | 0.01 | 0.05 | <0.001 | 0.06 | 0.02 | 0.10 | <0.001 | 0.04 | -0.02 | 0.10 | 0.054 |
|  |  |  |  |  |  |  |  |  |  |  |  |  |
| Read codes for depression (Major depression) |  |  |  |  |  |  |  |  |  |  |  |  |
| Minor depression | 0.30 | 0.03 | 0.57 | 0.004 | 0.46 | -0.09 | 1.02 | 0.031 | 0.34 | -0.44 | 1.11 | 0.259 |
| Other | 0.58 | -0.33 | 1.48 | 0.099 | 1.09 | -1.10 | 3.27 | 0.198 | 0.49 | -1.47 | 2.45 | 0.516 |
|  |  |  |  |  |  |  |  |  |  |  |  |  |
| Townsend deprivation score in fifths (1, least deprived) |  |  |  |  |  |  |  |  |  |  |  |  |
| 2 | 0.38 | 0.09 | 0.66 | 0.001 | 0.71 | -0.04 | 1.47 | 0.015 | 1.03 | 0.03 | 2.03 | 0.008 |
| 3 | 0.86 | 0.56 | 1.16 | <0.001 | 0.68 | -0.07 | 1.43 | 0.020 | 1.47 | 0.52 | 2.43 | <0.001 |
| 4 | 1.08 | 0.73 | 1.42 | <0.001 | 1.38 | 0.62 | 2.15 | <0.001 | 1.71 | 0.67 | 2.75 | <0.001 |
| 5, most deprived | 1.32 | 0.93 | 1.71 | <0.001 | 1.35 | 0.50 | 2.20 | <0.001 | 1.28 | 0.07 | 2.49 | 0.006 |
|  |  |  |  |  |  |  |  |  |  |  |  |  |
| Region of England (East Midlands) |  |  |  |  |  |  |  |  |  |  |  |  |
| East of England | 0.15 | -1.25 | 1.54 | 0.785 | -0.06 | -2.09 | 1.97 | 0.940 | 0.46 | -2.09 | 3.01 | 0.644 |
| London | -0.15 | -1.13 | 0.83 | 0.692 | -0.39 | -1.94 | 1.16 | 0.514 | 0.16 | -1.83 | 2.16 | 0.833 |
| North East | 0.23 | -0.94 | 1.40 | 0.612 | 0.67 | -1.26 | 2.59 | 0.372 | 0.90 | -2.50 | 4.30 | 0.494 |
| North West | 0.28 | -0.71 | 1.28 | 0.463 | 0.00 | -1.59 | 1.59 | 1.000 | 0.35 | -1.63 | 2.34 | 0.644 |
| South Central | -0.49 | -1.48 | 0.51 | 0.205 | -0.52 | -2.18 | 1.15 | 0.423 | 0.13 | -1.91 | 2.17 | 0.869 |
| South East | -0.17 | -1.16 | 0.82 | 0.662 | 0.03 | -1.68 | 1.74 | 0.963 | 0.79 | -1.15 | 2.72 | 0.295 |
| South West | -0.25 | -1.23 | 0.74 | 0.519 | 0.49 | -1.23 | 2.20 | 0.465 | 1.23 | -0.95 | 3.41 | 0.144 |
| West Midlands | 0.01 | -1.02 | 1.05 | 0.972 | -0.15 | -1.75 | 1.45 | 0.811 | -0.29 | -2.38 | 1.80 | 0.723 |
| Yorkshire & Humber | 0.15 | -0.92 | 1.22 | 0.712 | -0.33 | -2.08 | 1.43 | 0.631 | -0.58 | -3.38 | 2.21 | 0.591 |
|  |  |  |  |  |  |  |  |  |  |  |  |  |
| Smoking status (Non-smoker) |  |  |  |  |  |  |  |  |  |  |  |  |
| Ex-smoker | -0.12 | -0.35 | 0.12 | 0.206 | -0.21 | -0.84 | 0.43 | 0.400 | -0.04 | -0.84 | 0.76 | 0.903 |
| Light-smoker | 0.96 | 0.70 | 1.22 | <0.001 | 1.43 | 0.86 | 2.01 | <0.001 | 1.12 | 0.32 | 1.91 | <0.001 |
| Moderate-smoker | 1.43 | 0.60 | 2.27 | <0.001 | 0.97 | -0.87 | 2.81 | 0.175 | 2.87 | 0.67 | 5.06 | 0.001 |
| Heavy-smoker | 1.02 | -0.12 | 2.17 | 0.021 | 2.01 | -0.58 | 4.59 | 0.045 | 4.05 | 0.58 | 7.52 | 0.003 |
|  |  |  |  |  |  |  |  |  |  |  |  |  |
| Alcohol use (Non-drinker/trivial) |  |  |  |  |  |  |  |  |  |  |  |  |
| Light | -0.27 | -0.48 | -0.07 | 0.001 | -0.30 | -0.79 | 0.19 | 0.112 | -0.32 | -0.98 | 0.34 | 0.213 |
| Medium | -0.34 | -0.71 | 0.02 | 0.016 | 0.02 | -0.85 | 0.90 | 0.942 | 0.74 | -0.34 | 1.83 | 0.075 |
| Heavy | 0.18 | -0.71 | 1.07 | 0.605 | 1.14 | -0.71 | 3.00 | 0.112 | 2.37 | -0.14 | 4.87 | 0.015 |
| Very heavy | 0.64 | -0.25 | 1.52 | 0.063 | 1.20 | -0.61 | 3.02 | 0.088 | 2.33 | -0.60 | 5.27 | 0.040 |
|  |  |  |  |  |  |  |  |  |  |  |  |  |
| Ethnic group (White) |  |  |  |  |  |  |  |  |  |  |  |  |
| African/Caribbean | 1.51 | 0.66 | 2.35 | <0.001 | 1.33 | -0.18 | 2.84 | 0.023 | 1.41 | -1.10 | 3.92 | 0.148 |
| Asian | 1.11 | 0.40 | 1.81 | <0.001 | 1.08 | -0.28 | 2.45 | 0.041 | 1.05 | -1.13 | 3.23 | 0.213 |
| Other | 0.96 | 0.37 | 1.54 | <0.001 | 1.71 | 0.36 | 3.05 | 0.001 | 1.75 | -0.24 | 3.73 | 0.023 |
|  |  |  |  |  |  |  |  |  |  |  |  |  |
| Age | -0.02 | -0.03 | -0.01 | <0.001 | -0.04 | -0.06 | -0.02 | <0.001 | -0.03 | -0.05 | 0.00 | 0.014 |
|  |  |  |  |  |  |  |  |  |  |  |  |  |
| Sex (Female) |  |  |  |  |  |  |  |  |  |  |  |  |
| Male | 0.55 | 0.34 | 0.76 | <0.001 | 0.42 | -0.11 | 0.94 | 0.040 | -0.05 | -0.69 | 0.58 | 0.826 |
|  |  |  |  |  |  |  |  |  |  |  |  |  |
| Year of diagnosis (1998-2005) |  |  |  |  |  |  |  |  |  |  |  |  |
| 2006 to 2010 | -0.83 | -3.64 | 1.98 | 0.447 | 3.66 | 0.09 | 7.23 | 0.008 | -4.64 | -12.85 | 3.57 | 0.145 |
| 2011 to 2015 | -0.75 | -3.55 | 2.06 | 0.492 | 3.64 | 0.09 | 7.18 | 0.008 | -4.81 | -12.99 | 3.37 | 0.129 |
| 2016 to 2020 | -0.06 | -2.89 | 2.76 | 0.955 | 3.68 | 0.07 | 7.29 | 0.009 | -4.39 | -12.66 | 3.87 | 0.170 |
|  |  |  |  |  |  |  |  |  |  |  |  |  |
| Coronary heart disease | 0.99 | 0.31 | 1.67 | <0.001 | 0.30 | -1.42 | 2.01 | 0.655 | 0.45 | -1.77 | 2.68 | 0.598 |
| Stroke | 0.10 | -0.64 | 0.84 | 0.731 | 0.32 | -1.38 | 2.01 | 0.630 | 1.84 | -0.56 | 4.25 | 0.048 |
| Diabetes | 0.21 | -0.26 | 0.68 | 0.242 | 0.76 | -0.48 | 2.00 | 0.113 | 0.79 | -1.02 | 2.60 | 0.258 |
| Epilepsy | 0.34 | -0.63 | 1.31 | 0.368 | 2.00 | -0.35 | 4.36 | 0.029 | -0.15 | -2.71 | 2.42 | 0.883 |
| Hypothyroidism | 0.01 | -0.50 | 0.53 | 0.950 | 0.16 | -1.20 | 1.51 | 0.766 | -1.23 | -3.02 | 0.56 | 0.076 |
| Arthritis | -0.04 | -0.43 | 0.36 | 0.802 | 0.21 | -0.79 | 1.21 | 0.588 | -0.24 | -1.57 | 1.09 | 0.640 |
| Anxiety | 0.22 | -0.06 | 0.50 | 0.043 | -0.16 | -0.83 | 0.50 | 0.525 | 0.20 | -0.89 | 1.29 | 0.639 |
| Migraine | 0.40 | 0.01 | 0.79 | 0.008 | 0.27 | -0.62 | 1.16 | 0.431 | 0.22 | -0.99 | 1.44 | 0.635 |
| Cancer | -0.33 | -0.68 | 0.02 | 0.016 | -0.06 | -1.00 | 0.88 | 0.877 | -0.52 | -1.75 | 0.71 | 0.275 |
| Asthma | 0.02 | -0.23 | 0.28 | 0.818 | 0.19 | -0.40 | 0.77 | 0.407 | 0.10 | -0.80 | 1.00 | 0.774 |
| Renal failure | 1.24 | -0.39 | 2.86 | 0.050 | 5.49 | 1.19 | 9.80 | 0.001 | -2.51 | -7.79 | 2.76 | 0.219 |
| Liver failure | 0.12 | -0.99 | 1.23 | 0.779 | -0.42 | -2.75 | 1.91 | 0.639 | -0.15 | -3.81 | 3.50 | 0.914 |
| Osteoporosis | -0.35 | -1.40 | 0.69 | 0.381 | 0.72 | -2.10 | 3.54 | 0.508 | 1.37 | -2.27 | 5.00 | 0.332 |
| Suicidality | 0.72 | 0.13 | 1.31 | 0.002 | 0.35 | -1.16 | 1.86 | 0.550 | 1.70 | -0.31 | 3.70 | 0.029 |
| Antihypertensive | -0.11 | -0.48 | 0.26 | 0.452 | -0.36 | -1.44 | 0.72 | 0.392 | 0.28 | -1.11 | 1.66 | 0.606 |
| Aspirin | 0.03 | -0.57 | 0.64 | 0.890 | 0.49 | -1.00 | 1.99 | 0.394 | 1.31 | -0.66 | 3.27 | 0.086 |
| Anticoagulants | 0.37 | -0.72 | 1.46 | 0.384 | 0.57 | -3.35 | 4.48 | 0.708 | -0.12 | -4.11 | 3.88 | 0.940 |
| NSAIDs | 0.95 | 0.39 | 1.51 | <0.001 | 0.20 | -1.20 | 1.59 | 0.715 | 0.30 | -1.79 | 2.38 | 0.711 |
| Anticonvulsants | 0.91 | -0.02 | 1.85 | 0.012 | -0.12 | -2.87 | 2.63 | 0.907 | 0.96 | -2.24 | 4.15 | 0.439 |
| Hypnotics | 0.20 | -0.16 | 0.56 | 0.145 | 0.57 | -0.27 | 1.41 | 0.079 | -0.30 | -1.47 | 0.86 | 0.500 |
| Bisphosphonates | 0.52 | -1.52 | 2.57 | 0.509 | -0.89 | -4.21 | 2.43 | 0.489 | -2.06 | -5.52 | 1.41 | 0.126 |
| Contraceptives | -0.53 | -0.94 | -0.13 | 0.001 | -0.23 | -1.11 | 0.64 | 0.493 | -0.43 | -1.74 | 0.87 | 0.391 |
| *Constant* | 2.36 | -0.61 | 5.34 | 0.041 | -0.20 | -4.19 | 3.79 | 0.895 | 8.87 | 0.42 | 17.31 | 0.007 |

|  | **Efficacy, change from baseline *(FSA, N= 673,177)*** | | | | | | | | | | | |
| --- | --- | --- | --- | --- | --- | --- | --- | --- | --- | --- | --- | --- |
|  | **2 months** | | | | **6 months** | | | | **12 months** | | | |
|  | *MD* | *99% CI* | | *P* | *MD* | *99% CI* | | *P* | *MD* | *99% CI* | | *P* |
| ***Statins*** | -0.01 | -0.16 | 0.14 | 0.846 | -0.02 | -0.20 | 0.16 | 0.748 | -0.01 | -0.12 | 0.11 | 0.855 |
| Antidepressant category (SSRIs) |  |  |  |  |  |  |  |  |  |  |  |  |
| TCAs | 0.18 | 0.07 | 0.30 | <0.001 | 0.07 | -0.07 | 0.22 | 0.172 | 0.05 | -0.08 | 0.17 | 0.342 |
| MAOIs | 0.54 | -1.54 | 2.62 | 0.495 | 0.29 | -1.99 | 2.56 | 0.739 | 0.47 | -2.30 | 3.24 | 0.645 |
| Other antidepressants | 0.10 | -0.01 | 0.20 | 0.015 | 0.05 | -0.08 | 0.19 | 0.298 | 0.05 | -0.09 | 0.20 | 0.297 |
|  |  |  |  |  |  |  |  |  |  |  |  |  |
| PHQ-9 baseline | -0.50 | -0.52 | -0.48 | <0.001 | -0.65 | -0.68 | -0.61 | <0.001 | -0.67 | -0.71 | -0.63 | <0.001 |
| BMI | 0.02 | 0.01 | 0.03 | 0.001 | 0.03 | 0.00 | 0.07 | 0.008 | 0.02 | -0.01 | 0.06 | 0.053 |
|  |  |  |  |  |  |  |  |  |  |  |  |  |
| Read codes for depression (Major depression) |  |  |  |  |  |  |  |  |  |  |  |  |
| Minor depression | 0.07 | 0.01 | 0.12 | 0.001 | 0.05 | 0.00 | 0.10 | 0.015 | 0.03 | -0.03 | 0.09 | 0.158 |
| Other | 0.03 | -0.11 | 0.17 | 0.576 | 0.02 | -0.15 | 0.18 | 0.784 | 0.03 | -0.15 | 0.20 | 0.676 |
|  |  |  |  |  |  |  |  |  |  |  |  |  |
| Townsend deprivation score in fifths (1, least deprived) |  |  |  |  |  |  |  |  |  |  |  |  |
| 2 | 0.38 | 0.12 | 0.64 | 0.001 | 0.52 | 0.09 | 0.94 | 0.004 | 0.58 | -0.45 | 1.62 | 0.073 |
| 3 | 0.88 | 0.72 | 1.04 | <0.001 | 1.06 | 0.76 | 1.37 | <0.001 | 1.20 | 0.67 | 1.73 | <0.001 |
| 4 | 1.28 | 1.14 | 1.42 | <0.001 | 1.52 | 1.07 | 1.96 | <0.001 | 1.40 | 0.71 | 2.09 | <0.001 |
| 5, most deprived | 1.64 | 1.45 | 1.83 | <0.001 | 1.73 | 1.22 | 2.23 | <0.001 | 1.19 | 0.55 | 1.82 | <0.001 |
|  |  |  |  |  |  |  |  |  |  |  |  |  |
| Region of England (East Midlands) |  |  |  |  |  |  |  |  |  |  |  |  |
| East of England | -0.02 | -0.24 | 0.19 | 0.767 | 0.01 | -0.21 | 0.23 | 0.930 | -0.01 | -0.20 | 0.17 | 0.855 |
| London | -0.05 | -0.22 | 0.12 | 0.448 | -0.04 | -0.24 | 0.16 | 0.588 | -0.02 | -0.21 | 0.18 | 0.802 |
| North East | 0.02 | -0.21 | 0.25 | 0.796 | 0.02 | -0.23 | 0.26 | 0.845 | 0.02 | -0.27 | 0.31 | 0.836 |
| North West | -0.01 | -0.18 | 0.15 | 0.820 | 0.01 | -0.17 | 0.18 | 0.889 | 0.01 | -0.18 | 0.20 | 0.880 |
| South Central | -0.08 | -0.26 | 0.11 | 0.255 | -0.02 | -0.21 | 0.17 | 0.806 | -0.03 | -0.25 | 0.18 | 0.679 |
| South East | -0.03 | -0.21 | 0.15 | 0.656 | 0.00 | -0.21 | 0.20 | 0.995 | 0.00 | -0.17 | 0.17 | 0.977 |
| South West | -0.04 | -0.20 | 0.13 | 0.538 | 0.00 | -0.19 | 0.19 | 0.993 | -0.01 | -0.21 | 0.19 | 0.940 |
| West Midlands | -0.05 | -0.22 | 0.13 | 0.451 | 0.00 | -0.18 | 0.19 | 0.943 | -0.01 | -0.20 | 0.17 | 0.866 |
| Yorkshire & Humber | -0.02 | -0.23 | 0.18 | 0.743 | 0.00 | -0.22 | 0.23 | 0.979 | 0.00 | -0.23 | 0.23 | 0.999 |
|  |  |  |  |  |  |  |  |  |  |  |  |  |
| Smoking status (Non-smoker) |  |  |  |  |  |  |  |  |  |  |  |  |
| Ex-smoker | -0.24 | -0.43 | -0.05 | 0.003 | -0.07 | -0.54 | 0.40 | 0.640 | -0.05 | -0.59 | 0.48 | 0.743 |
| Light-smoker | 0.99 | 0.81 | 1.16 | <0.001 | 1.54 | 0.99 | 2.08 | <0.001 | 1.51 | 0.84 | 2.19 | <0.001 |
| Moderate-smoker | 1.37 | 0.68 | 2.06 | <0.001 | 1.40 | -0.02 | 2.83 | 0.011 | 2.09 | 0.05 | 4.13 | 0.009 |
| Heavy-smoker | 0.86 | 0.02 | 1.69 | 0.009 | 2.55 | -0.45 | 5.54 | 0.020 | 3.13 | 0.61 | 5.64 | 0.004 |
|  |  |  |  |  |  |  |  |  |  |  |  |  |
| Alcohol use (Non-drinker/trivial) |  |  |  |  |  |  |  |  |  |  |  |  |
| Light | -0.14 | -0.37 | 0.09 | 0.084 | -0.07 | -0.48 | 0.34 | 0.568 | -0.48 | -0.81 | -0.14 | 0.002 |
| Medium | -0.04 | -0.40 | 0.31 | 0.695 | 0.18 | -0.62 | 0.97 | 0.448 | -0.14 | -1.07 | 0.80 | 0.606 |
| Heavy | 0.05 | -0.45 | 0.56 | 0.746 | 0.60 | -1.21 | 2.41 | 0.278 | -0.04 | -2.04 | 1.95 | 0.937 |
| Very heavy | 0.20 | -0.61 | 1.02 | 0.436 | 0.22 | -1.03 | 1.47 | 0.567 | -0.11 | -2.55 | 2.32 | 0.863 |
|  |  |  |  |  |  |  |  |  |  |  |  |  |
| Ethnic group (White) |  |  |  |  |  |  |  |  |  |  |  |  |
| African/Caribbean | 1.25 | 0.80 | 1.70 | <0.001 | 1.33 | 0.16 | 2.50 | 0.006 | 0.59 | -1.12 | 2.30 | 0.219 |
| Asian | 1.44 | 1.26 | 1.62 | <0.001 | 0.92 | 0.38 | 1.46 | <0.001 | 0.43 | -0.38 | 1.24 | 0.113 |
| Other | 1.24 | 0.86 | 1.62 | <0.001 | 1.23 | 0.42 | 2.03 | 0.001 | 1.01 | 0.26 | 1.76 | 0.002 |
|  |  |  |  |  |  |  |  |  |  |  |  |  |
| Age | 0.00 | 0.00 | 0.00 | <0.001 | 0.00 | 0.00 | 0.00 | 0.010 | 0.00 | 0.00 | 0.00 | 0.057 |
|  |  |  |  |  |  |  |  |  |  |  |  |  |
| Sex (Female) |  |  |  |  |  |  |  |  |  |  |  |  |
| Male | 0.03 | -0.02 | 0.08 | 0.118 | 0.01 | -0.04 | 0.06 | 0.518 | 0.01 | -0.05 | 0.07 | 0.702 |
|  |  |  |  |  |  |  |  |  |  |  |  |  |
| Year of diagnosis (1998-2005) |  |  |  |  |  |  |  |  |  |  |  |  |
| 2006 to 2010 | 0.01 | -0.06 | 0.08 | 0.816 | -0.01 | -0.09 | 0.06 | 0.646 | -0.01 | -0.09 | 0.07 | 0.788 |
| 2011 to 2015 | -0.03 | -0.10 | 0.04 | 0.312 | -0.02 | -0.13 | 0.08 | 0.522 | -0.02 | -0.10 | 0.05 | 0.379 |
| 2016 to 2020 | 0.05 | -0.02 | 0.12 | 0.046 | 0.00 | -0.11 | 0.10 | 0.923 | 0.00 | -0.09 | 0.08 | 0.878 |
|  |  |  |  |  |  |  |  |  |  |  |  |  |
| Coronary heart disease | 0.09 | -0.14 | 0.32 | 0.268 | 0.03 | -0.12 | 0.19 | 0.579 | 0.04 | -0.14 | 0.22 | 0.586 |
| Stroke | 0.01 | -0.21 | 0.24 | 0.888 | -0.02 | -0.19 | 0.16 | 0.780 | 0.00 | -0.24 | 0.24 | 0.996 |
| Diabetes | 0.02 | -0.10 | 0.14 | 0.699 | 0.03 | -0.14 | 0.19 | 0.654 | 0.00 | -0.15 | 0.15 | 0.967 |
| Epilepsy | 0.00 | -0.20 | 0.19 | 0.951 | 0.02 | -0.22 | 0.26 | 0.811 | 0.00 | -0.27 | 0.28 | 0.975 |
| Hypothyroidism | -0.02 | -0.20 | 0.16 | 0.720 | -0.02 | -0.18 | 0.15 | 0.785 | -0.01 | -0.21 | 0.19 | 0.874 |
| Arthritis | -0.01 | -0.14 | 0.11 | 0.761 | 0.00 | -0.16 | 0.16 | 0.991 | 0.00 | -0.13 | 0.14 | 0.941 |
| Anxiety | 0.00 | -0.07 | 0.08 | 0.966 | 0.01 | -0.07 | 0.08 | 0.834 | 0.02 | -0.07 | 0.10 | 0.592 |
| Migraine | 0.02 | -0.09 | 0.12 | 0.670 | 0.01 | -0.14 | 0.17 | 0.806 | 0.00 | -0.11 | 0.12 | 0.970 |
| Cancer | -0.04 | -0.13 | 0.05 | 0.229 | -0.03 | -0.13 | 0.07 | 0.456 | -0.03 | -0.14 | 0.09 | 0.550 |
| Asthma | 0.00 | -0.07 | 0.07 | 0.990 | 0.01 | -0.07 | 0.10 | 0.684 | 0.00 | -0.08 | 0.09 | 0.900 |
| Renal failure | -0.02 | -0.41 | 0.36 | 0.871 | -0.03 | -0.57 | 0.52 | 0.899 | -0.02 | -0.66 | 0.62 | 0.935 |
| Liver failure | 0.04 | -0.21 | 0.29 | 0.674 | 0.03 | -0.28 | 0.35 | 0.784 | 0.01 | -0.32 | 0.34 | 0.944 |
| Osteoporosis | 0.00 | -0.21 | 0.21 | 0.999 | 0.03 | -0.23 | 0.29 | 0.762 | 0.00 | -0.29 | 0.29 | 0.977 |
| Suicidality | 0.08 | -0.13 | 0.28 | 0.316 | 0.06 | -0.10 | 0.22 | 0.328 | 0.05 | -0.12 | 0.22 | 0.406 |
| Antihypertensive | -0.01 | -0.12 | 0.09 | 0.787 | -0.02 | -0.15 | 0.11 | 0.715 | -0.02 | -0.16 | 0.13 | 0.766 |
| Aspirin | 0.01 | -0.15 | 0.17 | 0.878 | 0.04 | -0.13 | 0.22 | 0.500 | 0.02 | -0.13 | 0.17 | 0.730 |
| Anticoagulants | -0.03 | -0.30 | 0.25 | 0.798 | -0.01 | -0.29 | 0.28 | 0.948 | 0.03 | -0.20 | 0.26 | 0.732 |
| NSAIDs | 0.12 | -0.05 | 0.28 | 0.064 | 0.06 | -0.10 | 0.22 | 0.309 | 0.04 | -0.16 | 0.24 | 0.587 |
| Anticonvulsants | 0.08 | -0.16 | 0.32 | 0.381 | 0.03 | -0.22 | 0.28 | 0.748 | 0.05 | -0.26 | 0.36 | 0.679 |
| Hypnotics | 0.06 | -0.06 | 0.17 | 0.197 | 0.03 | -0.09 | 0.15 | 0.459 | 0.01 | -0.12 | 0.15 | 0.772 |
| Bisphosphonates | -0.05 | -0.46 | 0.36 | 0.755 | 0.02 | -0.44 | 0.48 | 0.906 | 0.06 | -0.44 | 0.55 | 0.746 |
| Contraceptives | -0.09 | -0.24 | 0.05 | 0.078 | -0.06 | -0.17 | 0.06 | 0.199 | -0.04 | -0.15 | 0.08 | 0.427 |
| *Constant* | 2.00 | 1.58 | 2.42 | <0.001 | 3.27 | 2.19 | 4.35 | <0.001 | 4.93 | 3.82 | 6.04 | <0.001 |

## A11. Subgroup analysis on sample >65 years old

### **Table A11a.** Characteristics of subgroup sample >65 years old at baseline. Values are numbers (percentages) unless stated otherwise

| **Characteristic** | **Antidepressant-only**  **N 59,124** | **Antidepressant**  **+statin**  **N 25,857** | **All sample**  **N 84,981** |
| --- | --- | --- | --- |
| Sex |  |  |  |
| Male | 22,120 (37.41) | 12,699 (49.11) | 34,819 (40.97) |
| Mean age [SD] | 78.44 (8.44) | 77.77 (7.58) | 78.24 (8.19) |
| Read codes for depression |  |  |  |
| Major depression | 38,799 (65.62) | 14,240 (55.07) | 53,039 (62.41) |
| Minor depression | 18,728 (31.68) | 10,783 (41.70) | 29,511 (34.73) |
| Other | 1,597 (2.70) | 834 (3.23) | 2,431 (2.86) |
| PHQ-9 baseline [SD] | 14.92 (5.18) | 14.93 (5.24) | 14.92 (5.20) |
| Year of diagnosis |  |  |  |
| 1998 to 2005 | 23,656 (40.01) | 3,753 (14.51) | 27,409 (32.25) |
| 2006 to 2010 | 12,403 (20.98) | 6,772 (26.19) | 19,175 (22.56) |
| 2011 to 2015 | 11,951 (20.21) | 7,774 (30.07) | 19,725 (23.21) |
| 2016 to 2020 | 11,114 (18.80) | 7,558 (29.23) | 18,672 (21.97) |
| Body Mass Index [SD] | 26.15 (5.31) | 27.47 (5.49) | 26.59 (5.41) |
| Smoking |  |  |  |
| Non-smoker | 28,571 (48.32) | 12,282 (47.50) | 40,853 (48.07) |
| Ex-smoker | 16,231 (27.45) | 9,319 (36.04) | 25,550 (30.07) |
| Light-smoker | 7,642 (12.93) | 3,316 (12.82) | 10,958 (12.89) |
| Moderate-smoker | 368 (0.62) | 110 (0.43) | 478 (0.56) |
| Heavy-smoker | 171 (0.29) | 60 (0.23) | 231 (0.27) |
| Alcohol (daily units) |  |  |  |
| Non-drinker/trivial (< 1) | 16,681 (28.21) | 9,156 (35.41) | 25,837 (30.40) |
| Light (1-2) | 9,035 (15.28) | 4,692 (18.15) | 13,727 (16.15) |
| Medium (3-6) | 2,446 (4.14) | 1,514 (5.86) | 3,960 (4.66) |
| Heavy (7-9) | 275 (0.47) | 174 (0.67) | 449 (0.53) |
| Very heavy (> 9) | 226 (0.38) | 108 (0.42) | 334 (0.39) |
| Ethnic group |  |  |  |
| White | 35,183 (59.51) | 19,170 (74.14) | 54,353 (63.96) |
| Indian | 309 (0.52) | 308 (1.19) | 617 (0.73) |
| Pakistani | 158 (0.27) | 182 (0.70) | 340 (0.40) |
| Bangladeshi | 96 (0.16) | 130 (0.50) | 226 (0.27) |
| Other Asian | 148 (0.25) | 145 (0.56) | 293 (0.34) |
| Caribbean | 285 (0.48) | 240 (0.93) | 525 (0.62) |
| Black African | 109 (0.18) | 67 (0.26) | 176 (0.21) |
| Chinese | 40 (0.07) | 28 (0.11) | 68 (0.08) |
| Other | 303 (0.51) | 202 (0.78) | 505 (0.59) |
| Townsend deprivation score in fifths |  |  |  |
| 1 (Least deprived) | 18,031 (30.50) | 7,352 (28.43) | 25,383 (29.87) |
| 2 | 15,375 (26.00) | 6,439 (24.90) | 21,814 (25.67) |
| 3 | 12,180 (20.60) | 5,294 (20.47) | 17,474 (20.56) |
| 4 | 8,341 (14.11) | 4,110 (15.90) | 12,451 (14.65) |
| 5 (Most deprived) | 5,055 (8.55) | 2,631 (10.18) | 7,686 (9.04) |
| Region of England |  |  |  |
| East Midlands | 1,787 (3.02) | 661 (2.56) | 2,448 (2.88) |
| East of England | 2,993 (5.06) | 1,069 (4.13) | 4,062 (4.78) |
| London | 7,381 (12.48) | 3,782 (14.63) | 11,163 (13.14) |
| North-East | 1,725 (2.92) | 872 (3.37) | 25,97 (3.06) |
| North-West | 11,831 (20.01) | 6,214 (24.03) | 18,045 (21.23) |
| South-Central | 8,223 (13.91) | 3,041 (11.76) | 11264 (13.25) |
| South-East | 7,575 (12.81) | 3,023 (11.69) | 10,598 (12.47) |
| South-West | 8,226 (13.91) | 2,903 (11.23) | 11,129 (13.10) |
| West Midlands | 6,583 (11.13) | 3,037 (11.75) | 9,620 (11.32) |
| Yorkshire & Humber | 2,800 (4.74) | 1,255 (4.85) | 4,055 (4.77) |
| Comorbidities at baseline |  |  |  |
| Coronary heart disease | 7,015 (11.86) | 9,381 (36.28) | 16,396 (19.29) |
| Stroke | 6,451 (10.91) | 6,362 (24.60) | 12,813 (15.08) |
| Diabetes | 5,566 (9.41) | 7,223 (27.93) | 12,789 (15.05) |
| Epilepsy | 886 (1.50) | 513 (1.98) | 1,399 (1.65) |
| Hypothyroidism | 5,074 (8.58) | 2,816 (10.89) | 7,890 (9.28) |
| Arthritis | 17,657 (29.86) | 8,858 (34.26) | 26,515 (31.20) |
| Anxiety | 6,670 (11.28) | 2,868 (11.09) | 9,538 (11.22) |
| Migraine | 2,006 (3.39) | 1,032 (3.99) | 3,038 (3.57) |
| Cancer | 15,252 (25.80) | 6,918 (26.75) | 22,170 (26.09) |
| Asthma | 10,491 (17.74) | 5,086 (19.67) | 15,577 (18.33) |
| Renal failure | 763 (1.29) | 597 (2.31) | 1,360 (1.60) |
| Liver failure | 638 (1.08) | 425 (1.64) | 1,063 (1.25) |
| Osteoporosis | 4,490 (7.59) | 1,745 (6.75) | 6,235 (7.34) |
| Suicidality | 481 (0.81) | 224 (0.87) | 705 (0.83) |
| Antidepressant category at baseline |  |  |  |
| SSRIs | 44,388 (75.08) | 20,103 (77.75) | 64,491 (75.89) |
| TCAs | 7,506 (12.70) | 1,721 (6.66) | 9,227 (10.86) |
| MAOIs | 13 (0.02) | 2 (0.01) | 15 (0.02) |
| other ADs | 7,217 (12.21) | 4,031 (15.59) | 1,1248 (13.24) |
| Use of other drugs at baseline |  |  |  |
| Antihypertensive | 12,622 (21.35) | 14,006 (54.17) | 26,628 (31.33) |
| Aspirin | 9,293 (15.72) | 10,856 (41.98) | 20,149 (23.71) |
| Anticoagulants | 3,196 (5.41) | 2,943 (11.38) | 6,139 (7.22) |
| NSAIDs | 3,827 (6.47) | 1,117 (4.32) | 4,944 (5.82) |
| Anticonvulsants | 1,711 (2.89) | 1,240 (4.80) | 2,951 (3.47) |
| Hypnotics | 7,873 (13.32) | 2,926 (11.32) | 10,799 (12.71) |
| Bisphosphonates | 1,220 (2.06) | 585 (2.26) | 1,805 (2.12) |
| Contraceptives | 3 (0.01) | 3 (0.01) | 6 (0.01) |

### **Table A11b.** Unadjusted and adjusted odds ratios (any statin + antidepressant vs antidepressant-only) for acceptability and tolerability at 2 months, 6 months, and 12 months; subgroup sample >65 years old

|  |  | **COMPLETE CASE ANALYSIS** | | | | | | **FULL SET ANALYSIS** | | | | | | |
| --- | --- | --- | --- | --- | --- | --- | --- | --- | --- | --- | --- | --- | --- | --- |
|  |  | Unadjusted analysis  N= 84,981 | | | Adjusted analysis  N= 6,091 | | | Unadjusted analysis  N= 84,981 | | | Adjusted analysis  N= 84,981 | | | |
| Outcome | Time  point | *OR* | *99% CI* | *P* | *OR* | *99% CI* | *P* | *OR* | *99% CI* | *P* | *OR* | *99% CI* | *P* | |
| **Acceptability** | 2mo | 0.82 | 0.79 to 0.85 | <0.001 | 0.83 | 0.70 to 0.99 | 0.006 | 0.82 | 0.79 to 0.85 | <0.001 | 0.89 | 0.85 to 0.93 | <0.001 | |
|  | 6mo  12mo | 0.76  0.71 | 0.73 to 0.79  0.67 to 0.74 | <0.001  <0.001 | 0.82  0.75 | 0.69 to 0.97  0.61 to 0.92 | 0.002  <0.001 | 0.76  0.71 | 0.73 to 0.79  0.67 to 0.74 | <0.001  <0.001 | 0.85  0.81 | 0.81 to 0.89  0.78 to 0.87 | <0.001  <0.001 |  |
| **Tolerability** | 2mo | 0.93 | 0.87 to 0.99 | 0.002 | 0.90 | 0.67 to 1.22 | 0.381 | 0.93 | 0.87 to 0.99 | 0.002 | 0.92 | 0.86 to 0.99 | 0.006 | |
|  | 6mo  12mo | 0.97  1.06 | 0.92 to 1.02  1.01 to 1.11 | 0.089  0.001 | 0.98  1.03 | 0.76 to 1.25  0.83 to 1.27 | 0.800  0.757 | 0.97  1.06 | 0.92 to 1.02  1.01 to 1.11 | 0.089  0.001 | 0.94  0.98 | 0.88 to 1.00  0.93 to 1.04 | 0.011  0.476 | |

### **Table A11c.** Unadjusted and adjusted odds ratios (any statin + antidepressant vs antidepressant-only) for safety (any adverse event, self-harm, suicidality, completed suicide, and all-cause mortality) at 2 months, 6 months, and 12 months; subgroup sample >65 years old

|  |  | **COMPLETE CASE ANALYSIS** | | | | | | **FULL SET ANALYSIS** | | | | | |
| --- | --- | --- | --- | --- | --- | --- | --- | --- | --- | --- | --- | --- | --- |
|  |  | Unadjusted analysis  N= 84,981 | | | Adjusted analysis  N= 6,091 | | | Unadjusted analysis  N= 84,981 | | | Adjusted analysis  N= 84,981 | | |
| Outcome | Time  point | *OR* | *99% CI* | *P* | *OR* | *99% CI* | *P* | *OR* | *99% CI* | *P* | *OR* | *99% CI* | *P* |
| **Any adverse event** | 2mo | 1.10 | 1.05 to 1.15 | <0.001 | 1.00 | 0.85 to 1.19 | 0.943 | 1.10 | 1.05 to 1.15 | <0.001 | 0.98 | 0.94 to 1.03 | 0.367 |
|  | 6mo  12mo | 1.17  1.19 | 1.12 to 1.23  1.12 to 1.26 | <0.001  <0.001 | 0.92  0.91 | 0.77 to 1.10  0.75 to 1.11 | 0.245  0.227 | 1.17  1.19 | 1.12 to 1.23  1.12 to 1.26 | <0.001  <0.001 | 0.99  0.97 | 0.94 to 1.05  0.91 to 1.03 | 0.725  0.168 |
| **Self-harm** | 2mo | 0.59 | 0.33 to 1.05 | 0.018 | 0.00 | 0.00 to 1374.98 | 0.083 | 0.59 | 0.33 to 1.05 | 0.018 | 0.61 | 0.29 to 1.26 | 0.080 |
|  | 6mo  12mo | 0.67  0.71 | 0.41 to 1.09  0.45 to 1.13 | 0.036  0.057 | 0.05  0.06 | 0.00 to 1.56  0.00 to 0.92 | 0.025 0.008 | 0.67  0.71 | 0.41 to 1.09  0.45 to 1.13 | 0.036  0.057 | 0.65  0.73 | 0.35 to 1.20  0.41 to 1.28 | 0.071  0.150 |
| **Suicidality** | 2mo | 1.53 | 1.16 to 2.02 | <0.001 | 0.87 | 0.39 to 1.96 | 0.659 | 1.53 | 1.16 to 2.02 | <0.001 | 1.15 | 0.83 to 1.59 | 0.275 |
|  | 6mo  12mo | 1.45  1.39 | 1.15 to 1.83  1.22 to 1.73 | <0.001  <0.001 | 0.73  0.67 | 0.34 to 1.56  0.33 to 1.34 | 0.284  0.135 | 1.45  1.39 | 1.14 to 1.83  1.12 to 1.73 | <0.001  <0.001 | 1.14  1.13 | 0.87 to 1.49  0.88 to 1.44 | 0.208  0.201 |
| **Completed suicide** | 2mo | 0.57 | 0.07 to 4.39 | 0.480 | - |  |  | - |  |  | 1.77 | 0.13 to 24.52 | 0.577 |
|  | 6mo  12mo | 0.43  0.69 | 0.09 to 1.99  0.22 to 2.17 | 0.155  0.400 | -  - |  |  | -  - |  |  | 0.82  1.59 | 0.13 to 5.06  0.38 to 6.63 | 0.774  0.400 |
| **All-cause mortality** | 2mo | 0.71 | 0.64 to 0.78 | <0.001 | 0.61 | 0.32 to 1.17 | 0.051 | 0.71 | 0.64 to 0.78 | <0.001 | 0.69 | 0.62 to 0.78 | <0.001 |
|  | 6mo  12mo | 0.71  0.80 | 0.64 to 0.78  0.76 to 0.85 | <0.001  <0.001 | 0.61  0.70 | 0.32 to 1.17  0.52 to 0.96 | 0.051  0.003 | 0.71  0.80 | 0.64 to 0.78  0.76 to 0.85 | <0.001  <0.001 | 0.69  0.71 | 0.62 to 0.78  0.66 to 0.76 | <0.001  <0.001 |

### **Table A11d.** Unadjusted and adjusted odds ratios/mean differences (any statin + antidepressant vs antidepressant-only) for efficacy (response, remission, change in depressive score), as measured on the PHQ-9 at 2 months, 6 months, and 12 months; subgroup sample >65 years old

|  |  | **COMPLETE CASE ANALYSIS** | | | | | | | | **FULL SET ANALYSIS** | | | | | |
| --- | --- | --- | --- | --- | --- | --- | --- | --- | --- | --- | --- | --- | --- | --- | --- |
|  |  | Unadjusted analysis  N= see below | | | | Adjusted analysis  N= see below | | | | Unadjusted analysis  N= 84,981 | | | Adjusted analysis  N= 84,981 | | |
| Outcome | Time  point | *N* | *OR* | *99% CI* | *P* | *N* | *OR* | *99% CI* | *P* | *OR* | *99% CI* | *P* | *OR* | *99% CI* | *P* |
| **Response** | 2mo | 3,592 | 1.06 | 0.88 to 1.27 | 0.452 | 2,019 | 1.09 | 0.82 to 1.45 | 0.446 | 1.00 | 0.91 to 1.09 | 0.872 | 1.04 | 0.99 to 1.09 | 0.024 |
|  | 6mo  12mo | 576  326 | 1.16  1.08 | 0.77 to 1.77  0.57 to 2.04 | 0.351  0.768 | 330  171 | 1.02  1.86 | 0.40 to 2.63  0.43 to 8.10 | 0.947  0.279 | 0.99  0.99 | 0.92 to 1.07  0.93 to 1.05 | 0.721  0.565 | 1.03  0.99 | 0.98 to 1.08  0.94 to 1.04 | 0.104  0.706 |
| **Remission** | 2mo | 5,570 | 0.99 | 0.83 to 1.18 | 0.864 | 2,019 | 1.26 | 0.91 to 1.75 | 0.062 | 1.02 | 0.92 to 1.12 | 0.605 | 1.05 | 0.98 to 1.13 | 0.049 |
|  | 6mo  12mo | 1,119  695 | 1.41  1.26 | 0.99 to 2.03  0.79 to 2.00 | 0.013  0.204 | 319  152 | 1.12  1.68 | 0.40 to 3.15  0.34 to 8.20 | 0.784  0.402 | 1.01  1.00 | 0.92 to 1.11  0.91 to 1.09 | 0.720  0.953 | 1.04  1.02 | 0.97 to 1.10  0.96 to 1.09 | 0.131  0.391 |
|  |  | *N* | *MD* | *99% CI* | *P* | *N* | *MD* | *99% CI* | *P* | *MD* | *99% CI* | *P* | *MD* | *99% CI* | *P* |
| **Change** | 2mo | 3,592 | -0.17 | -0.70 to 0.35 | 0.390 | 2,020 | -0.25 | -0.97 to 0.47 | 0.366 | -0.08 | -0.20 to 0.35 | 0.421 | -0.01 | -0.25 to 0.28 | 0.901 |
| **in depression**  **score** | 6mo  12mo | 576  326 | -0.07  0.18 | -1.46 to 1.32  -2.12 to 2.48 | 0.901  0.839 | 332  178 | 0.01  -1.36 | -2.30 to 2.32  -4.62 to 1.89 | 0.988  0.276 | -0.09  -0.09 | -0.19 to 0.37  -0.13 to 0.31 | 0.367  0.250 | -0.01  -0.02 | -0.30 to 0.28  -0.20 to 0.23 | 0.897  0.840 |

## A12. Comparison with aspirin

### **A12a.** Background evidence for antidepressant+aspirin use for depression

Small trials investigating acetylsalicylic acid (aspirin, a very commonly used anti-inflammatory and anti-platelet drug) in addition to antidepressant treatments sometimes showed a positive effect on depressive symptoms (Sepehrmanesh et al., 2017), sometimes no antidepressant effect but indeed more adverse events (Berk et al., 2020). Larger observational studies even suggested an increased association (OR = 1.10, 95% CI = 1.05 to 1.16) between the use of aspirin alone and depressive episodes (Kim et al., 2020).

Perhaps unsurprisingly, we could not identify any study that, akin to ours, explicitly assessed the acceptability and tolerability of antidepressant treatment in addition to aspirin. Our regression analyses did include data about aspirin (see Supplementary Material, S10), since it had been examined as a potential confounder. Overall, no significative effect for aspirin could be observed on acceptability and efficacy outcomes. Tolerability and safety (any adverse event and all-cause mortality) appeared worse. The general impression, compounded by more consistently positive clinical trials and meta-analyses (De Giorgi et al., 2021), is thus that statins may have an overall better profile for repurposing in depression. However, these observations should be considered with great caution since our study had not been conceived for assessing the effects of medications other than statins along with antidepressants.

### **Table A12b.** Summary of regression analyses for aspirin

CCA: complete case analysis; FSA: full set analysis

Acceptability

CCA

2 months: no effect

6 months: no effect

12 months: no effect

FSA

2 months: no effect

6 months: no effect

12 months: no effect

Tolerability

CCA

2 months: no effect

6 months: no effect

12 months: no effect

FSA

2 months: no effect

6 months: negative effect (OR = 1.09, 95% CI = 1.03 to 1.16, P<0.001)

12 months: negative effect (OR = 1.08, 95% CI = 1.03 to 1.14, P<0.001)

Any adverse event

CCA

2 months: no effect

6 months: no effect

12 months: no effect

FSA

2 months: negative effect (OR = 1.07, 95% CI = 1.07 to 1.11, P<0.001)

6 months: negative effect (OR = 1.12, 95% CI = 1.07 to 1.17, P<0.001)

12 months: negative effect (OR = 1.18, 95% CI = 1.12 to 1.24, P<0.001)

Self-harm

CCA

2 months: no effect

6 months: no effect

12 months: no effect

FSA

2 months: no effect

6 months: no effect

12 months: no effect

Suicidality

CCA

2 months: no effect

6 months: no effect

12 months: no effect

FSA

2 months: no effect

6 months: no effect

12 months: no effect

Completed suicide

CCA

2 months: no effect

6 months: no effect

12 months: no effect

FSA

2 months: no effect

6 months: no effect

12 months: no effect

All-cause mortality

CCA

2 months: no effect

6 months: no effect

12 months: no effect

FSA

2 months: no effect

6 months: no effect

12 months: negative effect (OR = 1.15, 95% CI = 1.07 to 1.24, P<0.001)

Response

CCA

2 months: no effect

6 months: no effect

12 months: no effect

FSA

2 months: no effect

6 months: no effect

12 months: no effect

Remission

CCA

2 months: no effect

6 months: no effect

12 months: no effect

FSA

2 months: no effect

6 months: no effect

12 months: no effect

Change in depression score

*CCA*

2 months: no effect

6 months: no effect

12 months: no effect

*FSA*

2 months: no effect

6 months: no effect

12 months: no effect

## A13. STROBE checklist

STROBE Statement—checklist of items that should be included in reports of observational studies

|  | Item No. | Recommendation | Page  No. | Relevant text from manuscript |
| --- | --- | --- | --- | --- |
| **Title and abstract** | 1 | (*a*) Indicate the study’s design with a commonly used term in the title or the abstract | 1 |  |
|  |  | (*b*) Provide in the abstract an informative and balanced summary of what was done and what was found | 3-4 |  |
| Introduction | | | |  |
| Background/rationale | 2 | Explain the scientific background and rationale for the investigation being reported | 5 |  |
| Objectives | 3 | State specific objectives, including any prespecified hypotheses | 5 |  |
| Methods | | | |  |
| Study design | 4 | Present key elements of study design early in the paper | 6 |  |
| Setting | 5 | Describe the setting, locations, and relevant dates, including periods of recruitment, exposure, follow-up, and data collection | 6 |  |
| Participants | 6 | (*a*) *Cohort study*—Give the eligibility criteria, and the sources and methods of selection of participants. Describe methods of follow-up  *Case-control study*—Give the eligibility criteria, and the sources and methods of case ascertainment and control selection. Give the rationale for the choice of cases and controls  *Cross-sectional study*—Give the eligibility criteria, and the sources and methods of selection of participants | 6 |  |
|  |  | (*b*) *Cohort study*—For matched studies, give matching criteria and number of exposed and unexposed  *Case-control study*—For matched studies, give matching criteria and the number of controls per case | - |  |
| Variables | 7 | Clearly define all outcomes, exposures, predictors, potential confounders, and effect modifiers. Give diagnostic criteria, if applicable | 6-7 |  |
| Data sources/ measurement | 8* | For each variable of interest, give sources of data and details of methods of assessment (measurement). Describe comparability of assessment methods if there is more than one group | 7 |  |
| Bias | 9 | Describe any efforts to address potential sources of bias | 7 |  |
| Study size | 10 | Explain how the study size was arrived at | 7 |  |

Continued on next page

| Quantitative variables | 11 | Explain how quantitative variables were handled in the analyses. If applicable, describe which groupings were chosen and why | 7 |  |
| --- | --- | --- | --- | --- |
| Statistical methods | 12 | (*a*) Describe all statistical methods, including those used to control for confounding | 7 |  |
|  |  | (*b*) Describe any methods used to examine subgroups and interactions | 7 |  |
|  |  | (*c*) Explain how missing data were addressed | 7 |  |
|  |  | (*d*) *Cohort study*—If applicable, explain how loss to follow-up was addressed  *Case-control study*—If applicable, explain how matching of cases and controls was addressed  *Cross-sectional study*—If applicable, describe analytical methods taking account of sampling strategy | 7 |  |
|  |  | (*e*) Describe any sensitivity analyses | 7-8 |  |
| Results | | | | |
| Participants | 13* | (a) Report numbers of individuals at each stage of study—eg numbers potentially eligible, examined for eligibility, confirmed eligible, included in the study, completing follow-up, and analysed | 9 |  |
|  |  | (b) Give reasons for non-participation at each stage | 9 |  |
|  |  | (c) Consider use of a flow diagram | 9 | Figure 1 |
| Descriptive data | 14* | (a) Give characteristics of study participants (eg demographic, clinical, social) and information on exposures and potential confounders | 9 | Table 1 |
|  |  | (b) Indicate number of participants with missing data for each variable of interest | 9 | Supplementary Material S4 |
|  |  | (c) *Cohort study*—Summarise follow-up time (eg, average and total amount) | - |  |
| Outcome data | 15* | *Cohort study*—Report numbers of outcome events or summary measures over time | 9-10 | Table 2, 3, 4; Supplementary Material S5, S6, S7 |
|  |  | *Case-control study—*Report numbers in each exposure category, or summary measures of exposure | - |  |
|  |  | *Cross-sectional study—*Report numbers of outcome events or summary measures | - |  |
| Main results | 16 | (*a*) Give unadjusted estimates and, if applicable, confounder-adjusted estimates and their precision (eg, 95% confidence interval). Make clear which confounders were adjusted for and why they were included | 9-10 | Table 2, 3, 4; Supplementary Material S5, S6, S7 |
|  |  | (*b*) Report category boundaries when continuous variables were categorized | - |  |
|  |  | (*c*) If relevant, consider translating estimates of relative risk into absolute risk for a meaningful time period | - |  |

Continued on next page

| Other analyses | 17 | Report other analyses done—eg analyses of subgroups and interactions, and sensitivity analyses | 10 | Supplementary Material S8, S9 |
| --- | --- | --- | --- | --- |
| Discussion | | | | |
| Key results | 18 | Summarise key results with reference to study objectives | 11 |  |
| Limitations | 19 | Discuss limitations of the study, taking into account sources of potential bias or imprecision. Discuss both direction and magnitude of any potential bias | 12 |  |
| Interpretation | 20 | Give a cautious overall interpretation of results considering objectives, limitations, multiplicity of analyses, results from similar studies, and other relevant evidence | 11 |  |
| Generalisability | 21 | Discuss the generalisability (external validity) of the study results | 11,13 |  |
| Other information | |  | | |
| Funding | 22 | Give the source of funding and the role of the funders for the present study and, if applicable, for the original study on which the present article is based | 1 |  |

*Give information separately for cases and controls in case-control studies and, if applicable, for exposed and unexposed groups in cohort and cross-sectional studies.

**Note:** An Explanation and Elaboration article discusses each checklist item and gives methodological background and published examples of transparent reporting. The STROBE checklist is best used in conjunction with this article (freely available on the Web sites of PLoS Medicine at http://www.plosmedicine.org/, Annals of Internal Medicine at http://www.annals.org/, and Epidemiology at http://www.epidem.com/). Information on the STROBE Initiative is available at www.strobe-statement.com
